# Mechanical Caging of Nucleic Acids Enabled by Light‐Activated Synthetic Molecular Motors

**DOI:** 10.1002/anie.4279044

**Published:** 2026-08-23

**Authors:** Yuchen Ma, Erika McCarthy, N. Sanjeeva Murthy, Cameron McGovern, Zhenyu Han, Şölen Ekesan, Subhajit Nandi, Advik Sirimarapu, Wilma K. Olson, Darrin M. York, Yuwei Gu

**Affiliations:** ^1^ Department of Chemistry and Chemical Biology Rutgers The State University of New Jersey Piscataway New Jersey USA; ^2^ Laboratory For Biomolecular Simulation Research Rutgers The State University of New Jersey Piscataway New Jersey USA; ^3^ Institute For Quantitative Biomedicine Rutgers The State University of New Jersey Piscataway New Jersey USA; ^4^ Department of Chemistry and International Institute For Nanotechnology Northwestern University Evanston Illinois USA; ^5^ Center For Quantitative Biology Rutgers The State University of New Jersey Piscataway New Jersey USA

**Keywords:** DNA structures, molecular dynamics, nonequilibrium processes, nucleic acid regulation, supramolecular chemistry

## Abstract

Control over nucleic acid activity is central to biotechnology and therapeutic development. Most existing strategies rely on installing protecting groups that mask nucleobases or backbone functionalities (i.e., chemical caging), where modulation of activity arises from alteration of the biomolecule's chemical identity. Here, inspired by biological mechanisms such as DNA supercoiling that regulate nucleic acid function through mechanically imposed conformational constraints, we introduce mechanical caging as an alternative strategy. A light‐driven synthetic molecular motor was integrated with single‐stranded DNA through four tethering points, creating a topology that couples motor rotation to DNA conformation. Control experiments confirmed that motor incorporation preserves intrinsic properties of ssDNA, including its ability to hybridize with complementary strands. Upon photoactivation, unidirectional motor rotation drives DNA into constrained conformations that suppress hybridization and reduce susceptibility to exonuclease digestion. Molecular dynamics simulations further provide molecular‐level insight into how motor‐driven mechanical constraints may impact the conformational ensemble and functional accessibility of nucleic acids. Incorporation of acid‐labile tethers enables on‐demand motor detachment to restore the native conformational ensemble and DNA function. Together, these results establish an externally controlled mechanical caging–decaging cycle and introduce a strategy for controlling biomolecular function through mechanical input rather than chemical masking.

## Graphical abstract

A synthetic light‐driven molecular motor imposes mechanical constraints on nucleic acids, enabling “mechanical caging” as a new mode to regulate DNA structure and function through mechanical input rather than chemical masking.

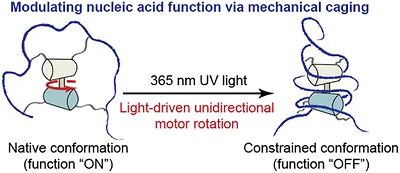

## 1 Introduction

The ability to exert on‐demand control over the functions of biomacromolecules is central to modern biotechnology [1, 2]. In the context of nucleic acids, most current strategies rely on chemical caging approaches [3]. For example, incorporation of photoresponsive protecting groups such as *o*‐nitrobenzyl [4], or thermoresponsive protecting groups such as glyoxal [5], into oligonucleotides enables chemical masking of functional sites and modulation of nucleic acid properties. More recently, acylation of the 2′‐OH group has emerged as an effective strategy for transient RNA cloaking [6, 7]. These chemical caging approaches have enabled powerful applications in biosensing [8, 9], gene editing [10, 11], and transcriptional regulation [12]. However, because regulation is achieved by altering the molecule's chemical identity, these approaches offer limited dynamic control and may introduce unintended functional consequences.

In biological systems, although nature has also evolved strategies analogous to chemical caging, such as DNA and RNA methylation [13], nucleic acid activity is often regulated through mechanically driven conformational control [14, 15, 16]. A prominent example is the supercoiling of double‐stranded DNA [17, 18], in which enzymes such as condensins and topoisomerases, together with histone‐mediated chromatin organization, impose topological constraints that modulate DNA accessibility and activity [19, 20, 21, 22, 23, 24, 25, 26]. Inspired by these biological mechanisms, we sought to develop a synthetic strategy that regulates nucleic acid function by imposing mechanical constraints on molecular conformation. We term this strategy *mechanical caging*, in which activity is regulated by globally remodeling the conformational ensemble rather than through localized molecular modifications. Beyond establishing a fundamentally different mode of regulation, such large‐scale conformational control may expand the scope of biomolecular regulation and enable applications beyond the reach of conventional chemical‐caging strategies.

Realizing this concept requires a synthetic actuator capable of generating controlled motion at the molecular scale. Advances in supramolecular chemistry have established synthetic molecular machines as systems capable of such motion [27, 28, 29, 30]. Among these, rotary molecular motors—including the light‐driven motors developed by Feringa and coworkers—undergo repetitive unidirectional rotation upon energy input [29, 31, 32, 33, 34]. When mechanically coupled to appropriately designed macromolecular architectures, this rotary motion can be converted into mechanical work, enabling stimulus‐driven actuation of materials [35, 36]. Despite these advances, the use of synthetic molecular motors has remained largely confined to small‐molecule and synthetic polymer systems [37, 38, 39], leaving it unclear whether this mechanical output can be harnessed to regulate the structure and function of biomacromolecules.

Here, we report the first example of an artificial, light‐driven molecular motor that actively imposes mechanical constraints on single‐stranded DNA (ssDNA) to regulate its function (Figure 1a,b). We demonstrate that motor activation drives ssDNA into a conformationally constrained state, resulting in functional deactivation through suppression of hybridization with a complementary strand and reduced susceptibility to exonuclease digestion. Molecular dynamics (MD) simulations further elucidate how motor rotation reshapes the conformational ensemble and functional accessibility of DNA. Finally, we present a fully reversible system in which the imposed constraints can be released on demand, completing a mechanical caging–decaging cycle for a biomacromolecule.

**FIGURE 1:**
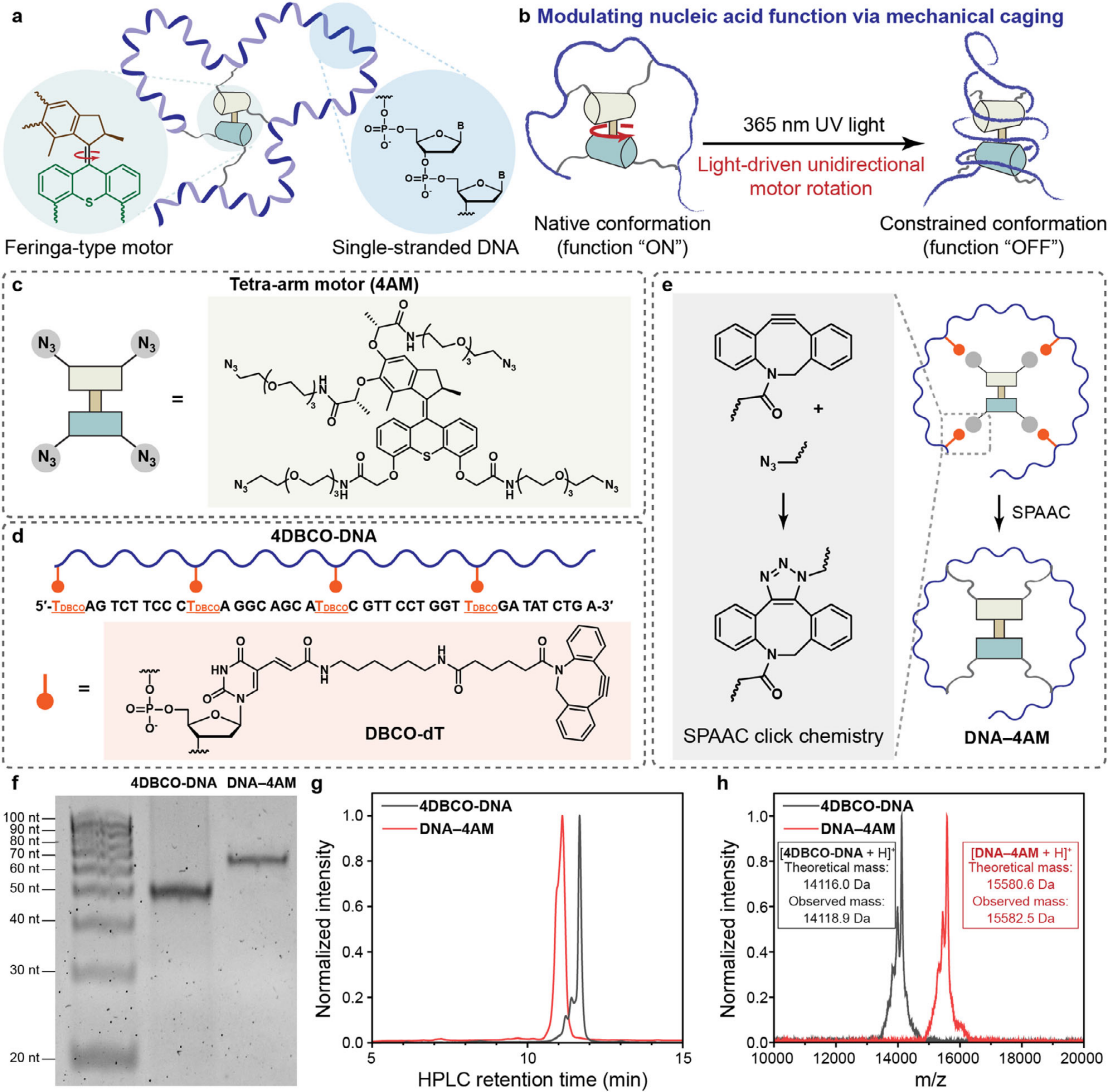
Conceptual illustration and molecular design of mechanical caging of ssDNA enabled by a synthetic molecular motor. (a) Schematic illustration of a covalent conjugate in which ssDNA is tethered to a Feringa‐type molecular motor through four anchoring points. Upon irradiation at 365 nm, the rotor (brown) undergoes unidirectional rotation relative to the stator (green). (b) Illustration of the proposed mechanical caging strategy, in which unidirectional motor rotation is expected to drive the DNA strand into a mechanically constrained state that suppresses its functional accessibility. (c) Chemical structure of the tetra‐arm motor (**4AM**), in which both the rotor and stator bear two azide‐functionalized side arms. (d) Sequence of a 40‐nt ssDNA strand (**4DBCO‐DNA**) bearing four evenly spaced DBCO groups. (e) Formation of **DNA–4AM** via SPAAC reactions between **4AM** and **4DBCO‐DNA**. (f) Denaturing PAGE (20%, GelRed‐stained) analysis of **4DBCO‐DNA** and **DNA–4AM**. A 20–100 nt ssDNA ladder is shown for size reference. An uncropped image of the original gel is shown in Figure S44. (g) Reversed‐phase HPLC analysis (detected at 254 nm) of **4DBCO‐DNA** and **DNA–4AM**. (h) MALDI‐MS analysis confirming formation of **DNA–4AM**.

## 2 Results and Discussion

### 2.1 Conjugation of DNA to a Tetra‐Arm Motor

To enable mechanical modulation of nucleic acid conformation, we designed a motor–DNA conjugate (**DNA–4AM**) in which a light‐driven rotary motor is tethered to ssDNA through four anchoring points (Figure 1a). Unlike conventional chemical caging, the motor is attached to ssDNA not to directly modify DNA function, but to establish a topology that mechanically couples motor rotation to nucleic acid conformation, allowing the motor to impose conformational constraints on the DNA strand. The motor structure (**4AM**) is based on a second‐generation Feringa‐type core (Figure 1c), whose light‐driven unidirectional rotation has been extensively characterized [32]. Here, we harness this established rotary motion as a molecular actuator to impose mechanical constraints on DNA.

To construct the conjugate, we synthesized a 40‐nucleotide ssDNA bearing four evenly spaced dibenzocyclooctyne (DBCO) groups (**4DBCO‐DNA**) via incorporation of DBCO‐dT phosphoramidites during automated solid‐phase synthesis (Figure 1d). Installation of DBCO at the C5 position of thymidine preserves Watson‐–Crick recognition [40] while enabling efficient strain‐promoted azide–alkyne cycloaddition (SPAAC) [41] with the azide‐functionalized motor, yielding **DNA–4AM** (Figure 1e). The pyrimidine‐rich sequence (Table S1) was selected to minimize intrinsic secondary structure so that structural or functional changes upon photoactivation would arise from motor‐induced mechanical constraints rather than preexisting folding equilibria. The 40‐nt length provides a representative model of ssDNA while remaining short enough that motor‐induced mechanical caging can be clearly detected through structural and functional assays.

**DNA–4AM** was obtained in high yield by incubating **4DBCO‐DNA** (10 µM) with **4AM** (30 µM) in 0.3 M triethylammonium acetate (TEAA) buffer. Denaturing urea polyacrylamide gel electrophoresis (PAGE) and high‐performance liquid chromatography (HPLC) each showed a distinct band or peak corresponding to the conjugate (Figure 1f,g). Matrix‐assisted laser desorption/ionization mass spectrometry (MALDI‐MS) further confirmed successful conjugation (Figure 1h): the parent **4DBCO‐DNA** exhibited a major peak at *m/z* 14118.9, whereas **DNA–4AM** showed a single peak at *m/z* 15582.5, corresponding to the expected mass increase upon conjugation with one **4AM** molecule. Incubation with excess small‐molecule azide produced no additional mass shift, verifying complete consumption of all four DBCO groups (Figure S1). These results confirm formation of the motor–DNA conjugate with four covalent linkages.

Because the azide groups on **4AM** and the DBCO groups on **4DBCO‐DNA** are chemically equivalent, conjugation affords a mixture of topological isomers that differ in the connectivity of the motor relative to the DNA strand. Detailed analysis of the possible connectivities reveals three distinct topological arrangements (Figures S2 and S3). Two correspond to “figure‐of‐eight” architectures previously shown to support motor‐induced mechanical constraints in synthetic polymer systems [35, 37, 38]. Although the third does not adopt a classical figure‐of‐eight arrangement, dissipation of imposed mechanical strain would require an intramolecular threading of a substantial DNA segment through a sterically confined loop (Figure S4), an event that is highly improbable in a macromolecular context. Accordingly, all topological variants are, in principle, capable of sustaining motor‐induced mechanical constraints. Unless otherwise noted, subsequent experiments were performed on the mixture, and a single representative topology is shown in Figure 1e for clarity.

### 2.2 Motor‐Driven Mechanical Constraints and Functional Deactivation of DNA

To determine whether motor activation mechanically imposes conformational constraints on **DNA–4AM**, an aqueous solution (6 µM) was irradiated at 365 nm for 2 h. Denaturing PAGE revealed that the irradiated sample migrated more rapidly than the nascent conjugate (Figure 2a), consistent with formation of a more compact conformational state. Notably, this mobility shift persisted under denaturing conditions, indicating that the structural change arises from mechanically imposed constraints resistant to disruption by agents that typically interfere with intramolecular interactions, such as hydrogen bonding.

**FIGURE 2:**
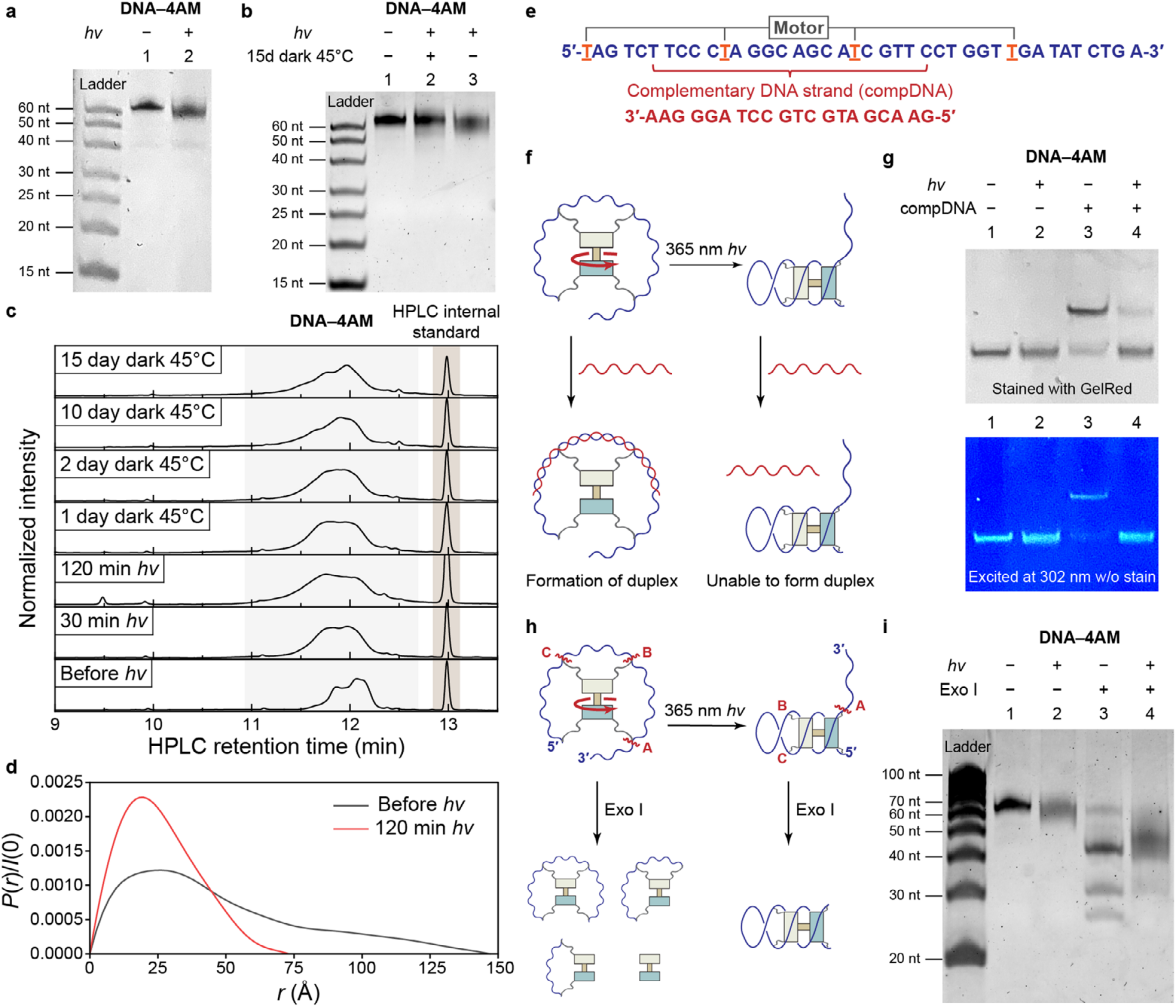
Motor‐driven mechanical caging suppresses DNA function. (a) Denaturing PAGE (20%, GelRed‐stained) analysis of **DNA–4AM** before and after irradiation. Lane 1: nascent **DNA–4AM**; Lane 2: **DNA–4AM** after 365 nm irradiation for 2 h. A 10–60 nt ssDNA ladder is shown for size reference. An uncropped image of the original gel is shown in Figure S45. (b) Denaturing PAGE (20%, GelRed‐stained) analysis of thermal relaxation of irradiated **DNA–4AM**. Lane 1: nascent **DNA–4AM**; Lane 2: post‐irradiated **DNA–4AM** incubated at 45°C in the dark for 15 days; Lane 3: **DNA–4AM** after irradiation at 365 nm for 2 h. A 10–60 nt ssDNA ladder is shown for size reference. An uncropped image of the original gel is shown in Figure S46. (c) Reversed‐phase HPLC analysis (detected at 254 nm) of **DNA–4AM** during 365 nm irradiation and subsequent incubation at 45°C in the dark. (d) SAXS‐derived pair distance distribution functions, *P*(*r*), of nascent and post‐irradiated **DNA–4AM**. Curves are normalized to the zero‐angle scattering intensity, *I*(0). (e) Sequence of the 20‐nt complementary strand (compDNA) and its fully complementary region within **DNA–4AM**. (f) Schematic illustration of how mechanical constraints may reduce hybridization accessibility of **DNA–4AM**. (g) Native PAGE (20%) analysis of hybridization between **DNA–4AM** and compDNA. Lane 1: nascent **DNA–4AM**; Lane 2: irradiated **DNA–4AM** (365 nm, 2 h); Lane 3: nascent **DNA–4AM** incubated with compDNA in 0.2 M NaCl at room temperature overnight; Lane 4: irradiated **DNA–4AM** incubated with compDNA. Top: GelRed‐stained; bottom: imaged via intrinsic fluorescence of the motor (302 nm excitation). Uncropped images of the original gels are shown in Figures S47 and S48. (h) Schematic illustration of reduced accessibility of irradiated **DNA–4AM** to Exo I. (i) Denaturing PAGE (20%, GelRed‐stained) analysis of Exo I digestion. Lane 1: nascent **DNA–4AM**; Lane 2: irradiated **DNA–4AM** (365 nm, 2 h); Lane 3: nascent **DNA–4AM** treated with Exo I at 37 °C for 2 h; Lane 4: irradiated **DNA–4AM** treated with Exo I. A 20–100 nt ssDNA ladder is shown for size reference. An uncropped image of the original gel is shown in Figure S49.

Interestingly, incubation at 45°C in the dark resulted in a slow, partial recovery toward the original mobility profile (Figure 2b). Similar partial relaxation has previously been reported for mechanically constrained Feringa‐type motors [37, 38]. In these systems, mechanical strain accumulated during light‐driven rotation provides the driving force for thermal back‐rotation after irradiation ceases. As the stored strain is progressively released, the driving force for further back‐rotation decreases until it is balanced by the intrinsic thermal barrier of the motor, leaving the system in a partially relaxed state rather than fully restoring the initial conformation.

Analytical HPLC further supported this conformational change. Using a slow‐gradient method (Supporting Information: Section I), **DNA–4AM** resolved into a defined peak profile that progressively shifted to shorter retention time upon irradiation (Figure 2c), consistent with altered hydrodynamic and conformational properties. Time‐course measurements showed that most of the change occurred within the first 90 min and reached a steady state after approximately 2 h (Figure S8). Thermal incubation in the dark partially restored the initial chromatographic profile, in agreement with the PAGE results.

Small‐angle x‐ray scattering (SAXS) provided direct insight into the light‐induced conformational change. Guinier analysis revealed a decrease in the radius of gyration (*R*_g_) from 2.41 nm to 1.93 nm following irradiation (Figures S10 and S11, Tables S2 and S3), consistent with global compaction. An indirect Fourier transform of the scattering data yields the pair distance distribution function [42], *P*(*r*), which describes the probability of finding intramolecular atom pairs separated by a distance *r*. Upon irradiation, the *P*(*r*) profile shifted toward shorter distances (Figure 2d), indicating a redistribution of conformations toward more constrained states. Kratky analysis further revealed reduced conformational flexibility in the irradiated sample (Figures S10 and S11). Together, these data demonstrate that motor activation drives **DNA–4AM** into a more compact and conformationally restricted state.

We next evaluated whether the mechanically constrained state induced by motor activation alters DNA function. Because duplex formation requires accessibility of nucleobases, we hypothesized that the imposed mechanical constraints would suppress hybridization (Figure 2f). A 20‐nt complementary strand (compDNA) was synthesized to target a region of **DNA–4AM** expected to experience the greatest motor‐induced mechanical constraint (Figure 2e). In native PAGE assays, nascent **DNA–4AM** readily formed duplexes with compDNA, as evidenced by the appearance of a slower‐migrating band (Figure 2g, Lane 3). In contrast, the irradiated sample showed minimal duplex formation under identical conditions (Figure 2g, Lane 4), indicating that motor activation suppresses hybridization by restricting strand accessibility. The intrinsic fluorescence of the motor enabled unambiguous quantification of duplex and single‐stranded species.

Another critical property of ssDNA is its susceptibility to degradation by single‐strand‐specific nucleases. Because efficient enzymatic processing requires conformational accessibility of the substrate, we examined whether motor‐induced mechanical constraints enhance resistance to nuclease‐mediated degradation (Figure 2h). We employed Exonuclease I (Exo I), which selectively digests ssDNA in the 3′→5′ direction. After incubating nascent **DNA–4AM** with Exo I at 37°C for 2 h, denaturing PAGE revealed three distinct bands corresponding to degradation intermediates (Figure 2i, Lane 3). This pattern is consistent with partial obstruction at the anchoring sites of the motor (labeled A, B, and C in Figure 2h), which impedes enzyme progression. Nevertheless, the presence of progressively truncated products indicates that the conformationally flexible nascent **DNA–4AM** remains largely accessible to digestion.

In contrast, Exo I treatment of the irradiated sample produced a broad band centered approximately 10 nucleotides shorter than the untreated control (Figure 2i, Lane 4 vs. Lane 2). This result suggests that only the segment between the 3′ end and anchoring point A remains accessible, whereas the remainder of **DNA–4AM** becomes conformationally restricted upon motor activation. As a result, Exo I digestion stalls near anchoring point A, preventing further degradation. To more clearly visualize this mechanically caged state, we synthesized an analogous **DNA–4AM** construct lacking the 3′ overhang (i.e., the segment between anchoring site A and the 3′ terminus). While the nascent construct was readily cleaved by Exo I, the UV‐irradiated construct remained intact following Exo I treatment, retaining a single, well‐defined band corresponding to the full‐length construct (Figure S12). These higher‐resolution results further confirm that motor‐induced mechanical caging strongly suppresses Exo I digestion.

Notably, all the experiments described above were performed using **DNA–4AM** as the unresolved mixture of topological isomers. To experimentally validate our prediction that all three topologies are capable of sustaining mechanical caging, we optimized the HPLC separation conditions to resolve the individual topological isomers (Figure S5). Hybridization and Exo I assays performed on each isolated isomer showed comparable suppression of hybridization and enhanced nuclease resistance following irradiation (Figure S6), confirming that mechanical caging is a general property of all three topological isomers rather than being specific to a particular motor–DNA connectivity.

Collectively, these structural and functional analyses establish that light activation of the tetra‐arm motor mechanically drives **DNA–4AM** into a compact, conformationally constrained state that suppresses hybridization and reduces nuclease susceptibility. These findings provide the foundation for our mechanical caging strategy and establish the first example of transient DNA deactivation achieved through conformational constraints imposed by mechanical input from a fully synthetic molecular system.

### 2.3 Validation of the Motor‐Driven Mechanical Caging Mechanism

While **DNA–4AM** exhibits pronounced structural and functional changes upon irradiation, it is essential to exclude alternative photochemical processes that could account for these observations. We first evaluated the photostability of the **DNA–4AM** conjugate by monitoring the intrinsic fluorescence of its molecular motor during continuous UV irradiation under the same aqueous, aerobic conditions used for mechanical caging. As shown in Figure 3a, the fluorescence spectra remained essentially unchanged throughout the 2 h irradiation period. Because photooxidation of overcrowded alkenes is typically accompanied by fluorescence loss [43], the absence of detectable spectral changes indicates negligible motor degradation within the **DNA–4AM** conjugate during the experimental irradiation window. This observation is consistent with the reported high photostability of second‐generation Feringa‐type molecular motors, even under aqueous conditions [43]. A pronounced decrease in fluorescence was observed only after overnight irradiation, well beyond the irradiation period employed throughout this work. We next evaluated the photostability of the DNA itself. A motor‐free ssDNA strand was subjected to 365 nm irradiation for 2 h. No detectable changes were observed in analytical HPLC, hybridization assays, or Exo I digestion (Figure S16). These results are consistent with the minimal structural perturbation expected under short‐term, low‐dose UVA exposure [44]. Together, these results demonstrate that the structural and functional changes observed for **DNA–4AM** do not arise from photodegradation of either the molecular motor or the DNA strand.

**FIGURE 3:**
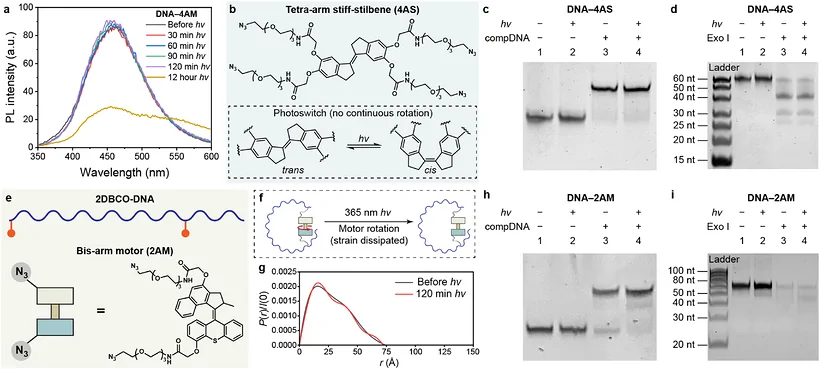
Control experiments validate the motor‐driven mechanism of mechanical caging. (a) Fluorescence emission spectra of **DNA–4AM** (25 µM in H_2_O; *λ*_ex_ = 330 nm) recorded after different UV irradiation times. (b) Structure of the tetra‐arm stiff‐stilbene control (**4AS**), which functions as a reversible photoswitch undergoing *trans*–*cis* photoisomerization but cannot undergo continuous unidirectional rotation. (c) Native PAGE (20%, GelRed‐stained) analysis of hybridization between **DNA–4AS** and compDNA. Lane 1: nascent **DNA–4AS**; Lane 2: irradiated **DNA–4AS** (365 nm, 1 h); Lane 3: nascent **DNA–4AS** incubated with compDNA in 0.2 M NaCl at room temperature overnight; Lane 4: irradiated **DNA–4AS** incubated with compDNA. An uncropped image of the original gel is shown in Figure S50. (d) Denaturing PAGE (20%, GelRed‐stained) analysis of Exo I digestion. Lane 1: nascent **DNA–4AS**; Lane 2: irradiated **DNA–4AS** (365 nm, 1 h); Lane 3: nascent **DNA–4AS** treated with Exo I at 37 °C for 2 h; Lane 4: irradiated **DNA–4AS** treated with Exo I. A 10–60 nt ssDNA ladder is shown for size reference. An uncropped image of the original gel is shown in Figure S51. (e) Structure of a 40‐nt DNA strand bearing two DBCO groups (**2DBCO‐DNA**) and the corresponding bis‐arm molecular motor (**2AM**), in which both the rotor and stator each bear a single azide‐functionalized side arm. (f) Schematic illustration showing that motor rotation in **DNA–2AM** is accommodated by local flexibility of the linkers and DNA backbone, thereby preventing the buildup of sustained mechanical strain. (g) SAXS‐derived *P*(*r*) of nascent and irradiated **DNA–2AM**. Curves are normalized to *I*(0). (h) Native PAGE (20%, GelRed‐stained) analysis of hybridization between **DNA–2AM** and compDNA. Lane 1: nascent **DNA–2AM**; Lane 2: irradiated **DNA–2AM** (365 nm, 2 h); Lane 3: nascent **DNA–2AM** incubated with compDNA in 0.2 M NaCl at room temperature overnight; Lane 4: irradiated **DNA–2AM** incubated with compDNA. An uncropped image of the original gel is shown in Figure S47. (i) Denaturing PAGE (20%, GelRed‐stained) analysis of Exo I digestion. Lane 1: nascent **DNA–2AM**; Lane 2: irradiated **DNA–2AM** (365 nm, 2 h); Lane 3: nascent **DNA–2AM** treated with Exo I at 37 °C for 2 h; Lane 4: irradiated **DNA–2AM** treated with Exo I. A 20–100 nt ssDNA ladder is shown for size reference. An uncropped image of the original gel is shown in Figure S52.

A second critical question is whether the observed DNA caging requires continued unidirectional motor rotation beyond the first half‐turn. If the motor undergoes only a single 180° photoisomerization, the observed conformational changes could, in principle, also be achieved using a conventional molecular photoswitch [45]. Although direct characterization of the rotational state of the motor within **DNA–4AM** is challenging, three independent observations strongly support continued rotation beyond the half‐turn. First, our calculation based on the reported photophysical parameters of the second‐generation Feringa motor indicates that the irradiation conditions employed in this work are sufficient to support multiple complete rotations of the isolated motor during the experimental time window (Supporting Information: Section VI). Second, the time‐dependent UV–Vis spectral evolution of **DNA–4AM** (Figure S7) closely resembles that reported for a second‐generation molecular motor used to wind a synthetic polymer, where detailed molecular‐level characterization established continued unidirectional rotation through at least one and a half turns [37, 38]. Finally, we synthesized a control construct in which the molecular motor was replaced with a tetra‐arm stiff‐stilbene photoswitch (**DNA–4AS**), which undergoes reversible *trans*–*cis* photoisomerization but lacks the ability to generate continuous unidirectional rotation (Figure 3b). In contrast to **DNA–4AM**, **DNA–4AS** exhibited no detectable changes in HPLC (Figure S15b), hybridization (Figure 3c), or Exo I assays (Figure 3d) following irradiation, demonstrating that a single 180° photoisomerization is insufficient to induce mechanical caging. Collectively, these complementary lines of evidence support continued unidirectional motor rotation as the origin of the mechanical caging observed in **DNA–4AM**.

A final critical question is whether continued motor rotation alone is sufficient to deactivate DNA function, or whether the motor must also generate sustained mechanical constraints. To address this question, we examined a control DNA–motor system in which motor rotation is preserved but mechanical caging is not expected to occur. We synthesized a bis‐arm motor construct (**2AM**) that retains the same motor core as **4AM** but is tethered to DNA through only two anchoring points (Figure 3e). Reaction with a DNA strand (**2DBCO‐DNA**) bearing two DBCO groups yielded the corresponding **DNA–2AM** conjugate, whose purity was confirmed by analytical HPLC (Figure S17a), denaturing PAGE (Figure S17b), and MALDI‐MS (Figure S18a).

In **DNA–2AM**, motor rotation can be accommodated by local flexibility along the linkers and DNA backbone without generating sustained mechanical strain (Figure 3f). This mode of rotational accommodation has previously been demonstrated in analogous circular small‐molecule motor systems [46]. Consistent with this expectation, irradiation of **DNA–2AM** at 365 nm for 2 h produced no detectable conformational change. SAXS revealed nearly identical *R*_g_ values for nascent and irradiated samples (2.06 nm vs. 2.08 nm), with essentially superimposable pair distance distribution functions (Figure 3g) and Kratky plots (Figure S10). Denaturing PAGE (Figure S18b) and analytical HPLC (Figure S18c) likewise showed no discernible differences between the two states. Functionally, both nascent and irradiated **DNA–2AM** efficiently formed duplexes with compDNA (Figure 3h). Similarly, Exo I digestion proceeded to completion for both samples (Figure 3i). These results indicate that motor activation alone does not affect hybridization efficiency or nuclease susceptibility in the absence of sustained mechanical constraints. Collectively, these validation experiments show that transient DNA deactivation is not a consequence of photochemical degradation or simple photoisomerization, but instead requires continued unidirectional motor rotation coupled with sustained mechanical constraints.

### 2.4 Structural and Thermodynamic Basis of Motor‐Induced DNA Caging From MD Simulations

While SAXS, HPLC, and PAGE provide strong experimental evidence for conformational constraints in the mechanically caged state, the highly dynamic nature of ssDNA limits detailed structural characterization. To gain molecular‐level insight into the conformational changes underlying motor‐induced DNA deactivation, we performed MD simulations of **DNA–4AM** and the control **DNA–2AM** system to examine how motor rotation may impact the conformational ensemble of the DNA strand. Atomistic models of both constructs were generated by covalently linking a 40‐nt ssDNA strand to the motor core at the appropriate attachment sites, and the systems were parameterized and simulated using AMBER24 [47], following established equilibration protocols [48].

Initially, we performed steered molecular dynamics (SMD) simulations in which rotation of the motor dihedral angle was driven at a constant velocity. All three topological variants of **DNA–4AM** exhibited qualitatively similar behavior: as rotation progressed, increasing resistance to further rotation was observed, consistent with progressive conformational restriction of the DNA strand (Figures S19 and S20). In contrast, **DNA–2AM** exhibited minimal change in rotational resistance over multiple turns (Figures S19 and S20), indicating that motor rotation in this architecture is accommodated without generating sustained conformational constraints on the DNA strand. These simulations qualitatively support the experimental observation that motor activation induces mechanical caging in **DNA–4AM** but not in **DNA–2AM**.

Because SMD simulations are performed under non‐equilibrium conditions and primarily provide qualitative insight into rotational response, we next carried out equilibrium umbrella sampling simulations to more rigorously characterize the free‐energy landscape of the DNA–motor constructs as a function of motor rotation. Umbrella sampling was conducted using 72 windows per full rotation (2π rad), enabling reconstruction of the free‐energy profile along the motor rotation angle. Owing to computational expense, one representative topological isomer of **DNA–4AM** was simulated, as SMD indicated qualitatively similar behavior among all variants. The free‐energy profiles of **DNA–4AM** over three successive rotational cycles are shown in Figure 4a, with replica exchange acceptance ratios provided in Figure S21. Because each cycle was sampled independently, the free energy for each turn is referenced to the onset of that rotation. To isolate DNA‐associated contributions, the free‐energy profile of the isolated motor (**4AM**) was computed separately and used as a baseline. The difference Δ*G* **_DNA–4AM_** − Δ*G* **_4AM_** therefore represents the conformational free‐energy change attributable solely to the attached DNA strand.

**FIGURE 4:**
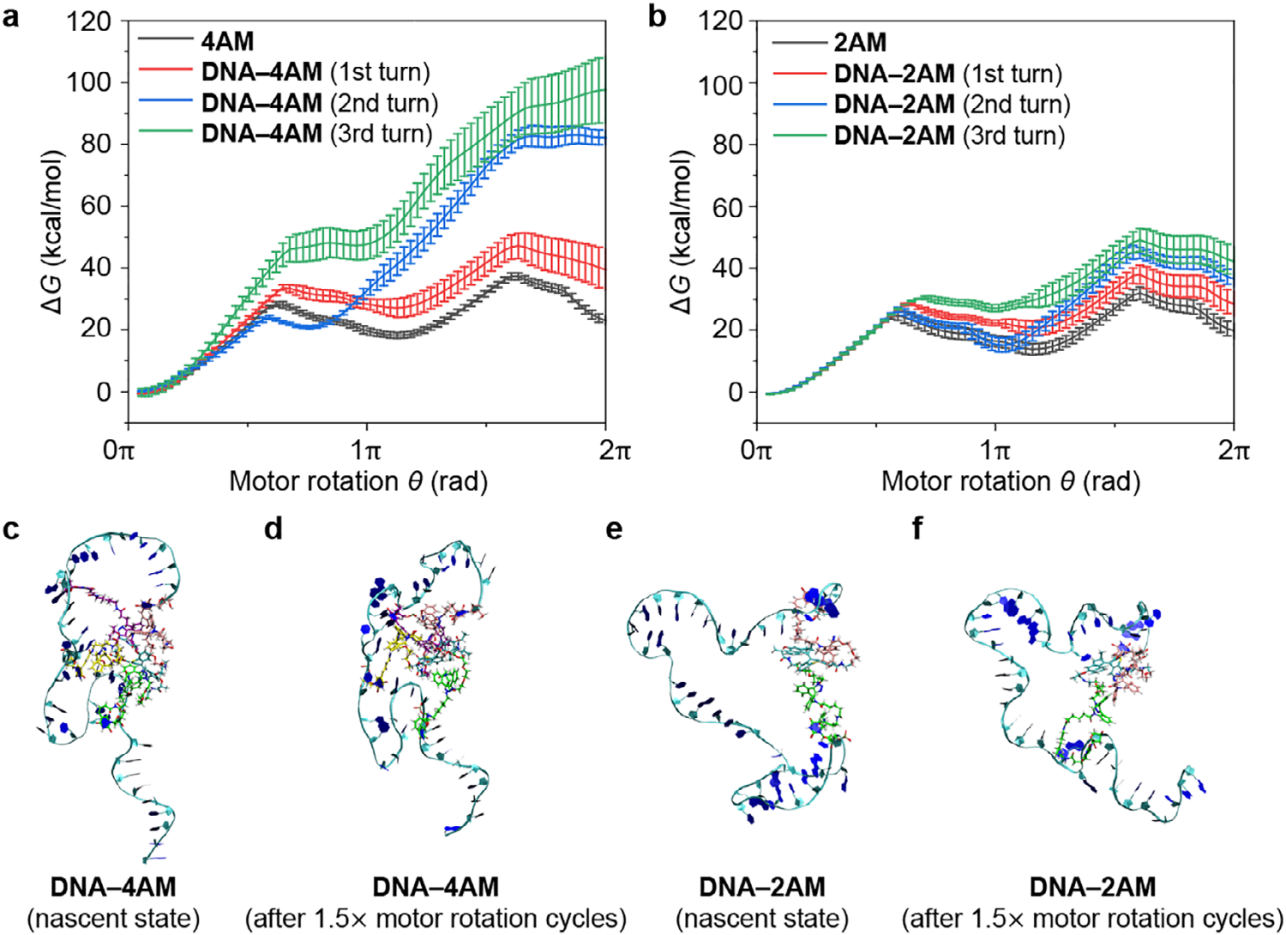
Computational analysis of the structural and thermodynamic basis of motor‐induced DNA caging. (a) Free‐energy profiles of **DNA–4AM** as a function of motor rotation angle (*θ*) over three consecutive rotational cycles, obtained from equilibrium umbrella sampling simulations. Data represent the mean of four independent simulations, with error bars denoting standard deviation. Each cycle was sampled independently, and the free energy for each turn is referenced to the onset of that rotation. The isolated motor (**4AM**) was simulated separately, and its free‐energy profile is shown as a reference. (b) Free‐energy profiles of **DNA–2AM** as a function of motor rotation angle (*θ*) over three consecutive rotational cycles. The free‐energy profile of the isolated motor (**2AM**) is shown as a reference. (c) Representative equilibrated structure of nascent **DNA**–**4AM**. (d) Representative structure of **DNA–4AM** after approximately 1.5 motor rotational cycles (cumulative 3π rad). (e) Representative equilibrated structure of nascent **DNA–2AM**. (f) Representative structure of **DNA–2AM** after approximately 1.5 motor rotational cycles (cumulative 3π rad). All molecular structures were extracted from the corresponding umbrella sampling windows.

During the first rotational cycle, the free‐energy profile of **DNA–4AM** progressively rises above that of the isolated motor, reflecting an increasing conformational free energy of the attached DNA strand. This elevation becomes more pronounced during the second rotational cycle, with a sharp increase around a cumulative rotation angle of 3π rad (Figure 4a). These results indicate that continued unidirectional rotation progressively reshapes the conformational ensemble of the DNA, ultimately driving it into a highly constrained state. The rapid rise in the DNA‐associated free‐energy contribution suggests that the motor reaches a thermodynamic limit after approximately 1.5 rotational cycles (3π rad), as additional rotation would incur a sharply increasing conformational free‐energy penalty from the attached DNA (Figure S22). This estimate is qualitatively consistent with prior studies of motor‐driven molecular winding in small‐molecule model systems [37, 38].

Representative snapshots from the simulations illustrate the structural evolution of the DNA strand following approximately 1.5 rotational cycles of the motor (Figure 4c,d). In the nascent state of **DNA–4AM**, the central segment of the strand remains conformationally flexible and adopts base orientations compatible with hybridization to a complementary strand. After 1.5 motor rotation cycles, pronounced kinking is observed. Quantitative analysis of base stacking overlap areas [49] further reveals a substantial reduction in base stacking relative to the nascent state (Table S4). This transition to a compact, conformationally constrained state reduces nucleobase accessibility and increases steric hindrance for duplex formation, consistent with the experimentally observed suppression of hybridization in the irradiated state.

For comparison, **DNA–2AM** was subjected to equilibrium umbrella sampling under identical conditions. In contrast to **DNA–4AM**, the difference Δ*G* **_DNA–2AM_** − Δ*G* **_2AM_** increases only marginally over the three full rotational cycles examined (Figure 4b), indicating that the conformational free energy of the attached DNA strand does not undergo a significant increase during motor rotation. This behavior is consistent with the topology of **DNA–2AM**, in which motor rotation is accommodated by local flexibility without generating sustained mechanical strain on DNA. Moreover, representative simulation snapshots (Figures 4e,f) show that, unlike **DNA–4AM**, the DNA strand in **DNA–2AM** remains conformationally flexible after approximately 1.5 rotational cycles, with base stacking largely preserved and, in some regions, slightly enhanced (Table S4). The stark contrast between **DNA–4AM** and **DNA–2AM** at both thermodynamic and structural levels provides molecular‐level insight into how mechanical constraints leads to functional deactivation.

### 2.5 Completion of a Mechanical Caging–Decaging Cycle via Cleavable Motor Design

To establish a complete and controllable mechanical caging–decaging cycle, we next designed a motor construct in which the imposed mechanical constraints could be released on demand. While motor activation drives DNA into a constrained state that suppresses function, practical implementation of the mechanical caging strategy requires a reliable mechanism to restore the native state. To this end, we designed a tetra‐arm motor analogue (**4AM‐X**) incorporating acid‐labile acetal linkages (highlighted in red in Figure 5a) on each arm [50]. Cleavage of these linkages under acidic conditions detaches the motor from the DNA strand (Figure 5b), thereby removing the topological constraints responsible for mechanical caging.

**FIGURE 5:**
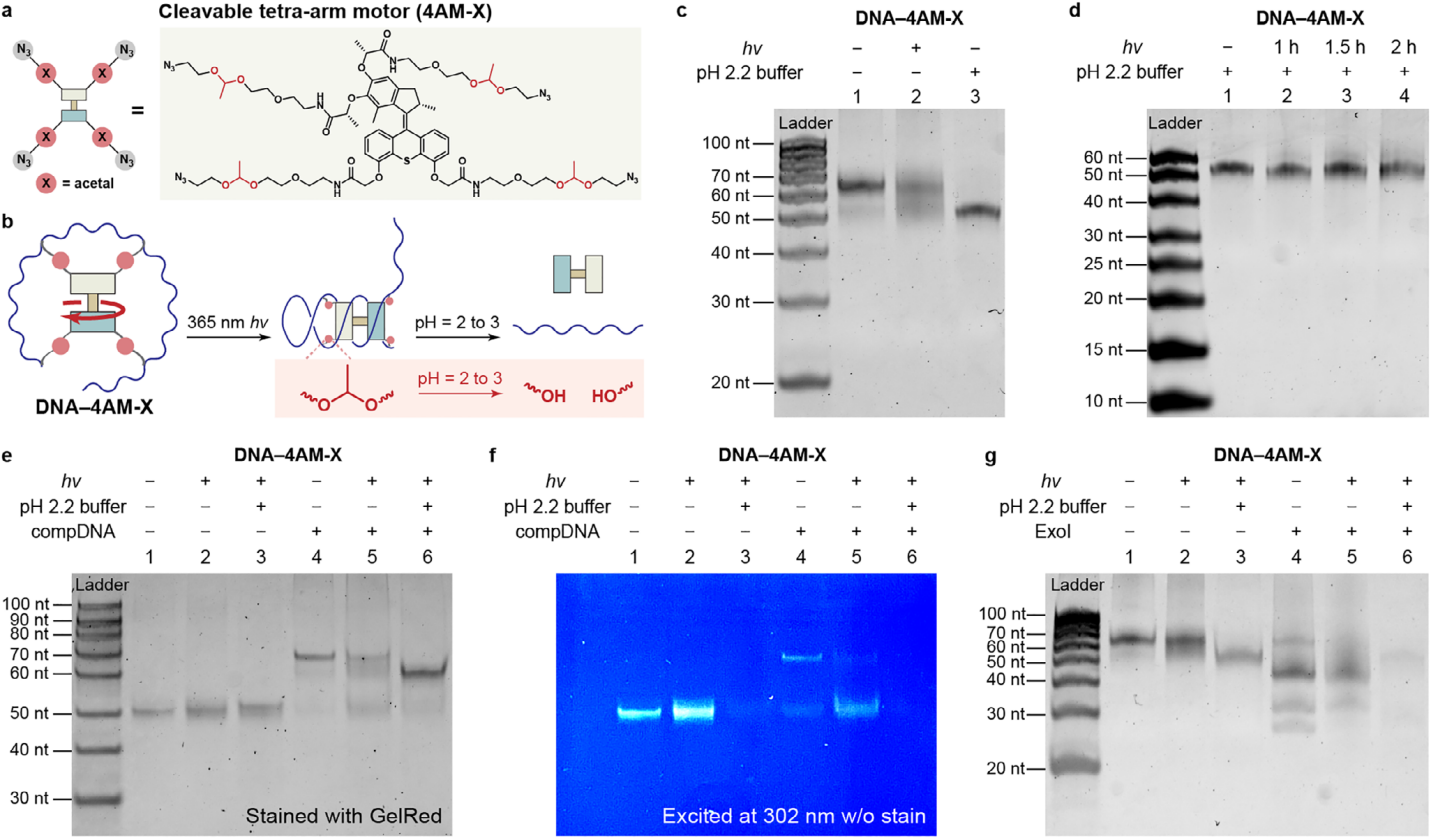
Completion of a mechanical caging–decaging cycle via cleavable motor design. (a) Chemical structure of the cleavable tetra‐arm motor (**4AM‐X**), which incorporates an acid‐labile acetal linkage (red) on each side arm. (b) Schematic illustration of decaging upon acid‐induced cleavage of the acetal linkages, resulting in detachment of the motor from DNA. (c) Denaturing PAGE (20%, GelRed‐stained) analysis of **DNA–4AM‐X** following irradiation and acid treatment. Lane 1: nascent **DNA–4AM‐X**; Lane 2: **DNA–4AM‐X** irradiated at 365 nm for 2 h; Lane 3: nascent **DNA–4AM‐X** incubated in pH 2.2 buffer overnight. A 20–100 nt ssDNA ladder is shown for size reference. An uncropped image of the original gel is shown in Figure S53. (d) Denaturing PAGE (20%, GelRed‐stained) of **DNA–4AM‐X** irradiated for varying durations (1, 1.5, and 2 h at 365 nm) followed by acid treatment. Lane 1: nascent **DNA–4AM‐X** incubated in pH 2.2 buffer overnight; Lanes 2–4: **DNA–4AM‐X** irradiated for 1, 1.5, and 2 h, respectively, then treated with acid. A 10–60 nt ssDNA ladder is shown for size reference. An uncropped image of the original gel is shown in Figure S54. (e) Native PAGE (20%, GelRed‐stained) analysis of hybridization between **DNA–4AM‐X** and compDNA. Lane 1: nascent **DNA–4AM‐X**; Lane 2: irradiated **DNA–4AM‐X**; Lane 3: irradiated **DNA–4AM‐X** treated with acid; Lane 4: nascent **DNA–4AM‐X** incubated with compDNA; Lane 5: irradiated **DNA–4AM‐X** incubated with compDNA; Lane 6: irradiated and acid‐treated **DNA–4AM‐X** incubated with compDNA. A 20–100 nt ssDNA ladder is shown for size reference. An uncropped image of the original gel is shown in Figure S55. (f) The same gel shown in (e) imaged via intrinsic motor fluorescence (302 nm excitation). An uncropped image of the original gel is shown in Figure S56. (g) Denaturing PAGE (20%, GelRed‐stained) analysis of **DNA–4AM‐X** following Exo I digestion. Lane 1: nascent **DNA–4AM‐X**; Lane 2: irradiated **DNA–4AM‐X**; Lane 3: irradiated **DNA–4AM‐X** treated with acid; Lane 4: nascent **DNA–4AM‐X** treated with Exo I; Lane 5: irradiated **DNA–4AM‐X** treated with Exo I; Lane 6: irradiated and acid‐treated **DNA–4AM‐X** treated with Exo I. A 20–100 nt ssDNA ladder is shown for size reference. An uncropped image of the original gel is shown in Figure S57.

The **DNA–4AM‐X** conjugate was synthesized from **4AM‐X** and **4DBCO‐DNA** (Figure S23). Upon 365 nm irradiation, **DNA–4AM‐X** transitioned into a mechanically constrained state analogous to that observed for **DNA–4AM**, as confirmed by denaturing PAGE (Figure 5c, Lane 2 vs. Lane 1) and HPLC analysis (Figure S23d). To evaluate tether cleavability, nascent **DNA–4AM‐X** was incubated overnight in a sodium acetate buffer (pH 2.2). Denaturing PAGE revealed formation of a faster‐migrating species (Figure 5c, Lane 3 vs. Lane 1), consistent with acid‐induced motor detachment and generation of linear ssDNA.

We next examined whether cleavage of the motor from the constrained state enables release of the imposed mechanical constraints. **DNA–4AM‐X** samples were irradiated for varying durations (1–2 h) to induce progressively greater mechanical constraints, followed by overnight incubation at pH 2.2. In all cases, the resulting products exhibited identical denaturing PAGE profiles to those obtained from acid cleavage of the nascent conjugate (Figure 5d), indicating complete removal of the motor and restoration of the same linear ssDNA state. These results demonstrate that the conformational constraints imposed by motor activation are fully reversible upon release of the tether.

Functional restoration was evaluated by hybridization and nuclease assays. Nascent **DNA–4AM‐X** readily formed duplexes with compDNA (Figure 5e, Lane 4 vs. Lane 1). As observed for **DNA–4AM**, the irradiated **DNA–4AM‐X** showed strongly suppressed duplex formation (Figure 5f, Lane 5 vs. Lane 4). Following acid‐triggered motor cleavage, however, the resulting linear ssDNA regained efficient hybridization capability (Figure 5e, Lane 6 vs. Lane 3). Successful motor removal was confirmed by disappearance of intrinsic motor fluorescence (Figure 5f, Lane 3 and Lane 6). Similarly, whereas irradiated **DNA–4AM‐X** displayed enhanced resistance to Exo I digestion, acid‐treated samples recovered full susceptibility to exonuclease degradation (Figure 5g).

Together, these results establish a fully reversible mechanical caging–decaging cycle, in which light‐driven motor activation transiently imposes mechanical constraints to suppress DNA function, and acid‐triggered motor cleavage restores the native, functionally accessible state.

## 3 Conclusion

The ability to regulate biomacromolecular function through synthetic molecular systems remains a central challenge in chemical biology and nanotechnology. In this work, we establish mechanical caging and decaging as a new mode of nucleic acid regulation based on reversible, mechanically imposed conformational constraints. By integrating a light‐driven molecular motor with ssDNA in a topology that sustains mechanical strain, we demonstrate that motor activation drives DNA into a compact, conformationally constrained state that suppresses hybridization and nuclease susceptibility, while programmed removal of the motor fully restores DNA function. At its core, this work demonstrates that the mechanical output of a synthetic molecular motor can be harnessed to reshape the conformational landscape of a biomacromolecule and thereby regulate its function, establishing a conceptual bridge between synthetic molecular machines and biological molecular machinery that exploits mechanical regulation to control biomacromolecular function, as exemplified by DNA supercoiling.

The ability to reversibly impose large‐scale conformational constraints opens new opportunities for regulating biomolecular functions that depend on global molecular conformation. One promising example is DNA aptamers, whose molecular recognition relies on cooperative formation of an extended three‐dimensional binding pocket rather than a single localized interaction [51, 52]. We envision that extending mechanical caging to aptamer sequences would allow motor‐driven conformational constraints to disrupt the binding‐competent structure, thereby suppressing target recognition, while subsequent decaging restores the native fold and recovers function. Given the rapidly expanding use of aptamers in biosensing [53] and targeted therapeutics [54], strategies for precise spatiotemporal regulation of aptamer function are expected to become increasingly important. Mechanical caging may provide a fundamentally new approach to achieving this level of control.

Another exciting opportunity enabled by mechanical caging is the ability to directly apply molecular‐scale mechanical force to biomacromolecules. Such a capability is particularly attractive for DNA nanotechnology, where a longstanding goal is to convert molecular‐scale inputs into controlled motion of DNA‐based nanostructures [55, 56, 57]. Many dynamic DNA nanostructures use flexible ssDNA linkers to connect rigid DNA components [58, 59]. Replacing one such linker with **DNA–4AM** would create a light‐driven contractile element. Motor rotation would shorten the linker, bringing the connected DNA components closer together and thereby converting molecular rotation into nanoscale displacement. Incorporating multiple contractile linkers into larger DNA architectures could further amplify this motion, providing a strategy for integrating synthetic molecular motors as actuators within dynamic DNA nanodevices.

Finally, although mechanical caging is demonstrated here using nucleic acids, the underlying design principle should be broadly applicable to other biomacromolecules. We envision that coupling synthetic molecular motors to peptides and proteins could similarly reshape their conformational landscapes, enabling mechanical regulation of secondary structure, protein folding, and ultimately biological function. In this regard, the present work marks the first step toward harnessing synthetic molecular machines to perform useful mechanical work on biomacromolecules [27, 60].

## Author Contributions

**Yuchen Ma**: writing – review and editing, investigation, formal analysis, data curation, visualization, methodology. **Erika Mccarthy**: methodology, software, data curation, investigation, formal analysis, visualization, writing – review and editing. **N. Sanjeeva Murthy**: investigation, writing – review and editing, data curation, formal analysis. **Cameron Mcgovern**: investigation. **Zhenyu Han**: investigation, writing – review and editing. **Şölen Ekesan**: methodology, software, writing – review and editing. **Subhajit Nandi**: investigation. **Advik Sirimarapu**: investigation. **Wilma K. Olson**: methodology, writing – review and editing, funding acquisition, supervision. **Darrin M. York**: supervision, funding acquisition, writing – review and editing, project administration. **Yuwei Gu**: conceptualization, methodology, investigation, visualization, supervision, funding acquisition, project administration, writing – original draft, writing – review and editing.

## Conflicts of Interest

The authors declare no conflicts of interest.

## Supporting information




**Supporting File**: anie74360‐sup‐0001‐SuppMat.pdf.

## Data Availability

The data that support the findings of this study are available in the Supporting Information of this article.
