# Supplementary material for: Mechanical Caging of Nucleic Acids Enabled by Light‐Activated Synthetic Molecular Motors
Source: Angew Chem Int Ed Engl. 2026 Aug 23;65(42):e4279044. doi: 10.1002/anie.4279044 (PMC13637968; doi:10.1002/anie.4279044)
Supplement: Supplementary file 1 [file ANIE-65-e4279044-s001.pdf]

Supporting Information for

Mechanical Caging of Nucleic Acids Enabled by Light-Activated Synthetic Molecular Motors

Yuchen Ma, Erika McCarthy, N. Sanjeeva Murthy, Cameron McGovern, Zhenyu Han, Şölen Ekesan, Subhajit Nandi, Advik Sirimarapu, Wilma K. Olson, Darrin M. York, Yuwei Gu*

Table of Contents

Section I. Materials and Methods	S3
Section II. Synthetic Procedures	S7
Section III. DNA Sequence	S13
Section IV. MALDI-TOF MS Experiments	S14
Section V. Discussion of Topological Isomers in **DNA–4AM**	S15
Section VI. Estimation of Motor Rotation under the Experimental Irradiation Conditions	S19
Section VII. Photostability of **DNA–4AM**	S20
Section VIII. HPLC and Exo I Analysis of **DNA–4AM**	S21
Section IX. SAXS Characterization of **DNA–4AM** and **DNA–2AM**	S24
Section X. Exo I Assay of the **DNA–4AM** Construct without Overhanging End	S26
Section XI. Studies of the DNA–Stiff-Stilbene Conjugate (**DNA–4AS**)	S27
Section XII. Experiments with **0DBCO-DNA**	S30
Section XIII. HPLC and PAGE Characterization of **DNA–2AM**	S31
Section XIV. Simulations of Motor–DNA Conjugates	S32
Section XV. A Reversible System via Cleavable Tether Design	S35
Section XVI. Spectroscopic Data	S36
Section XVII. Uncropped gel images	S56
SI References	S82

Section I. Materials and Methods

Experimental Materials and Methods

Materials: All deuterated solvents were purchased from Cambridge Isotope Laboratories, Inc. Unless otherwise specified, all other organic reagents, inorganic salts, and solvents were purchased from AmBeed, Millipore Sigma, TCI America, or Fisher Scientific, and used without further purification. All reactions were performed using anhydrous solvents and standard Schlenk techniques under nitrogen. All phosphoramidites, oligonucleotide synthesis reagents, and supplies were purchased from Glen Research and used without further purification.

Thin-layer chromatography was performed on Silica XG TLC Plates (with UV254 fluorescent indicator, aluminum-backed, 200 μm) from Sorbent Technologies, Inc.

Silica gel flash chromatography was performed on standard-grade silica gel (60A, 40-63 μm) from Sorbent Technologies, Inc. using a Teledyne ISCO CombiFlash NextGen 300+ Automated Flash Chromatography System.

^{1}H nuclear magnetic resonance (^{1}H NMR) spectra were acquired on either a 500 MHz Varian VNMRS spectrometer or a 500 MHz Bruker Avance Neo spectrometer. ***^{13}C nuclear magnetic resonance (^{13}C NMR)*** spectra were acquired on a 500 MHz Bruker Avance Neo spectrometer.

High-resolution mass spectrometry (HRMS) was conducted using a Waters Xevo G2-XS QTof LC-MS, equipped with a Waters ACQUITY UPLC BEH C18 Column (1.7 μm, 2.1 × 50 mm) set at 40°C, coupled with a quadrupole-TOF hybrid mass spectrometer. Water with 0.1% formic acid served as mobile phase A, and acetonitrile with 0.1% formic acid was used as mobile phase B. The samples were run with a gradient from 5% B to 95% B over 3.5 minutes at a flow rate of 0.4 mL/min. The MS data were collected in positive ionization mode, and the mass accuracies of the compounds were determined by running a lock spray standard. Data analysis was performed using MassLynx 4.1. The following parameters were employed: ESI capillary voltage = 3.18 kV; sample cone voltage = 30 V; source temperature = 125°C; desolvation temperature = 300°C; cone gas flow = 25 L/h; desolvation gas flow = 600 L/h.

Matrix-assisted laser-desorption ionization time-of-flight mass spectrometry (MALDI-TOF MS) was obtained on a Bruker MALDI Rapiflex Tissue Typer in linear negative mode, with 2′,6′-dihydroxyacetophenone as the matrix and diammonium hydrogen citrate as the co-matrix.

Oligonucleotide synthesis: DNA strands were synthesized on a 1 μmol scale using a K&A H-2 synthesizer equipped with TWIST columns and either Glen UnySupport™ 1000 (for non-DBCO strands) or dA-CPG 1000 (for DBCO-modified strands). Standard ATCG bases were incorporated using dA-CE, dT-CE, Ac-dC-CE, and dmf-dG-CE phosphoramidites. DBCO modification was introduced via DBCO-dT phosphoramidite. Coupling was performed using 0.25 M 5-ethylthio-1H-tetrazole (ETT) in anhydrous acetonitrile as the activator. Deblocking was achieved with 3% trichloroacetic acid in dichloromethane. Capping employed a 1:1 mixture of tetrahydrofuran/pyridine/acetic anhydride (Cap Mix A) and 16% 1-methylimidazole in tetrahydrofuran (Cap Mix B). Oxidation was carried out with 0.02 M iodine in tetrahydrofuran/water/pyridine for non-DBCO strands (40 s) and 0.5 M CSO in anhydrous acetonitrile for DBCO-modified strands (3 min). Coupling times were 55 s for standard bases and 10 min for DBCO-dT. Strands were deprotected and cleaved using 35% aqueous ammonia for 17 h at 55 °C (non-DBCO strands) or 2 h at 65 °C (DBCO strands). A complete list of synthesized oligonucleotides is provided in Supplementary Table S1.

High-performance liquid chromatography (HPLC): All oligonucleotides were purified by reverse-phase HPLC (Shimadzu LC-20AR) using either a ZORBAX StableBond 300 C18 (250 × 9.4 mm, 5 μm) or ZORBAX 300SB-C3 (250 × 9.4 mm, 5 μm) column as the stationary phase. The mobile phase consisted of 0.06 M triethylamine acetate (TEAA) in water (Buffer A) and acetonitrile (Buffer B), with a flow rate of 3 mL/min. Purified oligonucleotides were lyophilized and characterized by MALDI-TOF MS. Analytical HPLC was performed on the same instrument using an InfinityLab Poroshell 120 EC-C18 column (100 × 4.6 mm I.D.) with the same mobile phase and a flow rate of 1 mL/min. Bromocresol green was used as an internal standard for time-course HPLC analysis.

Slow-gradient HPLC elutions were performed at a constant flow rate of 1 mL/min using 0.06 M triethylamine acetate (TEAA) aqueous buffer as mobile phase A and acetonitrile as mobile phase B. For the analysis corresponding to Fig. 2c (23.00 min total run time), the gradient was applied as follows: 0.00–15.00 min, 0–50% B; 15.00–15.50 min, 50–100% B; 15.50–20.00 min, 100% B; 20.00–20.50 min, 100–0% B; 20.50–23.00 min, 0% B. For isomer separation (72.00 min total run time), the gradient was: 0.00–60.00 min, 0–35% B; 60.00–60.50 min, 35–100% B; 60.50–67.50 min, 100% B; 67.50–68.00 min, 100–0% B; 68.00–72.00 min, 0% B. All runs ended with an isocratic hold at 0% B for column re-equilibration.

Polyacrylamide gel electrophoresis (PAGE) was performed using a 20% gel prepared from a 19:1 acrylamide:bis-acrylamide solution (Fisher BioReagents) and run at 120 V for 120 minutes in 1× TBE buffer. Denaturing gels were prepared containing 6 M urea. DNA oligonucleotide length standards (10/60 or 20/100 Ladder, IDT) were used as size markers. After electrophoresis, gels were stained with GelRed (Biotium) and imaged using the Invitrogen iBright Imaging System (Thermo Fisher Scientific). For gels visualized by the intrinsic fluorescence of the motor moiety, imaging was performed using an Accuris SmartDoc™ Imaging System with 302 nm excitation.

Light Irradiation: DNA solutions (10–120 µL, 6 µM in Milli-Q water) were placed in glass vials and irradiated under a UV lamp (Analytik Jena UVP EL, 365 nm, 4 W) positioned 3.2 cm above the sample. The UV intensity at this distance was measured to be 3.7 mW/cm^2 using an AquaHorti UVCBA light meter. To minimize relaxation, irradiated samples were stored at 4 °C prior to measurement.

Exonuclease I (Exo I) digestion assays were performed by incubating 0.1 µg/µL ssDNA with 0.4 U/µL Exo I (New England Biolabs) in 1× Exo I reaction buffer (New England Biolabs) at 37 °C for 2 hours. After incubation, 10 µL of the reaction mixture was mixed with 10 µL of PAGE loading buffer (8 M urea, 0.01% bromophenol blue) and heated at 80 °C for 20 minutes. Samples were then analyzed by denaturing PAGE.

Hybridization assays were conducted by incubating the oligonucleotide (1 µM) with its complementary strand (1 µM) in 0.2 M NaCl at room temperature overnight. Samples were subsequently analyzed by native PAGE.

Small-Angle X-ray Scattering (SAXS) measurements were performed at the 16-ID beamline (LiX), National Synchrotron Light Source II (NSLS-II), Brookhaven National Laboratory (Upton, NY) [1]. **DNA–4AM** or **DNA–2AM** solutions (50 µL), prepared at 1× and 2× dilutions (starting concentration: 50 µM in water), were loaded into 1.5 mm quartz capillaries (Charles Supper) and sealed with silicone grease. Samples were measured at 22 °C. Following initial measurements, the same samples were irradiated with UV light for 2 hours and immediately remeasured by SAXS to obtain post-irradiation data. Data were collected with 15.14 keV X-rays and three Pilatus 1M detectors over a q range of 0.005-3.13 Å^{-1}; the q range of 0.005-0.25 Å^{-1} was used for small-angle data analysis and the data at a q range of 1.5-3.0 Å^{-1}, where water scattering is the dominant feature, were used to scale the buffer and the solution scattering curves. The data were analyzed in BioXTAS RAW-2.3.0 with ATSAS-4.0.1-1 to calculate the radius of gyration (R_g) by Guinier analysis and carry out Kratky analysis; pair-distance distribution functions, $P(r)$, were obtained by indirect Fourier transform with GNOM. [2-4]

UV–Vis spectra were recorded on an Agilent Cary 3500 UV–Vis spectrophotometer equipped with a Multicell Peltier temperature-controlled detector module. The experimental temperature was maintained at 25 °C. The spectra were collected in scan mode over a wavelength range of 250 to 700 nm. The key acquisition parameters were set as follows: a spectral bandwidth (SBW) of 2 nm, a scan rate of 3000 nm/min, a data interval of 1 nm, and an averaging time of 0.02 s.

Fluorescence emission spectra were acquired on an Agilent Cary Eclipse Fluorescence Spectrophotometer. The samples were excited at a wavelength of 330 nm, and the emission spectra were recorded from 350 to 600 nm. Both the excitation and emission slit widths were set to 5 nm. The measurements were performed with a scan rate of 600 nm/min, a data interval of 1.0 nm, and an averaging time of 0.1 s. The photomultiplier tube (PMT) detector voltage was set to

"medium". To improve the signal-to-noise ratio, the spectra were smoothed using a Savitzky-Golay filter (filter size 5).

Computational Methods

Modeling of single stranded DNA (ssDNA) 40-mer

Model structures for ssDNA were created following three different approaches, referred to here as: "straight helix", "histone wrapped", and "**4AM** fragmented". The first approach was to use the 3DNA software [5] to construct a B-DNA helix with the experimental sequence and discard the complementary strand yielding a straight single-stranded helix. The second approach was to use a circular DNA as a template for the sugar backbone and model the nucleobases according to the experimental sequence. A histone-wrapped DNA was used as template (PDB ID 1AOI) [6]. The third approach involved dividing the previously modeled straight helix into four fragments (nucleotides 1-10, 11-19, 20-30, and 31-40), placing them in the vicinity of the motor arms, and pulling together as described in section ***Attachment of the molecular motor to ssDNA***. The motor was then removed to yield ssDNA with DBCO-dT linkages in a highly bent conformation.

Each system was neutralized with 40 Na^+. The straight helix was solvated in a rectangular box of Tip4P/Ew [7] totaling 122,016 particles. The histone wrapped and **4AM** fragmented ssDNA were solvated in a truncated octahedral box totaling 148,676 and 75,260 particles, respectively. Production MD simulations were performed as described in the section ***Molecular dynamics simulations***.

Modeling of the molecular motor

The tetra-arm-motor (**4AM**) structure was modeled based on the rotor and stator components of the molecular motor crystallized by Li et al. [8] The **2AM** rotor and stator were adapted from **4AM** based on the structure by Klok et al. [9] The motor arm components were generated from their corresponding SMILES strings using the Open Babel program. [10] The DBCO-azide adduct was modeled based on the crystal structure by Mazzoccanti et al. [11] The two motors, arm, and DBCO with and without azide were all parameterized separately as individual residues to allow modular construction of different motors and connection patterns with the DNA. Motor residues include the rotor and stator as well as the branching atoms through the first alkyl-amine. The arm residues include the atoms after the alkyl-amine and before the first alkyl-azide, and the remaining atoms comprise the DBCO-azide component. Each component was parameterized with methyl caps at the attachment points. The systems were built to have motor and DBCO components constructed as one molecule consisting of four separate DBCO-azides, four separate arms, and one motor totaling 9 residues, in addition to the DNA 40-mer. The two molecules were then bound using LEaP [12] during system build.

Attachment of the molecular motor to ssDNA

Due to lack of structural information for the ssDNA-motor conjugate, specifically the attachment patterns between motor arms and the DBCO positions, modeling of the conjugate departed from a motor conformation with arms stretched out. The fragments of ssDNA were placed near the arms according to the desired attachment configuration. Then, through minimization and equilibration simulations, the fragments were pulled together and close to their corresponding motor arm using half harmonic restraints with weights of 5 kcal/mol·Å^2 to bring the distances below 4 Å. The system was then rebuilt as continuous ssDNA with DBCO-dT–azide linkages bound to the motor. This approach was used for each of the three topologically distinct attachment cases considered in this work.

Molecular dynamics simulations

All simulations were performed with AMBER24 [13] utilizing GPU-accelerated MD, [12] with the OL15 [14] DNA force field, Tip4P/Ew [7] water model, and Joung-Cheatham monovalent ion parameters. [15] The molecular motors and DBCO-dT attachments were parameterized using the Antechamber program [16] to assign AM1/BCC charges [17] and GAFF force field [18] parameters. Electrostatics were treated using the particle mesh Ewald (PME) method [19-20] using 12 Å real-space cutoffs and 1 Å^3 regular grid. SHAKE [21] constrains were applied to covalent bonds involving hydrogen.

Hydrogen mass repartitioning was applied to each system to enable longer time steps during simulations. The equilibration process for each system consisted of energy minimization with the unrestrained conjugate gradient method for 500 steps followed by heating the system from 0 K to 300 K over 1.6 ns at constant volume.

Production simulations were performed in the isothermal-isobaric (NPT) ensemble at 300 K and 1 atm using the Berendsen barostat [22] and Langevin thermostat [23] with 5 ps^{-1} collision frequency. For each system, five independent 1 μs production simulations were carried out using a 4 fs time step. The starting structure for the subsequent four trials were taken from frames at 250, 500, 750, and 1000 ns of the first trial.

Calculation of generalized work

Steered molecular dynamics (SMD) was used to calculate generalized work related to the rotation of the motor dihedral angle (Fig. S2b). Simulations were performed in the NPT ensemble at 300 K and 1 atm with a 2 fs time step. Rotations were enforced at constant velocity from 0 to 2π rad over 20 ns (0.1π/ns) with a force constant of 500 kcal/mol·$Å^2$, with work outputted every 20 ps. Five independent trials were performed for each motor attachment variation, the initial structures of which were taken from time stamps 0, 50, 100, 150, and 200 ns of the corresponding production simulations. Sequential SMD simulations were performed until the motor reached a limit due to steric hindrance, and if the limit was not reached, stopped after 10 turns. The generalized work is computed by integrating the force over the course of the simulation. For each rotation case, work was averaged for the common duration of five trials and was fit to a quadratic equation of the form ax^2+bx+c, from which the rate of change in the work over the simulation was calculated by taking the derivative ($2ax+b$).

Umbrella sampling simulations

All simulations were performed with AMBER24 [13] utilizing GPU-accelerated MD, [12] with the OL15 DNA force field, [14] motor components parameterized by GAFF, [18] Tip4P/Ew water model, [7] and Joung-Cheatham monovalent ion parameters. [15] Umbrella sampling simulations of **4AM**, **DNA–4AM**, **2AM**, and **DNA–2AM** were performed using 72 umbrella windows per turn (2π rad). Initial structures of **DNA–4AM** and **DNA–2AM** were taken from SMD simulations, and **4AM** and **2AM** structures were generated sequentially with 0.5 ns per window. An initial 10 ns of sampling was discarded from the analysis as equilibration from which four independent trials of 20 ns simulations were initiated using different random seeds yielding 80 ns of aggregate sampling per window per turn (5760 ns total sampling per turn). Simulations were performed in the NPT ensemble at 300 K and 1 atm with a 2 fs time step using umbrella potentials with 100 kcal mol^{-1} rad^{-1} force constants. Hamiltonian replica exchange was employed to establish equilibrium sampling conditions with exchanges attempted every 40 fs (20 steps). The reaction coordinate was defined by the dihedral angle indicated in Fig. S2b, and its values were collected every 500 fs (250 steps). The FE-ToolKit program [24] was used to calculate free energy profiles using the multistate Bennett acceptance ratio (MBAR) method [25] with 0.15-rad histogram bin spacing. Error bars represent the standard deviation between four independent trials.

Measurements of ssDNA structural properties

The base stacking overlap area [5] was computed to quantify the helical nature of the ssDNA. This was computed for each pair of stacked bases and averaged over the entire strand before and after motor rotation using the Dissecting the Spatial Structure of RNA [26] (DSSR) program. The software uses the atoms of the stacking bases to define two planes, which are projected onto a common, mean plane. The overlapping area is computed from two dimensional that connect the nucleobase atoms [5, 26]. Exocyclic atoms were included when measuring the overlap area.

Section II. Synthetic Procedures

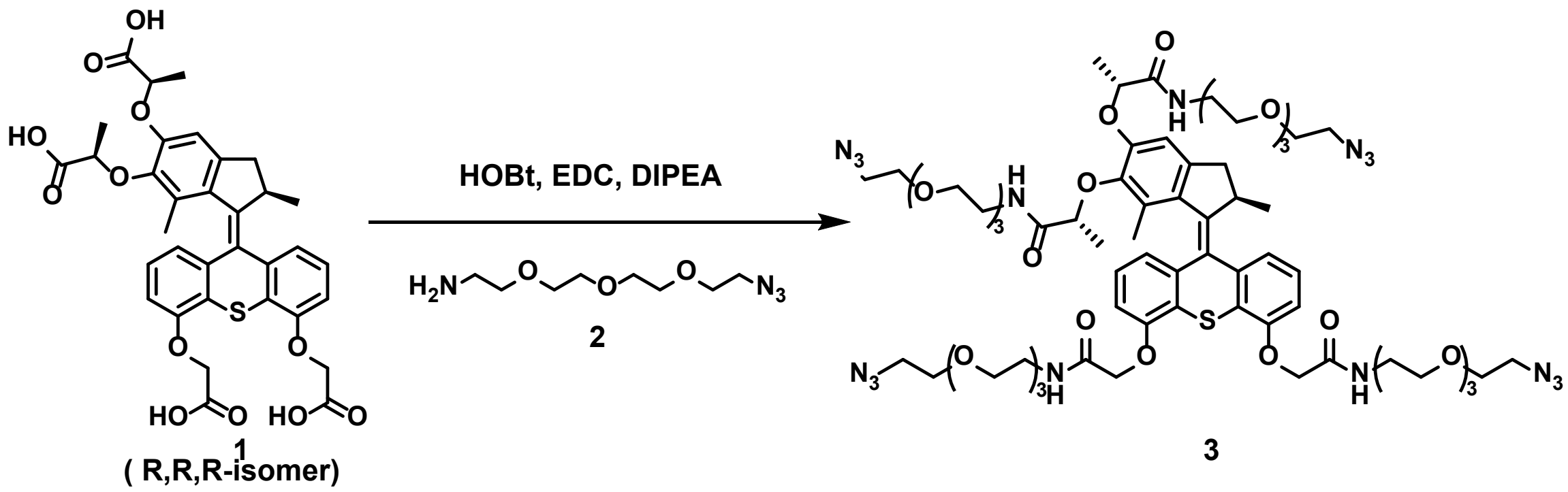

Compound 3 (4AM): The tetra-arm motor **4AM** (compound **3**) was synthesized from compound **1** (enantiopure; prepared via the diastereoselective route reported by Giuseppone et al.) [8, 27] and compound **2** (synthesized according to a previously described procedure) [28]. Briefly, to a solution of compound **1** (30 mg, 45 µmol) in DMF (1 mL) were added hydroxybenzotriazole (HOBt, 73.0 mg, 540 µmol), 1-ethyl-3-(3-dimethylaminopropyl)carbodiimide (EDC, 83.8 mg, 540 µmol), and *N*,*N*-diisopropylethylamine (DIPEA, 46.5 mg, 360 µmol). The mixture was stirred for 10 minutes before the addition of compound **2** (117.9 mg, 540 µmol), and the reaction was allowed to proceed at room temperature overnight. The resulting mixture was diluted with water and extracted four times with dichloromethane (DCM). The combined organic extracts were washed with water and brine, dried over Na_2SO_4, and concentrated under reduced pressure. The crude product was purified by flash column chromatography on silica gel (eluent: DCM/MeOH = 19:1), affording compound **3** (28.8 mg, 43.7% yield) as a purple solid.

^{1}H NMR (500 MHz, $CDCl_3$) δ 7.43 (d, *J* = 7.8 Hz, 1H), 7.32-7.27 (m, 3H), 7.25 – 7.20 (m, 1H), 7.05 (t, *J* = 7.9 Hz, 1H), 6.83 (t, *J* = 5.4 Hz, 1H), 6.80 – 6.71 (m, 3H), 6.68 (s, 1H), 4.72 – 4.60 (m, 4H), 4.57 (d, *J* = 14.8 Hz, 1H), 4.40 (q, *J* = 6.8 Hz, 1H), 4.13 (p, *J* = 6.7 Hz, 1H), 3.69 – 3.49 (m, 57H), 3.39-3.30 (m, 8H), 2.40 (d, *J* = 15.1 Hz, 1H), 1.58 (d, *J* = 6.7 Hz, 3H), 1.33 (d, *J* = 6.8 Hz, 3H), 1.17 (s, 3H), 0.67 (d, *J* = 6.7 Hz, 3H).

^{13}C NMR (126 MHz, $CDCl_3$) δ 172.6, 172.1, 168.2, 168.0, 154.7, 154.3, 150.2, 146.8, 143.4, 143.2, 142.4, 138.8, 133.6, 130.5, 127.6, 127.5, 124.5, 123.9, 121.9, 121.6, 110.2, 109.9, 109.4, 78.0, 76.2, 70.8, 70.8, 70.7, 70.7, 70.7, 70.6, 70.4, 70.4, 70.4, 70.2, 70.2, 70.1, 70.1, 69.9, 69.8, 68.7, 68.6, 50.8, 50.7, 39.6, 39.1, 39.0, 39.0, 38.3, 19.2, 19.1, 18.4, 15.0.

HRMS: *m/z* calculated for $C_{66}H_{97}N_{16}O_{20}S^+$ ($[M+H]^+$): 1465.6786; found: 1465.6946.

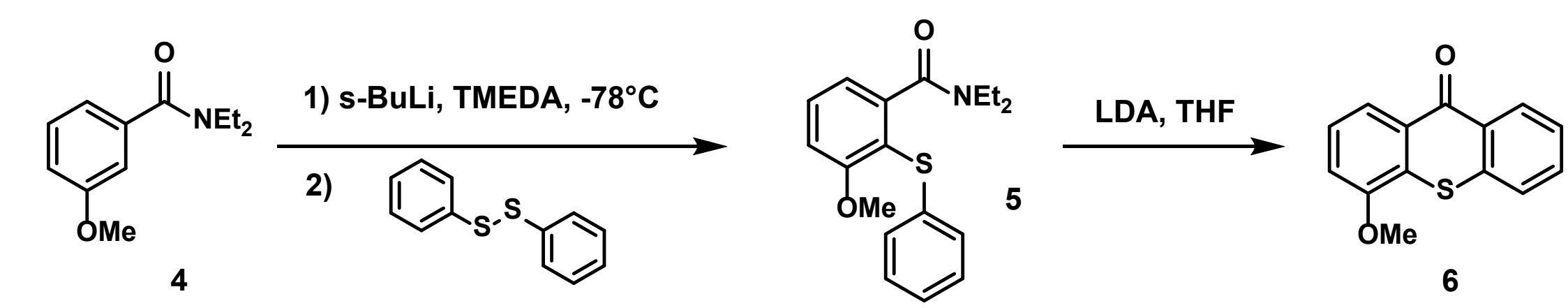

Compound 6: To a solution of THF (15 mL) cooled to –78 °C under nitrogen, 1.43 mL of sec-BuLi (1.4 M in cyclohexane, 2.0 mmol) and tetramethylethylenediamine (TMEDA, 0.32 mL, 2.2 mmol) were added. After stirring for 30 minutes, a solution of compound **4** (414.5 mg, 2.0 mmol, synthesized according to a previously described procedure) [29] in THF (5 mL) was added dropwise, and the mixture was stirred for an additional 30 minutes. Diphenyl disulfide (523.3 mg, 2.4 mmol) was then added as a solid. The reaction mixture was allowed to warm to room temperature and stirred overnight. Water was added to quench the reaction. After removal of THF under reduced pressure, the mixture was extracted with diethyl ether, washed with 1 M HCl, dried over Na_2SO_4, and concentrated under reduced pressure. The residue was purified by flash column chromatography on silica (eluent: DCM/MeOH = 97:3). The obtained crude compound **5** (233.4 mg, 0.74 mmol, 37% yield) was dissolved in 5 mL THF, and the solution was added dropwise to

lithium diisopropylamide (LDA, 3.7 mmol) in 10 mL THF at 0 °C. After addition, the reaction mixture was stirred at room temperature for 1 h. The reaction was then quenched with saturated aqueous NH_4Cl (20 mL) and extracted with diethyl ether (3 × 20 mL). The combined organic layers were dried over anhydrous Na_2SO_4. The residue was purified by flash column chromatography on silica (eluent: hexane/EtOAc = 6:1). The fractions were collected and concentrated to give the product as a yellow solid (104.1 mg, 58% yield).

^{1}H NMR (500 MHz, $CDCl_3$) δ 8.61 (dd, J = 8.4, 1.5 Hz, 1H), 8.26 (dd, J = 8.1, 1.2 Hz, 1H), 7.66 – 7.59 (m, 2H), 7.50 – 7.40 (m, 2H), 7.14 (dd, J = 7.9, 1.1 Hz, 1H), 4.04 (s, 3H).

^{13}C NMR (126 MHz, $CDCl_3$) δ 180.2, 154.5, 137.5, 132.3, 130.5, 129.8, 129.1, 127.7, 126.9, 126.5, 126.1, 121.9, 112.4, 56.6.

HRMS: *m/z* calculated for $C_{14}H_{11}O_2S^+$ ($[M+H]^+$): 243.0480; found: 243.0613.

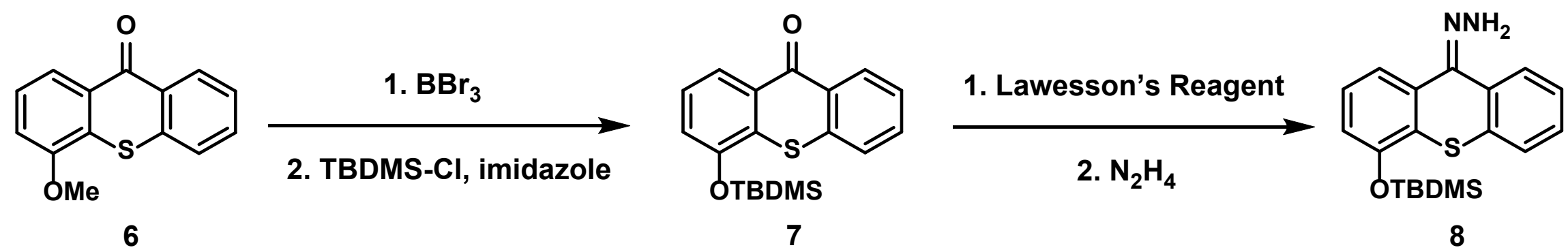

Compound 8: To a solution of compound **6** (1.2 g, 5 mmol) in dry DCM (36 mL), boron tribromide (1 mL, 10 mmol) was added dropwise at 0 °C. The reaction mixture was stirred overnight and allowed to warm to room temperature. Methanol (50 mL) was then added at 0 °C, and the solvent was removed under reduced pressure. This methanol addition and evaporation process was repeated three times. The resulting crude compound was dissolved in dry THF (60 mL), followed by the addition of imidazole (0.5 g, 7.5 mmol) and *tert*-butyldimethylsilyl chloride (TBDMS-Cl, 1.13 g, 7.5 mmol) at 0 °C. The reaction mixture was stirred at room temperature for 3 hours and then filtered. After solvent removal under reduced pressure, the residue was purified by flash column chromatography on silica gel (eluent: hexane/EtOAc = 19:1). The resulting crude compound **7** (0.96 g, 2.8 mmol, 56% yield) and Lawesson's reagent (3.4 g, 8.4 mmol) were dissolved in dry toluene (14 mL) and heated at 80 °C for 2 hours. After cooling, the mixture was filtered through a plug of silica, and the solvent was removed under reduced pressure. The crude residue was dissolved in THF (10 mL), stirred at room temperature, and treated dropwise with hydrazine hydrate (1.5 mL, 28 mmol). The reaction was stirred for 2 hours at room temperature and then concentrated under reduced pressure. The residue was purified by flash column chromatography on silica gel (eluent: hexane/EtOAc = 9:1) to afford compound **8** (794.8 mg, 80% yield), an unstable intermediate used immediately after synthesis without further characterization.

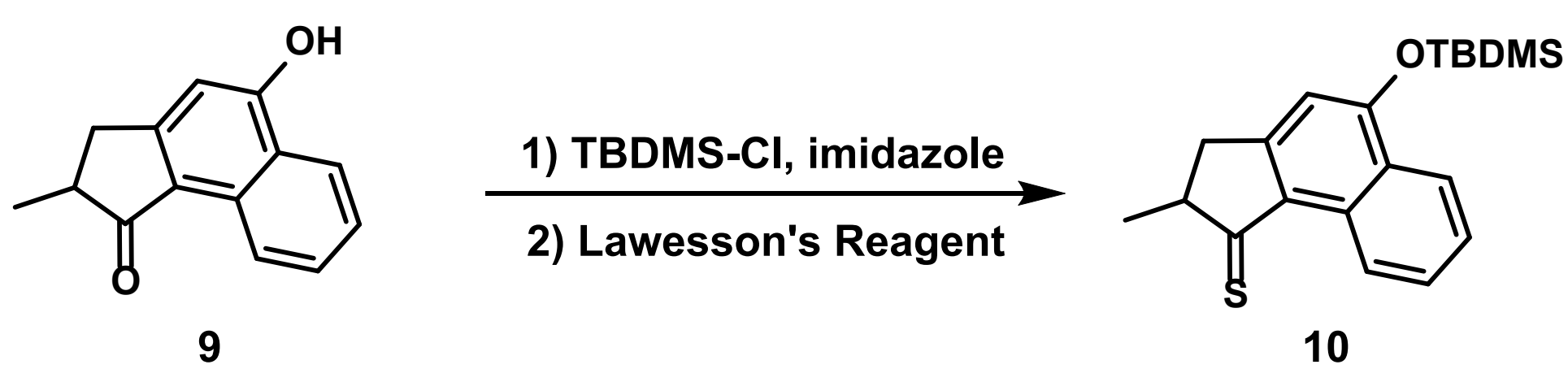

Compound 10: Compound **9** (161 mg, 0.76 mmol, synthesized according to a previously described procedure) [30] was dissolved in dry THF (60 mL), to which imidazole (78 mg, 1.14 mmol) and TBDMS-Cl (172 mg, 1.14 mmol) were added at 0°C. The mixture was stirred at room temperature for 3 hours and then filtered. After evaporation of the solvent, the residue was purified by flash column chromatography on silica (eluent: hexane/EtOAc = 19:1). The crude product (170 mg, 0.52 mmol, 68% yield) was dissolved in dry toluene (6 mL) with Lawesson's reagent (631 mg, 1.56 mmol). The reaction mixture was heated to 80°C for 2 hours. The mixture was then filtered through a plug of silica. The solvent was removed under reduced pressure to afford compound **10** (142 mg, ~80% yield), a highly unstable intermediate used immediately after synthesis without further characterization.

i

Compound 12: Under nitrogen atmosphere and exclusion of light, compound **8** (178.3 mg, 0.52 mmol) was dissolved in dry THF (5 mL), to which activated MnO_2 (755.4 mg, 9 mmol) and Na_2SO_4 (710.2 mg, 5 mmol) were added at 0°C. After stirring at 0°C for 20 minutes, A solution of compound **10** (142 mg, 0.41 mmol) in THF (2.5 mL) was added dropwise to the mixture at 0°C. The mixture was slowly allowed to warm to room temperature and stirred overnight. The solvent was then removed under vacuum and the residue was purified by flash column chromatography on silica (eluent: hexane/EtOAc = 19:1). The obtained crude compound **11** (113.2 mg, 0.17 mmol, 33% yield) was dissolved in toluene (3 mL) with triphenylphosphine (888.6 mg, 3.39 mmol). The solution was stirred at 120°C for overnight. The solvent was then removed under vacuum and the residue was purified by flash column chromatography on silica (eluent: hexane/EtOAc = 9:1). The crude product (85.8 mg, 0.135 mmol, 79% yield) was dissolved in THF (3 mL), TBAF (0.27 mL, 1 M in THF) was added at 0 °C, and the reaction mixture was stirred at this temperature for 5 minutes. The reaction was then quenched with a saturated solution of NH_4Cl (6 mL) and extracted with DCM (3 × 12 mL). The combined organic layers were dried over Na_2SO_4 and concentrated under reduced pressure. The resulting residue was purified by flash column chromatography on silica gel (eluent: hexane/EtOAc = 4:1) to afford compound **12** as a racemic mixture (36.8 mg, 67% yield).

^{1}H NMR (500 MHz, $CDCl_3$) *Note: The equilibrium population consists of cis and trans isomers in an approximately 1:1 ratio.*

Isomer 1: δ 8.07 (d, *J* = 8.0, 3.3 Hz, 1H), 7.78 (d, *J* = 7.8 Hz, 1H), 7.60 (d, *J* = 7.8 Hz, 1H), 7.34 (t, *J* = 7.6 Hz, 1H), 7.25 – 7.15 (m, 2H), 6.94 (t, *J* = 8.2 Hz, 1H), 6.91 (d, *J* = 7.1 Hz, 1H), 6.84 (t, *J* = 7.5 Hz, 1H), 6.81 (s, 1H), 6.67 (t, *J* = 7.8 Hz, 1H), 6.66 (d, *J* = 7.8 Hz, 1H), 4.29 (p, *J*=6.7 Hz, 1H), 3.58 (dd, *J* = 15.5, 6.2 Hz, 1H), 2.54 (d, *J* = 15.6 Hz, 1H), 0.81 (d, *J* = 6.9 Hz, 3H).

Isomer 2: δ 8.05 (d, *J* = 8.0, 3.3 Hz, 1H), 7.62 (d, *J* = 7.3 Hz, 1H), 7.38 (d, *J* = 7.5 Hz, 1H), 7.25 – 7.15 (m, 2H), 7.01 (t, *J* = 7.4 Hz, 1H), 6.84 (t, *J* = 8.2 Hz, 1H), 6.82 (t, *J* = 6.6 Hz, 1H), 6.81 (s, 1H), 6.78 (d, *J* = 7.5 Hz ,1H), 6.59 (t, *J* = 7.5 Hz, 1H), 6.38 (d, *J* = 7.4 Hz, 1H), 4.23 (p, *J* = 6.7 Hz, 1H), 3.58 (dd, *J* = 15.5, 6.2 Hz, 1H), 2.54 (d, *J* = 15.6 Hz, 1H), 0.78 (d, *J* = 6.8 Hz, 3H).

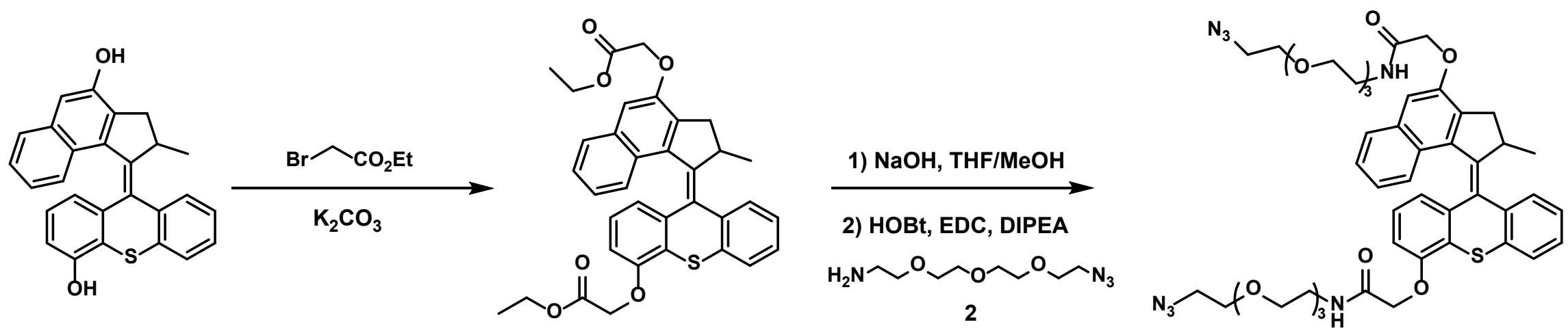

12 13 14

Compound 14 (2AM): Compound **12** (32.1 mg, 78.6 µmol) was dissolved in anhydrous DMF and reacted with ethyl bromoacetate (39.4 mg, 26 µL, 0.236 mmol) and anhydrous K_2CO_3 (32.6 mg, 0.236 mmol). The reaction mixture was stirred overnight at room temperature, then diluted with DCM and washed with water. The aqueous phase was extracted with DCM (2×), and the combined organic layers were washed with brine, dried over Na_2SO_4, and concentrated under reduced pressure. The residue was purified by flash column chromatography on silica gel (eluent: hexane/EtOAc = 4:1). The resulting crude product **13** (19.2 mg, 33 µmol, 42% yield) was dissolved in a 1:1 mixture of THF/MeOH (0.75 mL/0.75 mL), and a NaOH solution (6.6 mg, 165 µmol in 165 µL of deionized water) was added. The mixture was stirred at room temperature for 3 hours and quenched with 1 M HCl at 0 °C. The reaction mixture was extracted with DCM (3 × 15 mL), and the combined organic layers were dried over Na_2SO_4. The crude product was subsequently functionalized with compound **2** via EDC/HOBt coupling, following a protocol analogous to the functionalization of compound **1** to compound **3**, affording the bis-arm motor **2AM** (compound **14**) as a green solid (10.8 mg, 35% yield).

^{1}H NMR (500 MHz, $CDCl_3$) *Note: The compound exists as a mixture of cis and trans isomers, as indicated by the HPLC analysis which displays two peaks in a 1:1 ratio (see Fig. S43), whose ^{1}H NMR signals are difficult to deconvolute. Therefore, the peaks are reported collectively.*

δ 8.09 (d, *J* = 8.4 Hz, 1H), 7.80 (dd, *J* = 7.8, 1.3 Hz, 0.5H), 7.66-7.56 (m, 1H), 7.48 (t, *J* = 7.4 Hz, 1H), 7.44-7.29 (m, 2H), 7.25-7.19 (m, 1H), 7.18-7.08 (m, 1H), 7.02 (td, *J* = 7.5, 1.5 Hz, 0.5H), 6.96-6.73 (m, 4H), 6.71-6.56 (m, 1.5H), 6.44 (dd, *J* = 7.1, 1.6 Hz, 0.5H), 4.74 – 4.51 (m, 4H), 4.27 (dq, *J* = 13.0, 6.7 Hz, 1H), 3.66 – 3.54 (m, 29H), 3.34 – 3.29 (m, 4H), 2.58 (dd, *J* = 15.7, 4.1 Hz, 1H), 0.77 (dd, *J* = 6.9, 4.3 Hz, 3H).

^{13}C NMR (126 MHz, $CDCl_3$) δ 168.1, 154.6, 154.0, 146.7, 146.2, 146.1, 142.1, 140.4, 139.7, 137.9, 135.0, 134.9, 129.8, 129.6, 128.8, 127.9, 127.5, 127.2, 127.0, 126.8, 126.7, 126.5, 126.3, 126.2, 126.0, 125.5, 124.5, 124.0, 122.8, 121.7, 121.3, 110.0, 103.9, 70.7, 70.6, 70.4, 70.0, 69.8, 69.7, 68.6, 68.4, 67.8, 50.6, 40.2, 39.0, 38.9, 37.8, 37.7, 29.7, 19.6

HRMS: *m/z* calculated for $C_{47}H_{57}N_8O_{10}S^+$ ($[M+H]^+$): 925.3918; found: 925.3886.

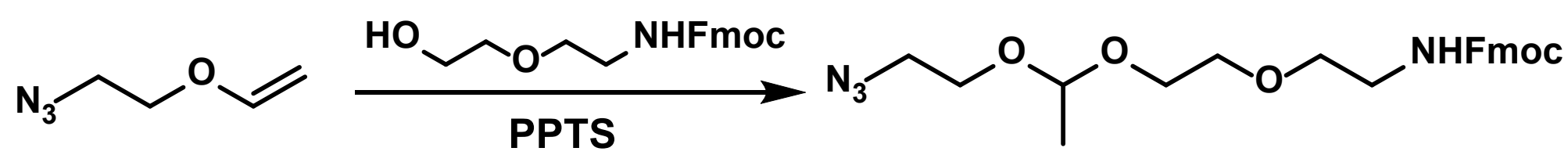

15 **16**

Compound 16: 9H-fluoren-9-ylmethyl *N*-[2-[2-(2-hydroxyethoxy)ethoxy]ethyl]carbamate (500 mg, 1.53 mmol) and pyridinium p-toluenesulfonate (PPTS, 38.5 mg, 0.153 mmol) were dissolved in DCM (38mL). Compound **15** (765.8 mg, 6.77 mmol, synthesized according to a previously described procedure) [31] was added dropwise at 0°C, under N_2. The reaction mixture was stirred at room temperature for 12 hours, diluted with DCM, washed with saturated aqueous $NaHCO_3$, water, and brine, dried over Na_2SO_4, filtered and evaporated. The residue was purified by flash column chromatography on silica (eluent: hexane/EtOAc = 2:1). The fractions were collected and concentrated to give **compound 16** as a yellow liquid (415.5 mg, 62% yield).

^{1}H NMR (500 MHz, $CDCl_3$) δ 7.75 (d, *J* = 7.6 Hz, 2H), 7.59 (d, *J* = 7.5 Hz, 2H), 7.38 (t, *J* = 7.5 Hz, 2H), 7.30 (td, *J* = 7.5, 1.2 Hz, 2H), 5.40 (t, *J* = 5.7 Hz, 1H), 4.79 (q, *J* = 5.4 Hz, 1H), 4.41 (d, *J* = 6.9 Hz, 2H), 4.20 (t, *J* = 6.9 Hz, 1H), 3.78 – 3.67 (m, 2H), 3.67 – 3.49 (m, 6H), 3.46 – 3.23 (m, 4H), 1.32 (d, *J* = 5.4 Hz, 3H).

^{13}C NMR (126 MHz, $CDCl_3$) δ 156.5, 144.0, 141.4, 127.7, 127.1, 125.1, 120.0, 99.7, 70.4, 70.1, 66.6, 63.9, 63.7, 50.9, 47.3, 40.9, 19.4.

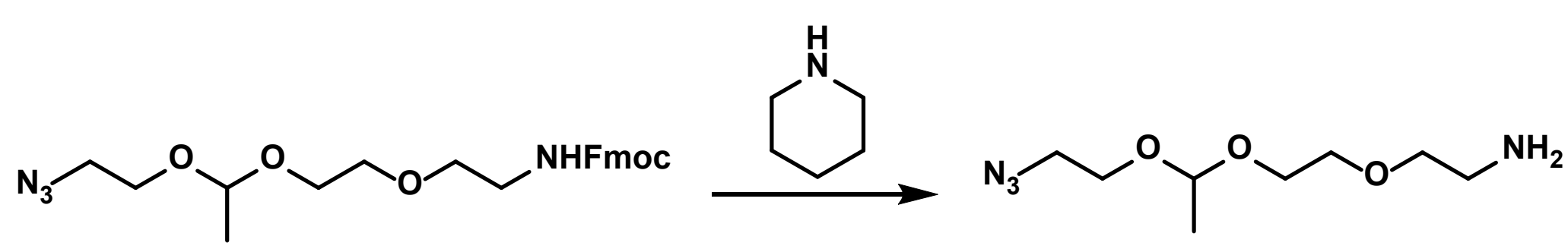

16 **17**

Compound 17: Compound **16** (415.5 mg, 0.94 mmol) was dissolved in DMF (4mL), and 1 mL of piperidine was added. The reaction mixture was stirred at room temperature for 1 hour and then concentrated under reduced pressure. The residue was purified by flash column chromatography on silica (eluent: DCM/MeOH/NEt_3 = 90:10:0.1). The fractions were collected and concentrated to give the product as a yellow liquid (170.2 mg, 83% yield).

^{1}H NMR (500 MHz, $CDCl_3$) δ 4.71 (q, *J* = 5.4 Hz, 1H), 4.00 (s, 2H), 3.70 – 3.60 (m, 2H), 3.56 –3.46 (m, 6H), 3.33 – 3.24 (m, 2H), 2.84 (t, *J* = 5.1 Hz, 2H), 1.24 (d, *J* = 5.4 Hz, 3H).

^{13}C NMR (126 MHz, $CDCl_3$) δ 99.7, 71.3, 70.2, 63.9, 63.8, 50.8, 40.9, 19.3.

HRMS: *m/z* calculated for $C_8H_{19}N_4O_3^+$ ($[M+H]^+$): 219.1457; found: 219.1485.

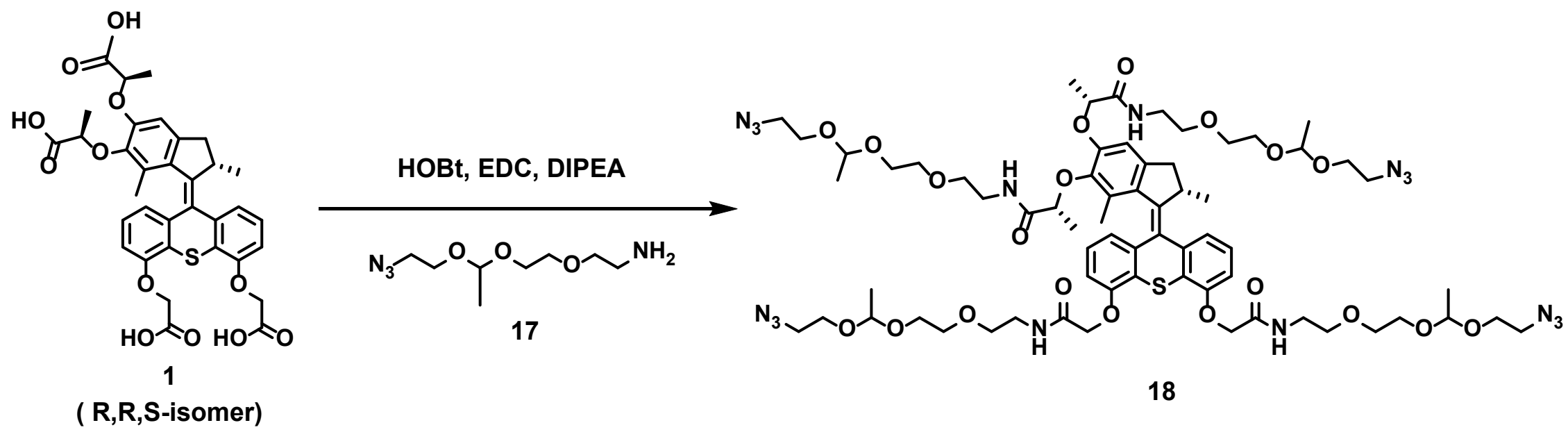

Compound 18 (4AM-X): This compound was synthesized following the same procedure as for compound **3** (**4AM**), except that the R,R,S-isomer of compound **1** was used in place of the R,R,R-isomer, and compound **17** was used instead of compound **2**. The product was obtained as a purple solid (9.3 mg, 25% yield).

^{1}H NMR (500 MHz, $CDCl_3$) δ 7.44 (d, *J* = 7.7 Hz, 1H), 7.42 – 7.36 (m, 1H), 7.31 (t, *J* = 8.0 Hz, 1H), 7.23-7.18 (m, 1H), 7.03 (t, *J* = 6.9 Hz, 1H), 6.93 (t, *J* = 5.3 Hz, 1H), 6.79 (dd, *J* = 7.9, 4.0 Hz, 2H), 6.73 (d, *J* = 8.2 Hz, 1H), 6.69 (s, 1H), 4.90 (t, *J* = 5.6 Hz, 1H), 4.82 (q, *J* = 5.4 Hz, 1H), 4.79 – 4.63 (m, 8H), 4.57 (d, J = 14.8 Hz, 1H), 4.44 (q, J = 7.0 Hz, 1H), 4.14 (p, J = 7.1 Hz, 1H), 3.65 – 3.52 (m, 32H), 3.43-3.36 (m, 8H), 3.35-3.26 (m, 8H), 2.41 (d, *J* = 15.1 Hz, 1H), 1.59 (d, 3H), 1.35 (d, *J* = 5.3 Hz, 3H), 1.31-1.22 (m, 12H), 1.16 (s, 3H), 0.66 (d, *J* = 6.5, 2.8 Hz, 3H).

^{13}C NMR (126 MHz, $CDCl_3$) δ 172.4, 172.0, 168.2, 168.1, 154.9, 154.3, 150.2, 146.8, 143.2, 142.4, 138.8, 133.9, 130.9, 127.6, 124.6, 124.0, 122.2, 121.7, 110.3, 110.1, 109.7, 99.9, 79.2, 76.5, 70.8, 70.5, 70.4, 69.8, 68.8, 64.1, 64.0, 63.8, 51.1, 51.0, 40.4, 39.7, 39.1, 39.0, 38.3, 37.9, 19.6, 19.5, 19.4, 19.4, 19.2, 19.1, 18.4, 15.3.

HRMS: *m/z* calculated for $C_{66}H_{96}N_{16}O_{20}SNa^{+}$ ([M+Na]$^{+}$): 1487.6605; found: 1487.6677.

DNA–4AM, DNA–2AM, and DNA–4AM-X Conjugates: To an aqueous solution of **4DBCO-DNA** (12 nmol, final concentration 10 µM) in a reaction tube were added 2 M TEAA buffer (final concentration 0.3 M) and 3 equivalents of the molecular motor **4AM** (1 mM stock solution in DMSO). The reaction mixture was shaken for 17 hours at room temperature. The **DNA–4AM** was purified by RP-HPLC. The final purity of the products was confirmed by 20% denaturing PAGE.

DNA–2AM, and **DNA–4AM-X** were synthesized analogously. The **DNA–2AM** reaction mixture was first purified by RP-HPLC and then further purified by 20% denaturing PAGE.

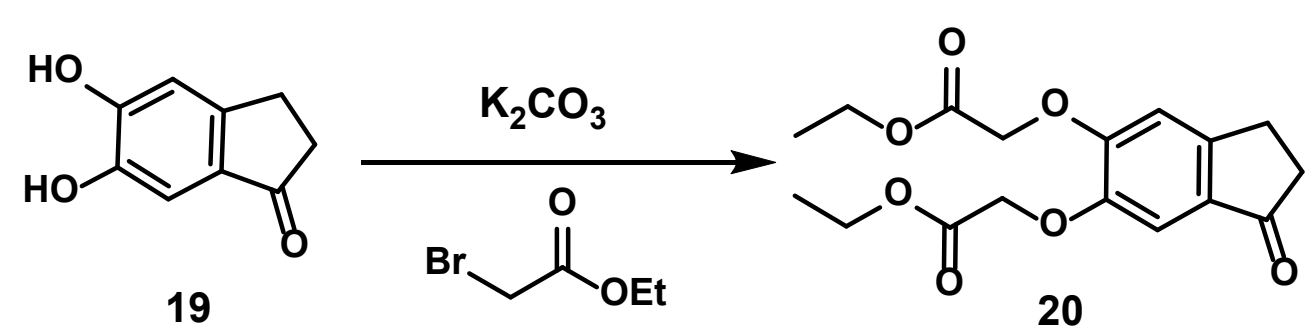

Compound 20: Compound **19** (500 mg, 3.05 mmol, synthesized according to a previously described procedure [32]) and ethyl bromoacetate (1.53 g, 9.14mmol) were dissolved in dry DMF (20 mL), to which K_2CO_3(1.7 g, 12.2 mmol) was added. The reaction mixture was stirred at room temperature for 16 hours, diluted with DCM, washed with water, and brine, dried over Na_2SO_4, filtered and evaporated. The residue was purified by flash column chromatography on silica (eluent: hexane/EtOAc = 2:1). The fractions were collected and concentrated to give compound **20** as a white solid (761.3 mg, 74% yield).

^{1}H NMR (500 MHz, $CDCl_3$) δ 7.14 (s, 1H), 6.84 (s, 1H), 4.79 (s, 2H), 4.72 (s, 2H), 4.27 (dq, *J* = 11.0, 7.1 Hz, 4H), 3.06 – 3.00 (m, 2H), 2.68 – 2.62 (m, 2H), 1.29 (td, *J* = 7.2, 1.4 Hz, 6H).

^{13}C NMR (126 MHz, $CDCl_3$) δ 205.2, 168.1, 153.7, 150.7, 147.9, 130.7, 110.4, 107.1, 66.0, 61.4, 36.4, 25.4, 14.1.

HRMS: *m/z* calculated for $C_{17}H_{21}O_7^{+}$ ([M+H]$^{+}$): 337.1287; found: 337.1292.

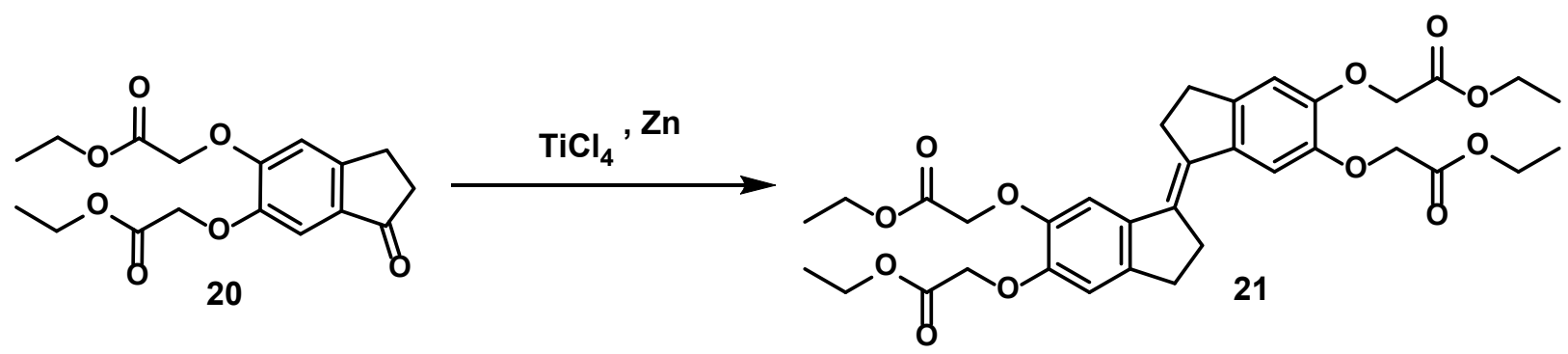

Compound 21: To 409 mg Zn powder (6.26 mmol) under N_2 was added 20 mL THF and cooled to 0 ºC. 344 µL $TiCl_4$ (3.13 mmol) was slowly added and the mixture was then refluxed for 2 hours. The mixture was allowed to cool to 0 ºC, and 0.67 mL pyridine was slowly added, stirred for 10 mins. Compound **20** (500 mg, 1.49 mmol) in 5 mL THF was added to the reaction mixture. The mixture was heated at reflux for 16 h, cooled to room temperature and treated with a saturated aqueous solution of NH_4Cl and extracted with DCM. The organic layer was washed with brine and dried over Na_2SO_4. The residue was purified by flash column chromatography on silica (eluent: hexane/EtOAc = 2:1). The fractions were collected and concentrated to give *trans*-compound **21** as a yellow solid (133 mg, 28% yield).
Note: Zn was activated with 1 M aq. HCl for 15 min, filtered, rinsed with EtOH and Et_2O, vacuum dried under 80 °C for overnight, and stored in a glove box under N_2 atmosphere.

^{1}H NMR (500 MHz, $CDCl_3$) δ 7.19 (s, 2H), 6.81 (s, 2H), 4.73 (d, *J* = 8.1 Hz, 8H), 4.27 (q, *J* = 7.1 Hz, 8H), 3.09 – 2.98 (m, 8H), 1.33 – 1.28 (m, 12H).

^{13}C NMR (126 MHz, $CDCl_3$) δ 169.4, 147.7, 146.8, 141.7, 137.43, 133.4, 112.8, 111.4, 67.7, 66.7, 61.3, 31.9, 30.81, 14.2.

HRMS: *m/z* calculated for $C_{34}H_{41}O_{12}^+$ ([M+H]$^+$): 641.2598; found: 641.2589.

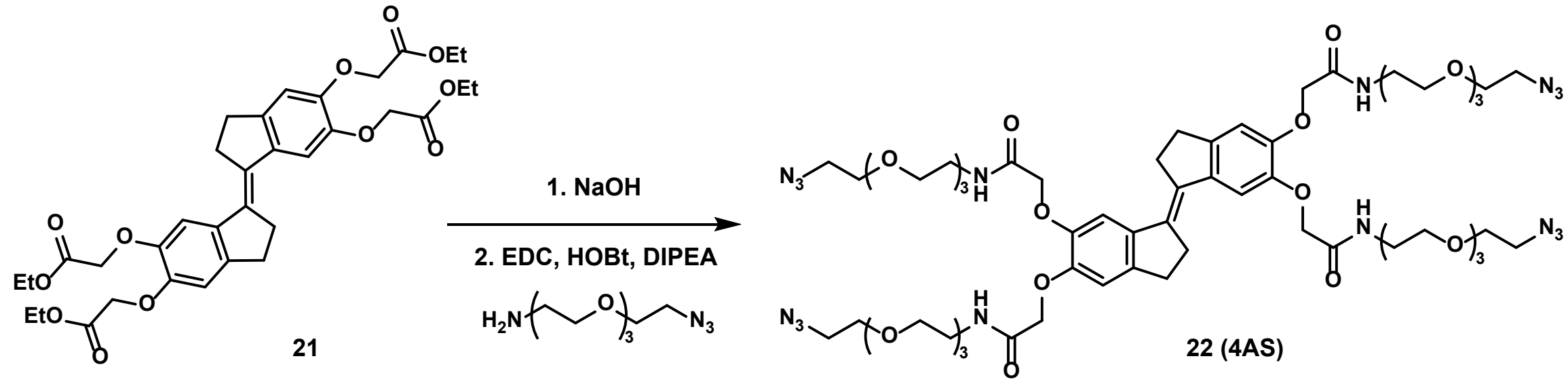

Compound 22 (4AS): This compound was synthesized following the same procedure as for compound **3** (**4AM**) and compound **14** (**2AM**). The product was isolated as the pure *trans* isomer as a yellow solid (29 mg, 12% yield).

^{1}H NMR (500 MHz, $CDCl_3$) δ 7.25-7.10 (m, 6H), 6.87 (s, 2H), 4.58 (d, *J* = 9.2 Hz, 8H), 3.68 – 3.56 (m, 56H), 3.36-3.31 (m, 8H), 3.11-2.95 (m, 8H).

^{13}C NMR (126 MHz, $CDCl_3$) δ 167.5, 146.1, 145.6, 140.9, 136.7, 132.6, 110.5, 69.7, 69.6, 69.3, 69.0, 68.8, 68.7, 68.3, 49.6, 37.9, 30.9, 29.8, 28.7

HRMS: *m/z* calculated for $C_{58}H_{89}N_{16}O_{20}^+$ ([M+H]$^+$):1329.6439; found: 1329.6460.

Section III. DNA Sequence

Table S1. Complete list of all oligonucleotides synthesized in this work.

	**DNA sequence**
**4DBCO-DNA**	5′-XAGTC TTCCC XAGGC AGCAX CGTTC CTGGT XGATA TCTGA-3′ (where X represents DBCO-dT)
**4DBCO-DNA** (without overhanging end)	5′-XAGT CTT CCC XAG GCA GCA TCG TXC CTG GTTGAT ATC XA-3′ (where X represents DBCO-dT)
**2DBCO-DNA**	5′-XAGTC TTCCC TAGGC AGCAT CGTTC CTGGT XGATA TCTGA-3′ (where X represents DBCO-dT)
**0DBCO-DNA**	5′-TAGTC TTCCC TAGGC AGCAT CGTTC CTGGT TGATA TCTGA-3′
Complementary strand (**compDNA**)	5′-GAACGATGCT GCCTA GGGAA-3′

Section IV. MALDI-TOF MS Experiments

After the initial SPAAC reaction between DBCO-containing DNA and **4AM** or **2AM**, subsequent intramolecular SPAAC reactions do not result in additional mass changes. To confirm complete conversion, the isolated oligonucleotides were treated overnight with an excess (~80,000 eq.) of compound **2** (exact mass: 218.1379) and reanalyzed by MALDI-TOF MS. The results showed no further increase in molar mass, confirming that all DBCO groups on the DNA strands had fully reacted with the azides on the molecular motors (Fig. S1).

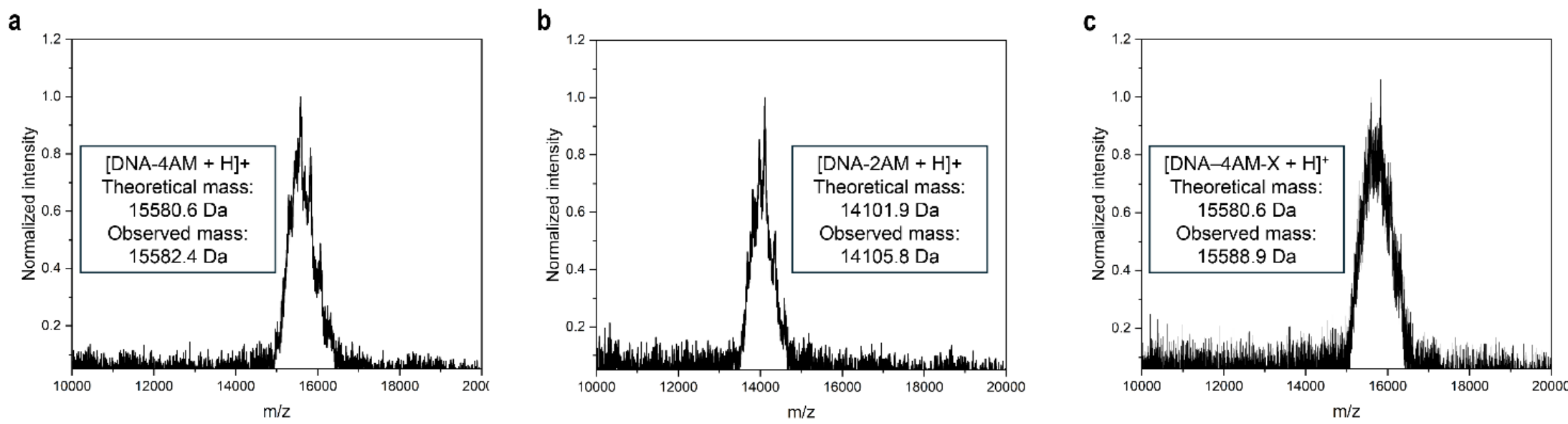

Figure S1. MALDI-MS analysis of **DNA–4AM** (a), **DNA–2AM** (b), and **DNA–4AM-X** (c) after azide treatment.

Section V. Discussion of Topological Isomers in DNA–4AM

The four attachment points (labeled as A, B, C, and D from the 5′ to 3′ end in Fig. S2a) on the **4DBCO-DNA** strand are not equivalent due to their distinct positions along the sequence. The molecular motor is asymmetric relative to its axle, allowing the four attachment points to be grouped into two equivalent pairs: the two on the upper part of the motor (i.e., the rotor, shown in green) and the two on the lower part (i.e., the stator, shown in red) depicted in Fig. S2b.

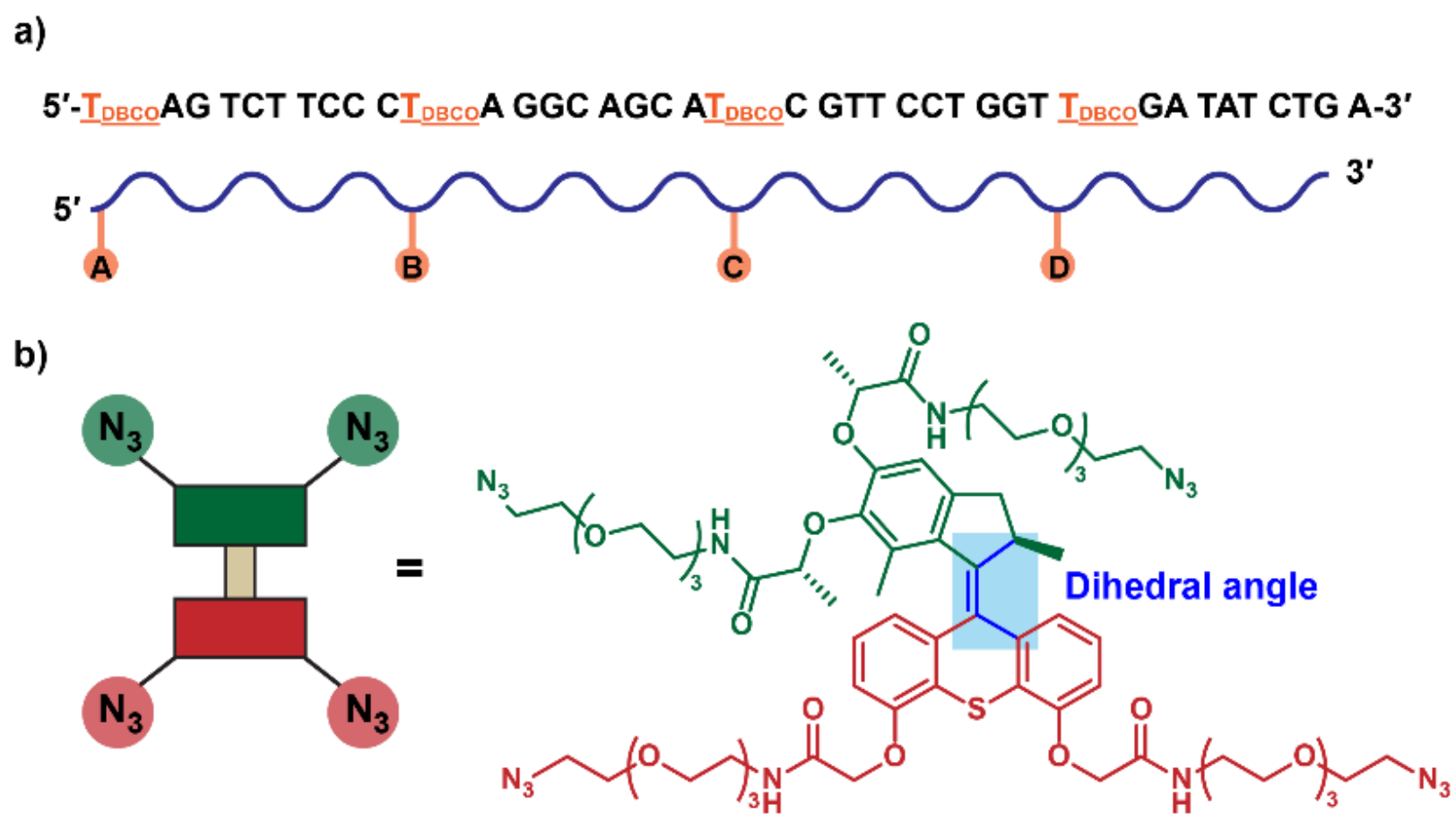

Figure S2. (a) Structure and schematic illustrating the four distinct attachment points on **4DBCO-DNA**. (b) Structure and schematic showing the two asymmetric pairs of attachment points in **4AM**. The dihedral angle subjected to rotation during SMD simulations is highlighted in blue.

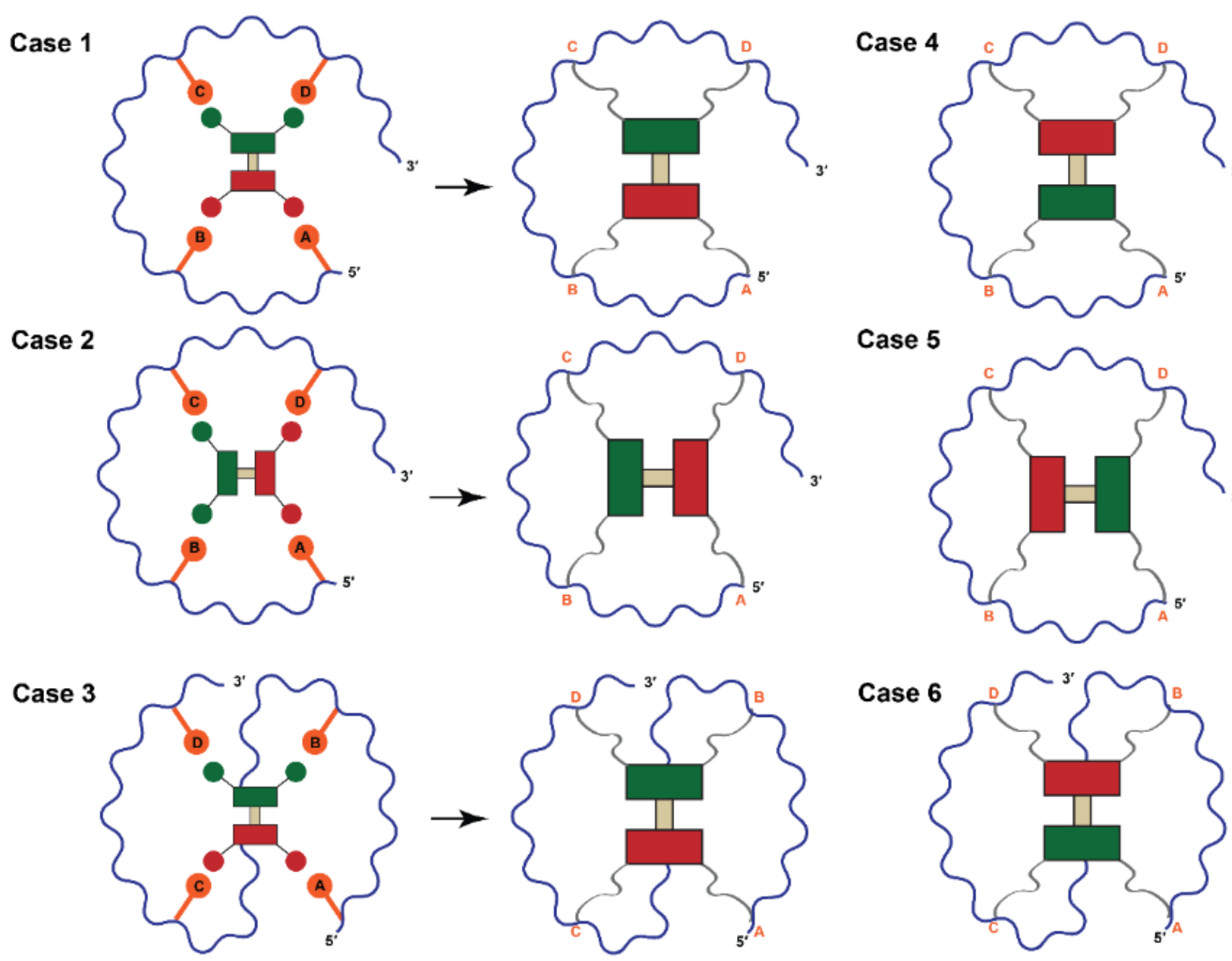

Figure S3. Topological isomers of **DNA–4AM**. From the perspective of motor rotation, Cases 1, 2, and 3 are topologically equivalent to Cases 4, 5, and 6, respectively. Accordingly, only Cases 1, 2, and 3 were explicitly modeled in this study.

As shown in Fig. S3, there are six possible topological configurations for **DNA–4AM**. In Case 1, the two attachment points on the stator bind to points A and B on the DNA. In Case 2, the stator binds to points A and D, and in Case 3, to points A and C. From the perspective of motor rotation, Cases

1, 2, and 3 are topologically equivalent to Cases 4, 5, and 6, respectively. Therefore, the number of distinct isomeric connection patterns effectively reduces to three: Cases 1, 2, and 3.

From a topological perspective, Case 2 and Case 3 adopt the so-called “figure-of-eight” arrangement, in which the rotor and stator are directly connected through two linkages (C to D and A to B). In small-molecule model systems, it has been demonstrated that unidirectional motor rotation within such a “figure-of-eight” topology can generate effective molecular entanglement, thereby mechanically imposing conformational strain on the system.

Case 1 lacks such a “figure-of-eight” topology and may, at first glance, appear to possess a strain-dissipation pathway, rendering it incapable of sustaining mechanical constraints. However, closer examination leads to the opposite conclusion. The only plausible strain-dissipation mechanism in Case 1— one that exploits free rotation around the single bonds along the DNA backbone and spacers to accommodate unidirectional motor rotation—would require threading of the DNA segment from point C to the 3′ terminus through the space enclosed by the segment between C and B and the two tethers connecting C/B to the motor (shaded area in Fig. S4).

While such a threading process might be conceivable in a small-molecule system, it becomes highly improbable in the present macromolecular context. The segment from C to the 3′ end comprises 20 nucleotides (one can visualize its spatial extent from the simulated structure shown in Fig. 4c), and the substantial electrostatic repulsion between negatively charged DNA backbones further disfavors such a threading event. Consequently, the statistical likelihood of this macromolecular threading process is extremely low. In our SMD simulations, we performed more than 50 turns of motor rotation in Case 1 and did not observe a single threading event. Therefore, in the absence of threading, mechanical constraint will inevitably accumulate in Case 1 as well.

In principle, all three topological isomers are capable of sustaining motor-driven mechanical constraints in DNA, whether for topological or steric reasons.

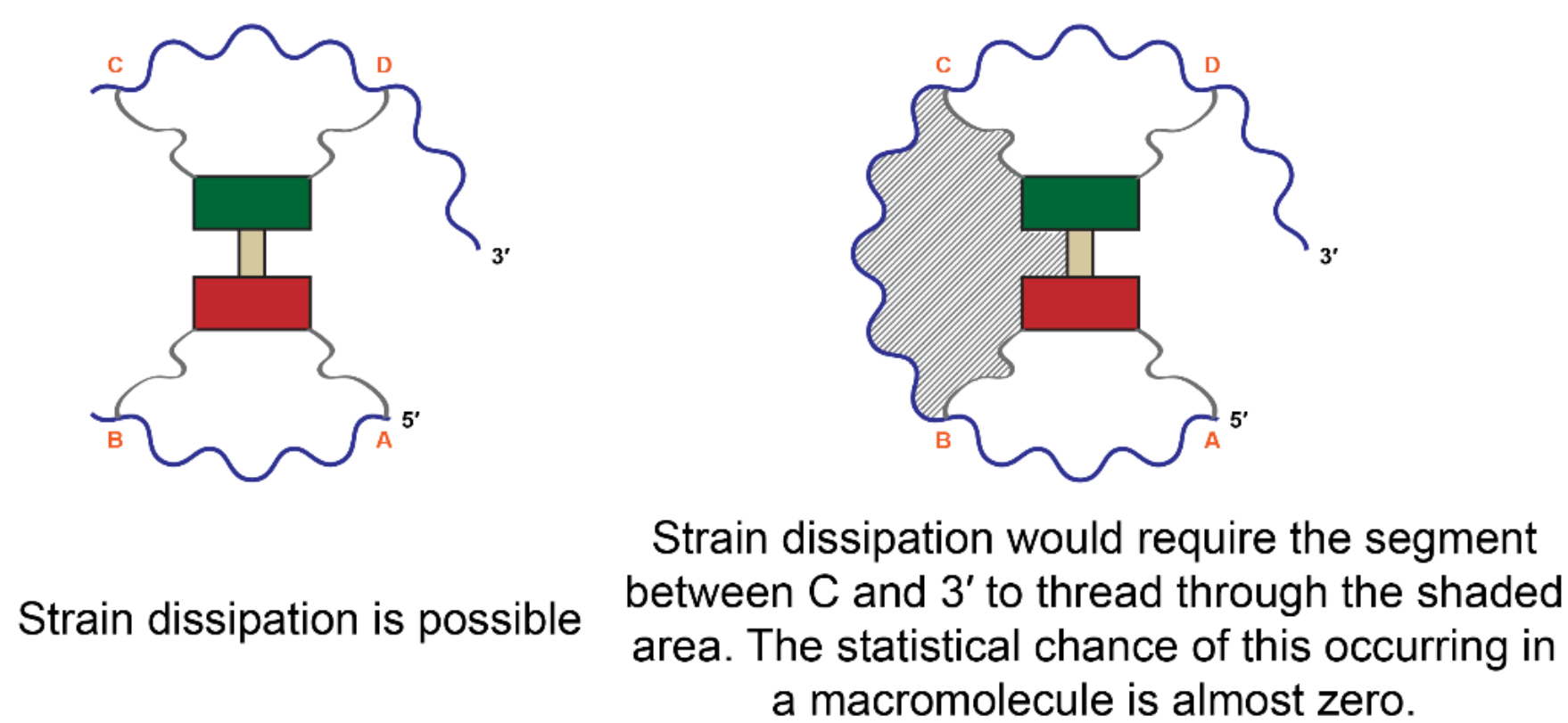

Figure S4. Left: Without the connection between B and C, the system does not allow sustained mechanical constraint. Right: When B and C are connected, strain dissipation would require a highly improbable macromolecular threading event. Thus, Case 1 is also able to sustain mechanical constraint as the motor rotates.

To determine whether the observed mechanical caging of the **DNA–4AM** construct is uniform across all formed topological isomers, we developed an optimized slow-gradient HPLC method to separate the isomers. As shown in Fig. S5, this extended elution profile successfully resolved the original **DNA–4AM** mixture into three distinct peaks. We collected the fractions corresponding to these peaks, isolating three independent samples, each predominantly containing one of the major topological isomers (designated as Isomer 1, Isomer 2, and Isomer 3).

Although the exact topological structure of each isolated fraction cannot be assigned, each is expected to correspond to one of the three topological patterns identified in Fig. S3. Because the purpose of this experiment is to determine whether all three topological patterns are capable of supporting mechanical caging, unambiguous assignment of the individual topological structures is not required.

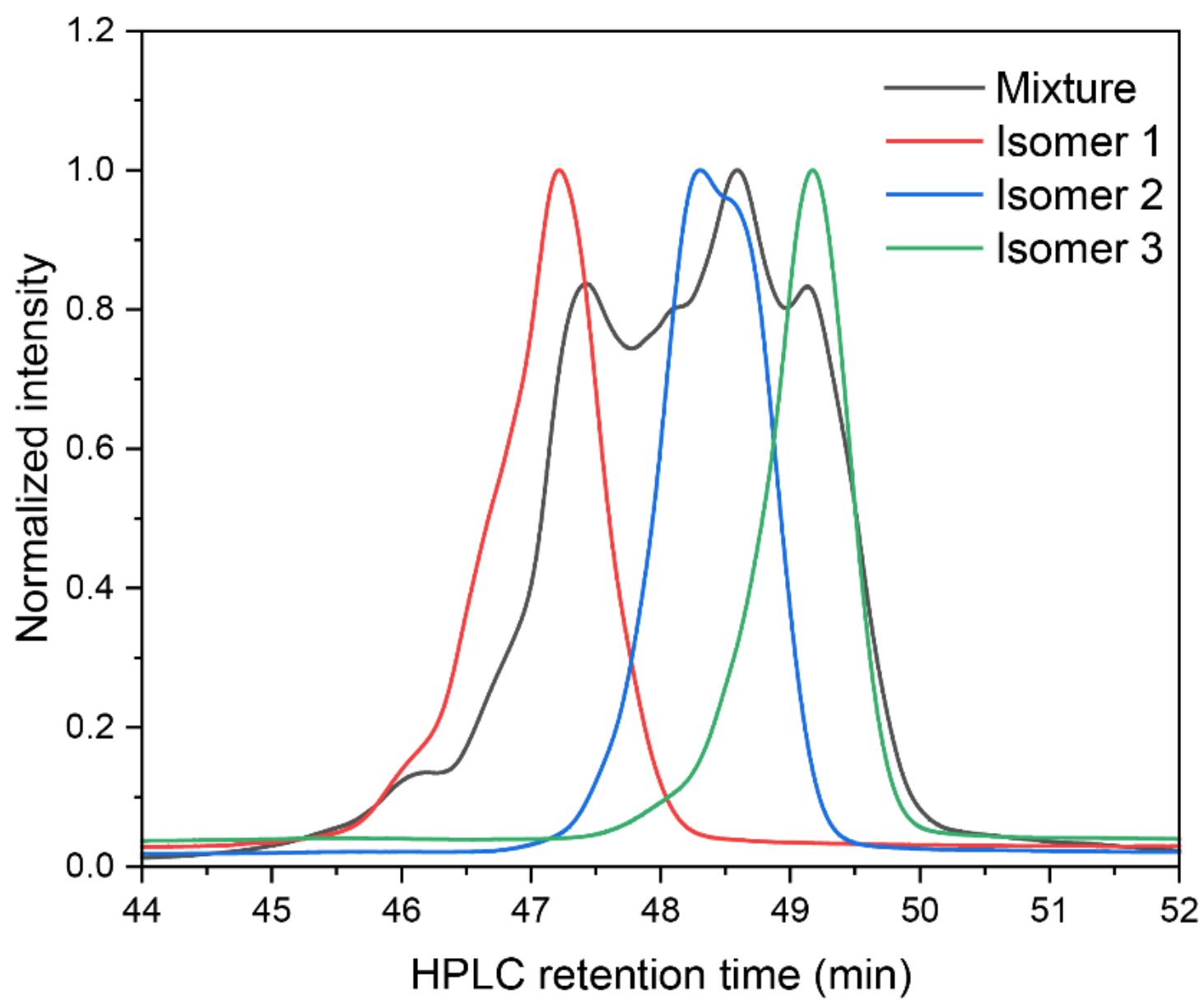

Figure S5. Reversed-phase HPLC analysis (detected at 254 nm) confirming isomer separation. Overlaid chromatograms of the pre-separation mixture (black trace) and the individually purified isomers (red, blue, and green traces) upon re-injection.

We next evaluated the mechanical caging of each isolated isomer using hybridization and exonuclease assays. Native PAGE analysis (Fig. S6a–c) showed highly consistent behavior across all three isolated isomers in the hybridization assay. Before UV irradiation, Isomers 1, 2, and 3 readily hybridized with the complementary DNA (compDNA). Following UV irradiation, however, all three isomers completely lost their ability to form duplexes with compDNA. Likewise, in the exonuclease assay, denaturing PAGE analysis (Fig. S6d–f) demonstrated that each isolated isomer became resistant to nuclease digestion after UV irradiation.

Collectively, these results demonstrate that UV irradiation consistently drives **DNA–4AM** into a compact, mechanically caged state regardless of its topological isomer. The resulting suppression of hybridization and enhanced nuclease resistance are therefore intrinsic properties of the mechanically caged structure, indicating that all three topological isomers identified in Fig. S3 are capable of supporting mechanical caging.

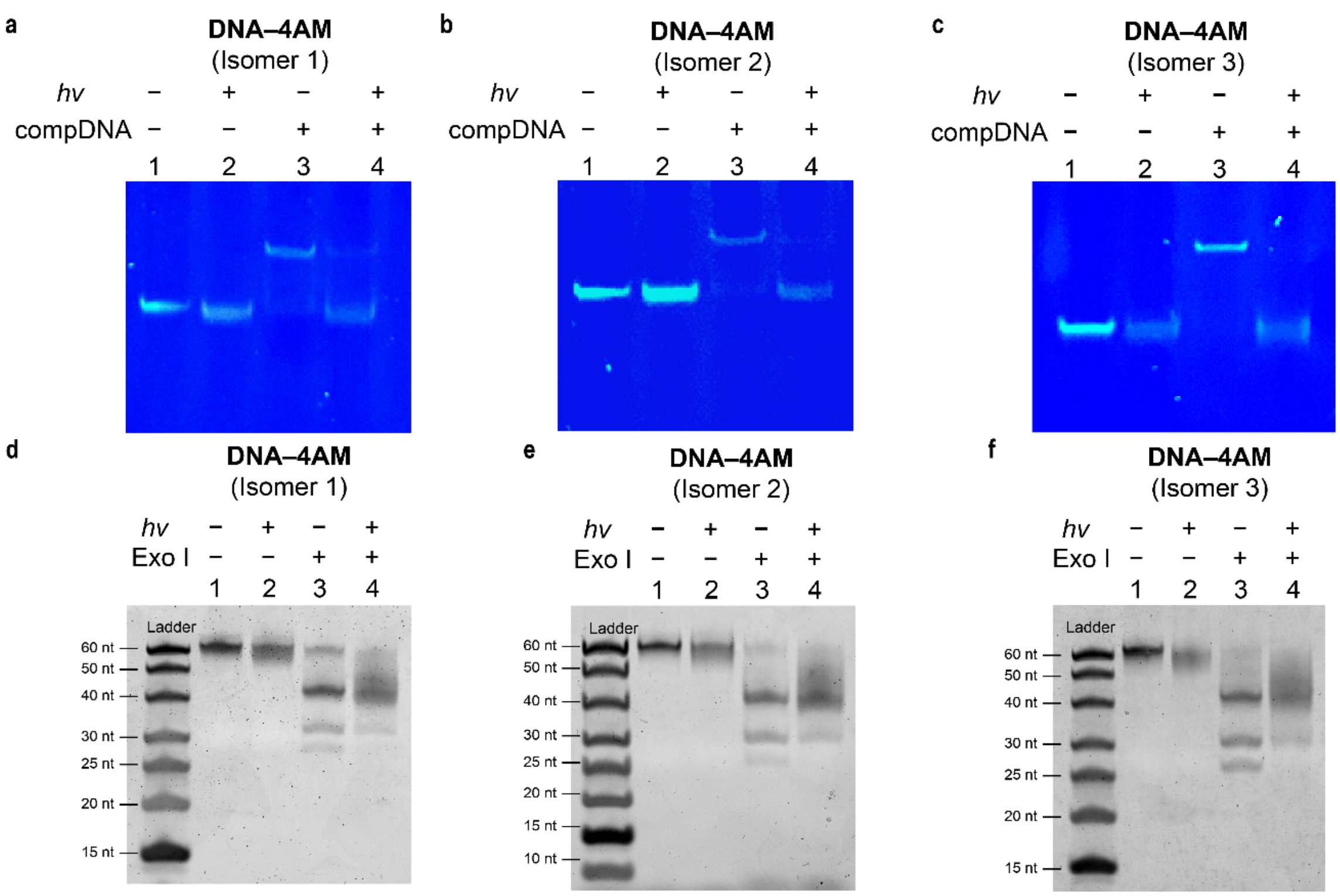

Figure S6. Gel electrophoresis analysis of **DNA–4AM** isomers. (a–c) Native PAGE (20%) analysis showing the hybridization between **DNA–4AM** and compDNA for Isomer 1 (a), Isomer 2 (b), and Isomer 3 (c). Lane 1: nascent **DNA–4AM**; Lane 2: irradiated **DNA–4AM** (365 nm, 2 h); Lane 3: nascent **DNA–4AM** incubated with compDNA in 0.2 M NaCl at room temperature overnight; Lane 4: irradiated **DNA–4AM** incubated with compDNA. Imaged via intrinsic fluorescence of the motor (302 nm excitation). (d–f) Denaturing PAGE (20%) analysis of Exo I digestion for Isomer 1 (d), Isomer 2 (e), and Isomer 3 (f). Lane 1: nascent **DNA–4AM**; Lane 2: irradiated **DNA–4AM** (365 nm, 2 h); Lane 3: nascent **DNA–4AM** treated with Exo I at 37 °C for 2 h; Lane 4: irradiated **DNA–4AM** treated with Exo I. A 10–60 nt ssDNA ladder is shown for size reference. Uncropped images of the original gels are shown in Figs. S58–S63.

Section VI. Estimation of Motor Rotation under the Experimental Irradiation Conditions

The photophysics of second-generation Feringa-type molecular motors closely related to the one employed in this work have been well characterized in previous studies. These reported photophysical parameters allow us to estimate whether the photon dose used in our experiments is, in principle, sufficient to drive motor rotation.

For the fast-rotating second-generation motor used here[9], the photochemical *E*/*Z* isomerization of the central alkene is generally considered the rate-limiting photochemical step under continuous irradiation, whereas the intervening thermal helix inversion occurs on a much shorter timescale than the 2 h irradiation period. Therefore, the overall number of completed rotations is primarily limited by the number of productive photoisomerization events.

The irradiance of the 365 nm UV source used in this work was measured to be I = 3.7 mW cm^{-2}. The molar extinction coefficient of the same motor scaffold at 365 nm has previously been reported as ε = 3891 M^{-1} cm^{-1} [33]. Although the photoisomerization quantum yield (Φ) has not been directly measured for the exact motor used in this study, photophysical investigations of structurally related second-generation Feringa motors have reported values typically between approximately 0.05 and 0.20 [34], with values as low as approximately 0.02 reported for some early motor designs [35]. To provide a conservative estimate, we therefore assume a photoisomerization quantum yield of Φ = 0.02, corresponding to the lower limit ever reported for second-generation Feringa motors.

Based on these photophysical parameters, the incident photon flux (F) generated by the 365 nm UV source can be calculated as

$$F = \frac{I}{E_{\text{photon}}} = \frac{I\lambda}{hc} = \frac{3.7\times10^{-3}\times365\times10^{-9}}{6.626\times10^{-34}\times2.998\times10^{8}} = 6.80\times10^{15}\ \text{cm}^{-2}\ \text{s}^{-1}$$

On the other hand, the molecular absorption cross section (σ) can be obtained from the molar extinction coefficient according to

$$\sigma = \frac{1000\ln(10)}{N_{\text{A}}}\varepsilon = \frac{1000\ln(10)}{6.02\times10^{23}}\times3891 = 1.49\times10^{-17}\text{cm}^{2}$$

Accordingly, the average number of photons absorbed per motor molecule during the 1 h irradiation period can be calculated as

$$N_{\text{abs}} = Ft\sigma = 6.8\times10^{15}\times3600\times1.49\times10^{-17} = 364.8$$

Using a conservative photoisomerization quantum yield of Φ = 0.02, and assuming that each complete 360° rotation requires two productive photoisomerization events, the number of complete rotation cycles that can theoretically be driven per motor during a 1 h irradiation period is estimated to be

$$N_{\text{rot}} = \frac{N_{\text{abs}}\Phi}{2} = \frac{364.8\times0.02}{2} = 3.6$$

This analysis indicates that the irradiation conditions employed in this work are, in principle, sufficient to drive approximately 3.6 complete rotation cycles per motor during a 1 h irradiation period. Because this estimate assumes a deliberately conservative photoisomerization quantum yield, the actual number of rotations is likely higher. It should be noted that this calculation considers only the intrinsic photophysics of the molecular motor and does not account for the mechanical constraints imposed by the attached DNA, which ultimately determine the extent of motor rotation at equilibrium. Nevertheless, these results demonstrate that photon availability is not expected to limit motor rotation beyond a single complete cycle under our experimental conditions.

Section VII. Photostability of DNA–4AM

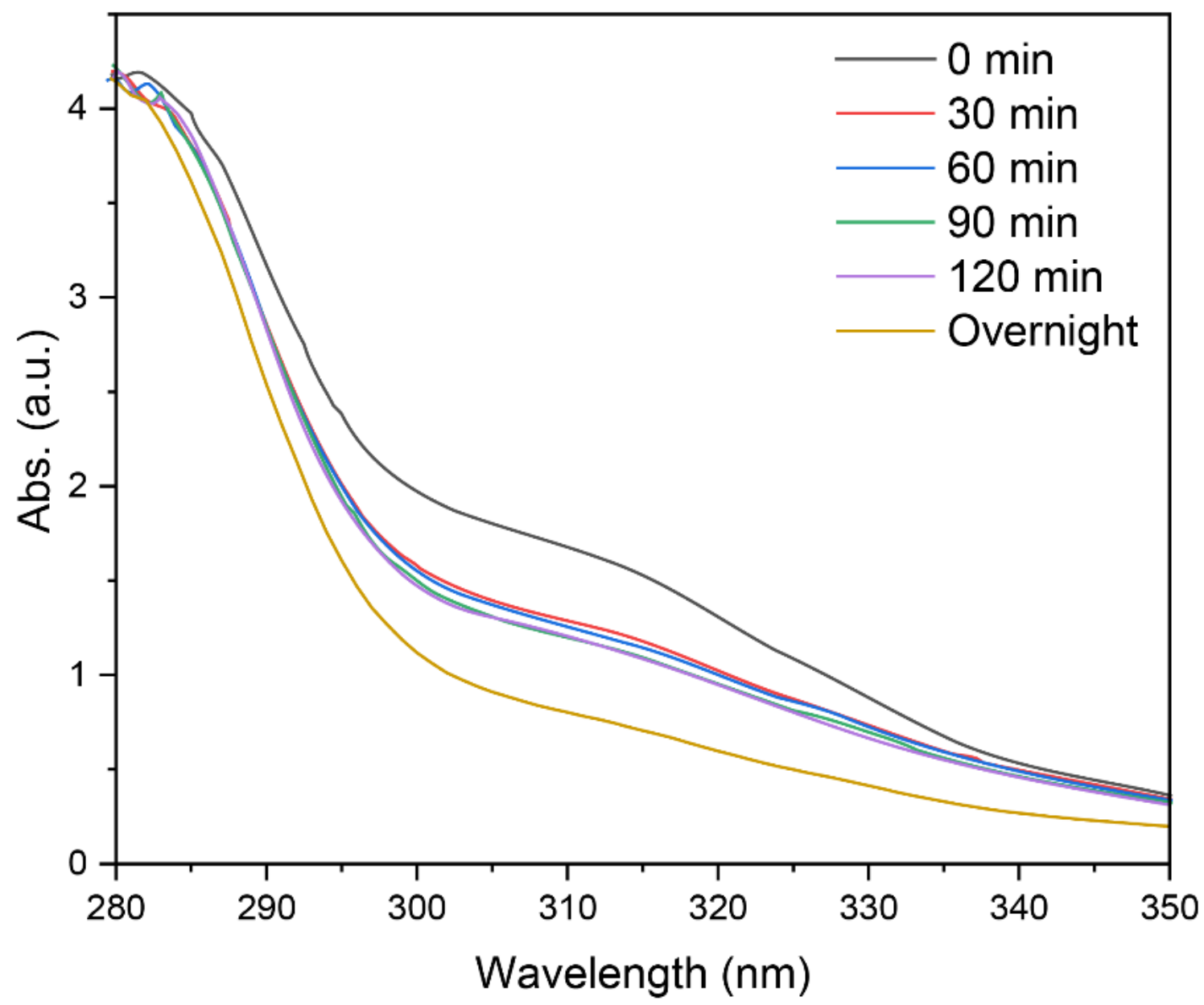

Figure S7. UV–Vis spectra of **DNA–4AM** (H_2O, 25 μM) for different irradiation times.

Our photoirradiation conditions were selected to reflect the intended application of these mechanically caged nucleic acids in aqueous, aerobic environments. To assess the photostability of the molecular motor under these conditions, the stability of the **DNA–4AM** conjugate was monitored by UV–Vis absorption and fluorescence spectroscopy during continuous irradiation. As shown in Fig. S7, the UV–Vis absorption spectrum shifted during the first 30 min, corresponding to establishment of the photostationary state (PSS), and then exhibited only minimal changes over the subsequent 90 min. Likewise, the intrinsic fluorescence of the motor remained unchanged throughout the 120 min irradiation period (Fig. 3a). In both measurements, significant spectral changes were observed only after prolonged overnight irradiation, indicating the onset of photodegradation. These results demonstrate that the second-generation Feringa motor employed in this work remains highly photostable under the irradiation conditions relevant to all experiments. Accordingly, the observed changes in DNA hybridization, nuclease resistance, and conformational behavior originate from mechanically induced caging rather than chemical degradation of the molecular motor.

Section VIII. HPLC and Exo I Analysis of DNA–4AM

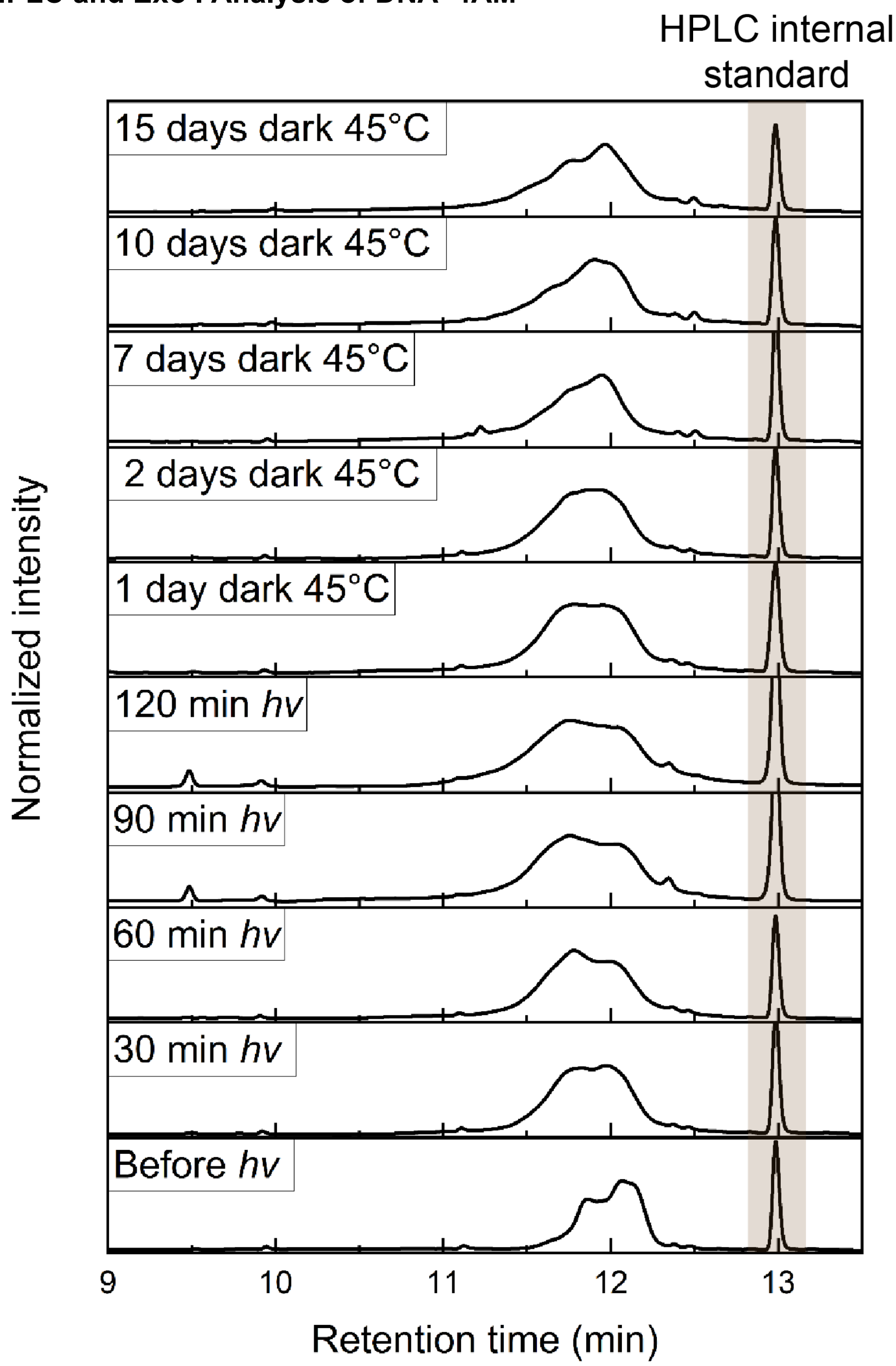

Figure S8. Reversed-phase HPLC analysis (detected at 254 nm) of **DNA–4AM** during 365 nm irradiation and subsequent incubation at 45 °C in the dark.

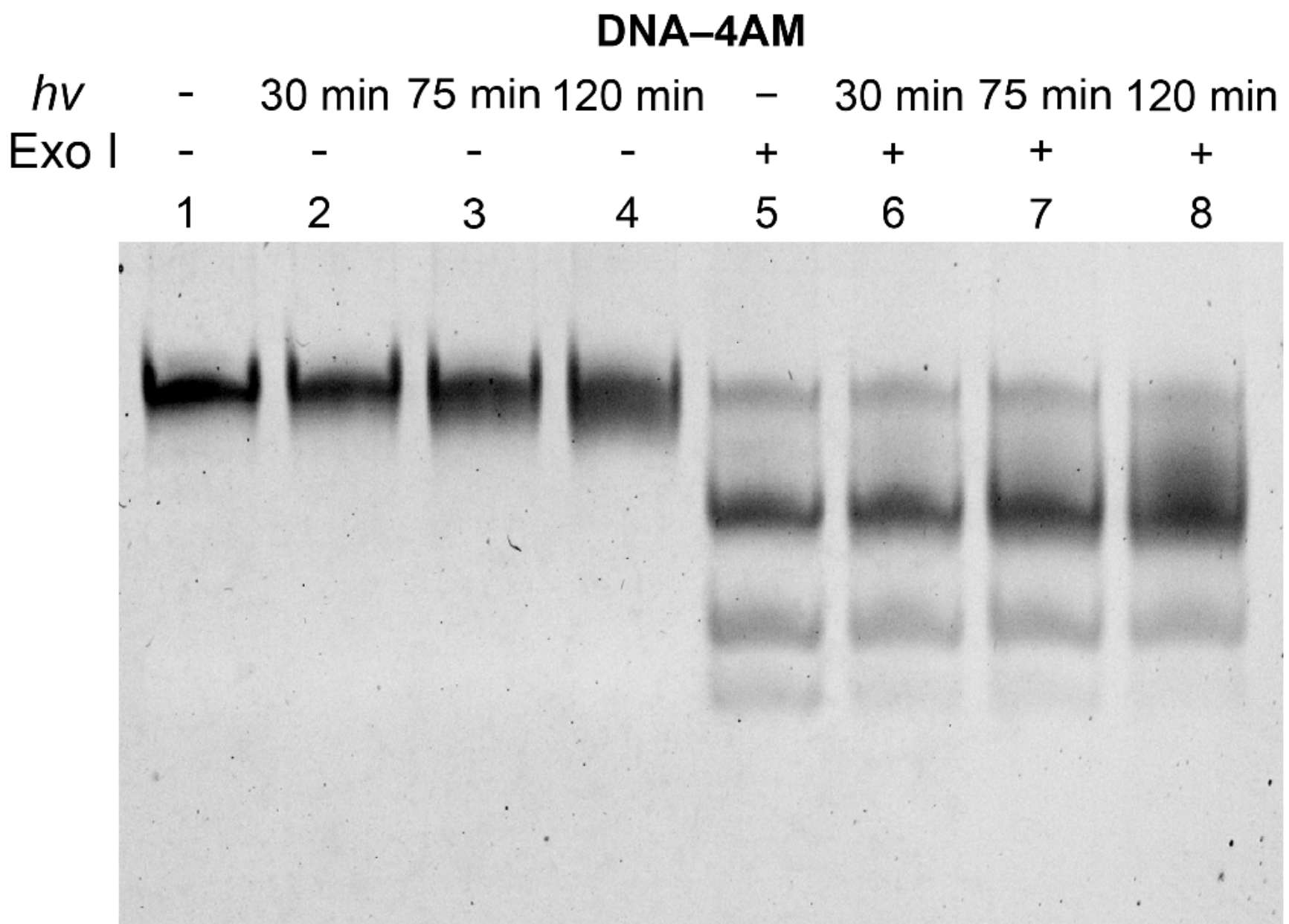

Figure S9. Denaturing PAGE (20%) analysis of Exo I digestion for **DNA–4AM** at different irradiation times. Lane 1: nascent **DNA–4AM**; Lanes 2–4: irradiated **DNA–4AM** (365 nm) for 30 min, 75 min, and 120 min, respectively; Lane 5: nascent **DNA–4AM** treated with Exo I at 37 °C for 2 h; Lanes 6–8: irradiated **DNA–4AM** (30 min, 75 min, and 120 min, respectively) treated with Exo I. An uncropped image of the original gel is shown in Fig. S64.

Due to the presence of multiple structural states of the molecular motor (*cis*-stable, *cis*-unstable, *trans*-stable, and *trans*-unstable), together with the conformational complexity of the **DNA–4AM** construct, directly monitoring motor rotation in our system proved challenging. Nevertheless, by examining the extent of mechanical caging after different durations of UV irradiation, we were able to obtain indirect evidence supporting unidirectional motor rotation beyond a half-turn.

As shown in Fig. S9 (Lanes 1–4), continuous UV irradiation for 30, 75, and 120 min resulted in progressively faster-migrating **DNA–4AM** species on denaturing PAGE, indicative of increasingly compact conformations. This trend is consistent with the HPLC profiles shown in Fig. S8. Furthermore, Exonuclease I digestion confirmed that prolonged irradiation produced progressively stronger mechanical caging. After 30 min of UV irradiation (Lane 6), negligible mechanical caging was observed, with a substantial fraction of the DNA still degraded into shorter fragments. In contrast, extending the irradiation time to 75 min (Lane 7) and 120 min (Lane 8) led to a systematic decrease in the intensity of the lower-molecular-weight degradation products, demonstrating progressively enhanced resistance to enzymatic digestion.

To relate these observations to motor rotation, we further analyzed the UV–Vis spectra of **DNA–4AM** collected after different irradiation times (Fig. S7). The absorbance at 310 nm decreased rapidly during the first 30 min of irradiation, followed by a slower but clearly measurable decline between 30 and 120 min. This behavior closely resembles that reported for the same second-generation molecular motor operating in fully synthetic systems [36]. In that study, the initial rapid decrease in absorbance corresponded to establishment of the PSS associated with photoisomerization (approximately a half-turn of motor rotation), whereas the subsequent slower decrease was attributed to continued unidirectional rotation beyond the half-turn. As the motor accumulated additional rotations, it progressively adopted increasingly strained conformations, resulting in subtle but measurable changes in its intrinsic UV absorption.

Taken together, the UV–Vis, HPLC, denaturing PAGE, and Exonuclease I digestion results support the following mechanistic picture. During the first 30 min of irradiation, the motor completes approximately a half-turn, which produces only limited mechanical caging of the DNA. Continued

irradiation drives further unidirectional rotation beyond the half-turn, resulting in progressively greater DNA compaction and substantially enhanced mechanical caging. Motor rotation eventually ceases as the accumulated conformational strain reaches its maximum. Our MD simulations suggest that this highly strained state corresponds to approximately 1.5 turns of motor rotation. The experimental observations described above are consistent with this prediction, suggesting that the extent of rotation estimated by the simulations is likely close to that achieved in our system.

The accumulated conformational strain in this highly twisted state is responsible for the observed slow thermal back relaxation toward a less strained conformation (Fig. 2c), consistent with previous reports on this class of molecular motors[33, 36]. Collectively, these findings demonstrate that the observed mechanical caging requires a molecular motor capable of sustained unidirectional rotation and cannot be reproduced by a conventional photoswitch that undergoes only reversible half-turn isomerization (see Section XI).

Section IX. SAXS Characterization of DNA–4AM and DNA–2AM

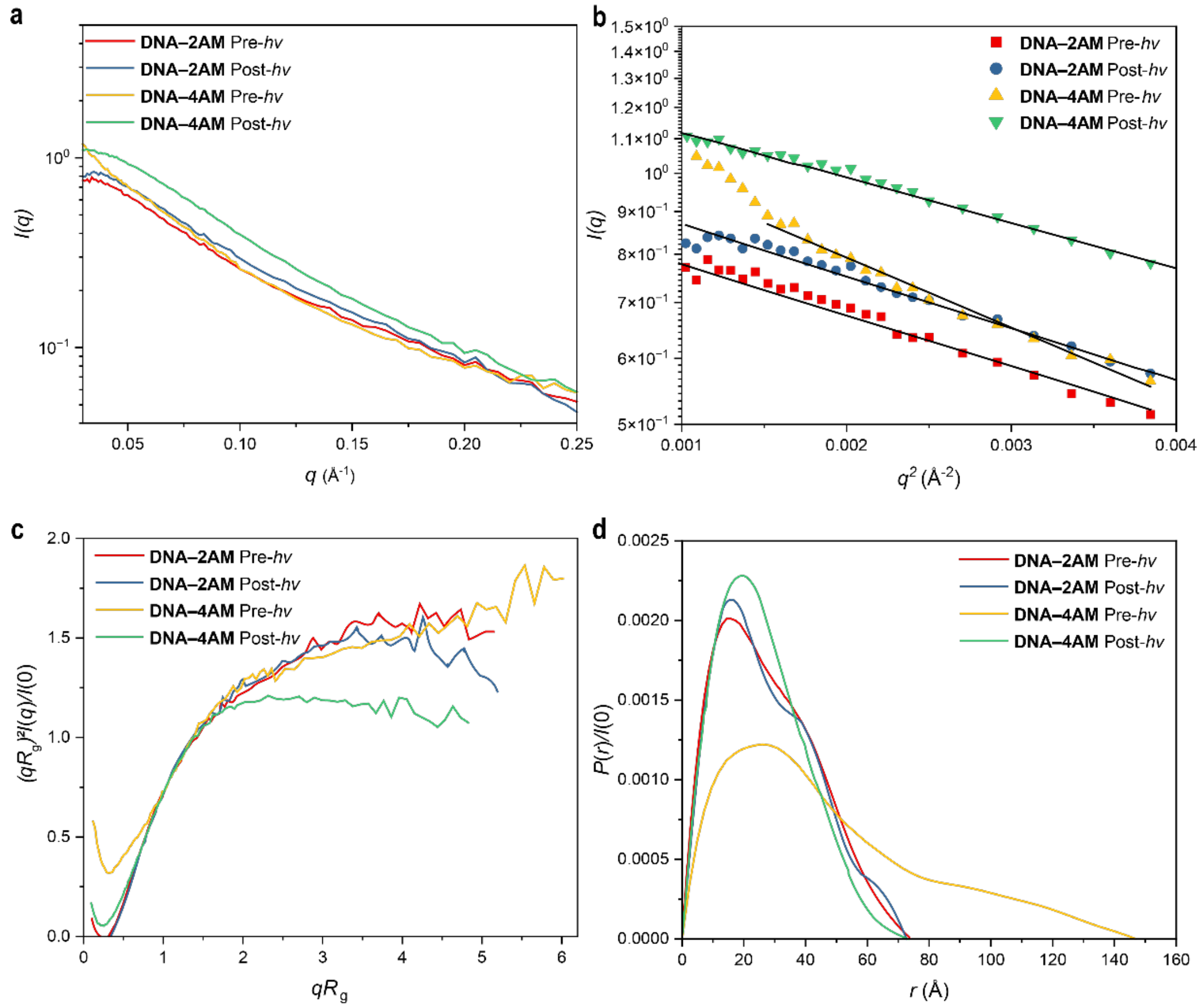

Figure S10. SAXS analysis of nascent and post-irradiated **DNA–2AM** and **DNA–4AM** at 50 µM in water. (a) Scattering profiles. (b) Guinier plots with linear fits shown as black lines (solid symbols represent raw data). (c) Dimensionless Kratky plots. (d) Pair distance distribution functions, $P(r)$, derived from SAXS data. Curves are normalized to $I(0)$.

Table S2. Structural parameters determined from SAXS measurements of **DNA–2AM** and **DNA–4AM** at 50 µM in water by Guinier analysis ("Guinier") and indirect Fourier transform ("GNOM").

	**DNA–2AM** Pre-*hv*	**DNA–2AM** Post-*hv*	**DNA–4AM** Pre-*hv*	**DNA–4AM** Post-*hv*
Guinier R_g (Å)	20.58 +/- 0.52	20.78 +/- 0.45	24.08 +/- 0.3	19.31 +/- 0.55
Guinier $I(0)$	0.9 +/- 0.01	1.0 +/- 0.01	1.17 +/- 0.02	1.27 +/- 0.01
GNOM D_{max} (Å)	101.0	80.0	147.0	90.0
GNOM R_g (Å)	20.48 +/- 1.15	22.24 +/- 0.5	40.43 +/- 0.97	19.56 +/- 0.61
GNOM $I(0)$	0.9 +/- 0.02	1.03 +/- 0.01	1.71 +/- 0.05	1.28 +/- 0.01

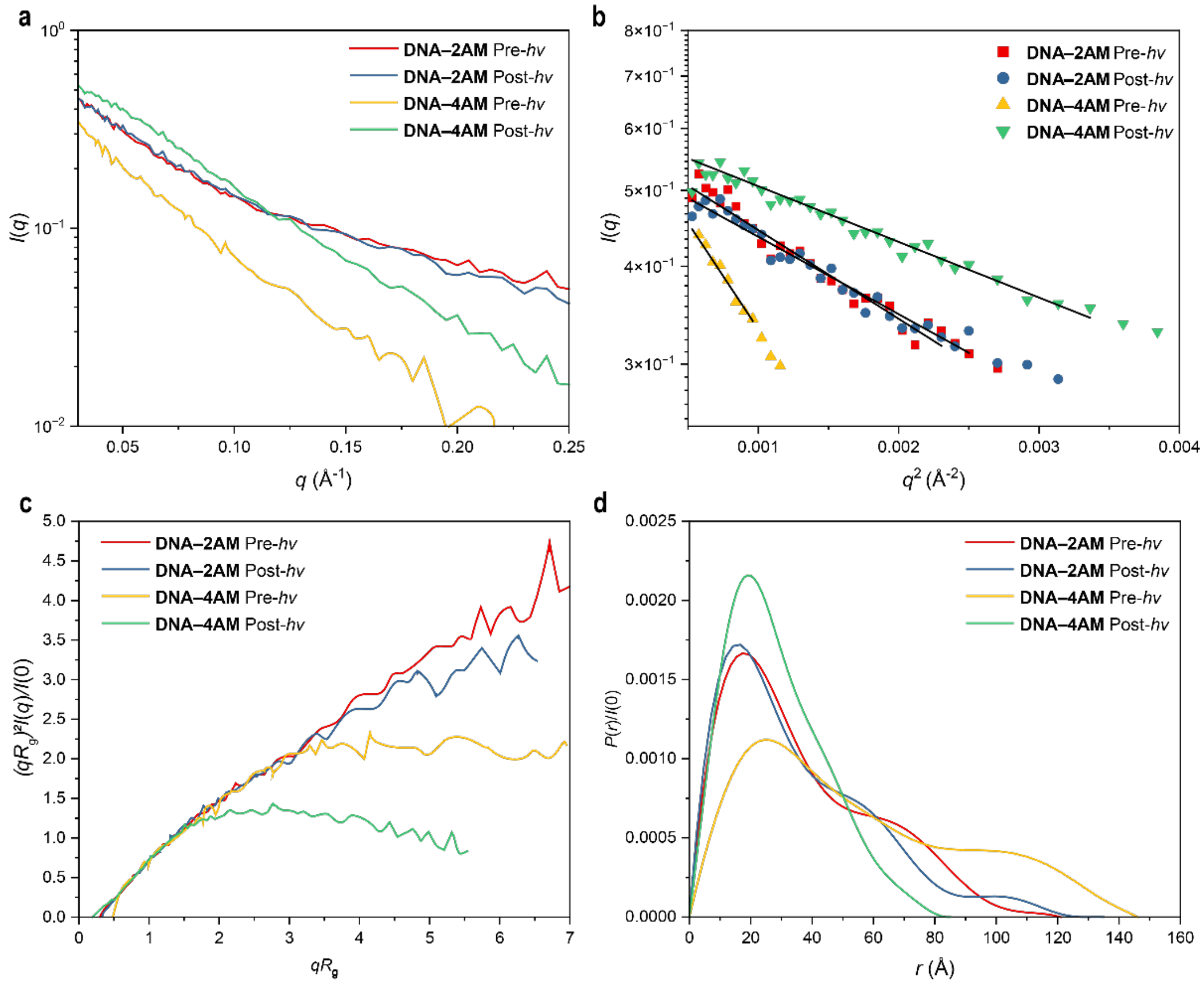

Figure S11. SAXS analysis of nascent and post-irradiated **DNA–2AM** and **DNA–4AM** at 25 μM in water. (a) Scattering profiles. (b) Guinier plots with linear fits shown as black lines (solid symbols represent raw data). (c) Dimensionless Kratky plots. (d) Pair distance distribution functions, $P(r)$, derived from SAXS data. Curves are normalized to $I(0)$.

Table S3. Structural parameters determined from SAXS measurements of **DNA–2AM** and **DNA–4AM** at 25 μM in water by Guinier analysis ("Guinier") and indirect Fourier transform ("GNOM").

	**DNA–2AM** Pre-*hv*	**DNA–2AM** Post-*hv*	**DNA–4AM** Pre-*hv*	**DNA–4AM** Post-*hv*
Guinier R_g (Å)	27.92 +/- 0.45	26.12 +/- 0.5	43.3 +/- 2.45	22.16 +/- 0.26
Guinier $I(0)$	0.58 +/- 6.65e^{-3}	0.55 +/- 7.44e^{-3}	0.62 +/- 0.03	0.6 +/- 4.54e^{-3}
GNOM D_{max} (Å)	121.0	135.0	146.0	85.0
GNOM R_g (Å)	31.09 +/- 0.79	30.59 +/- 2.36	45.44 +/- 1.5	23.17 +/- 0.32
GNOM $I(0)$	0.59 +/- 8.86e^{-3}	0.58 +/- 0.02	0.61 +/- 0.02	0.6 +/- 5.0e^{-3}

Section X. Exo I Assay of the DNA–4AM Construct without Overhanging End

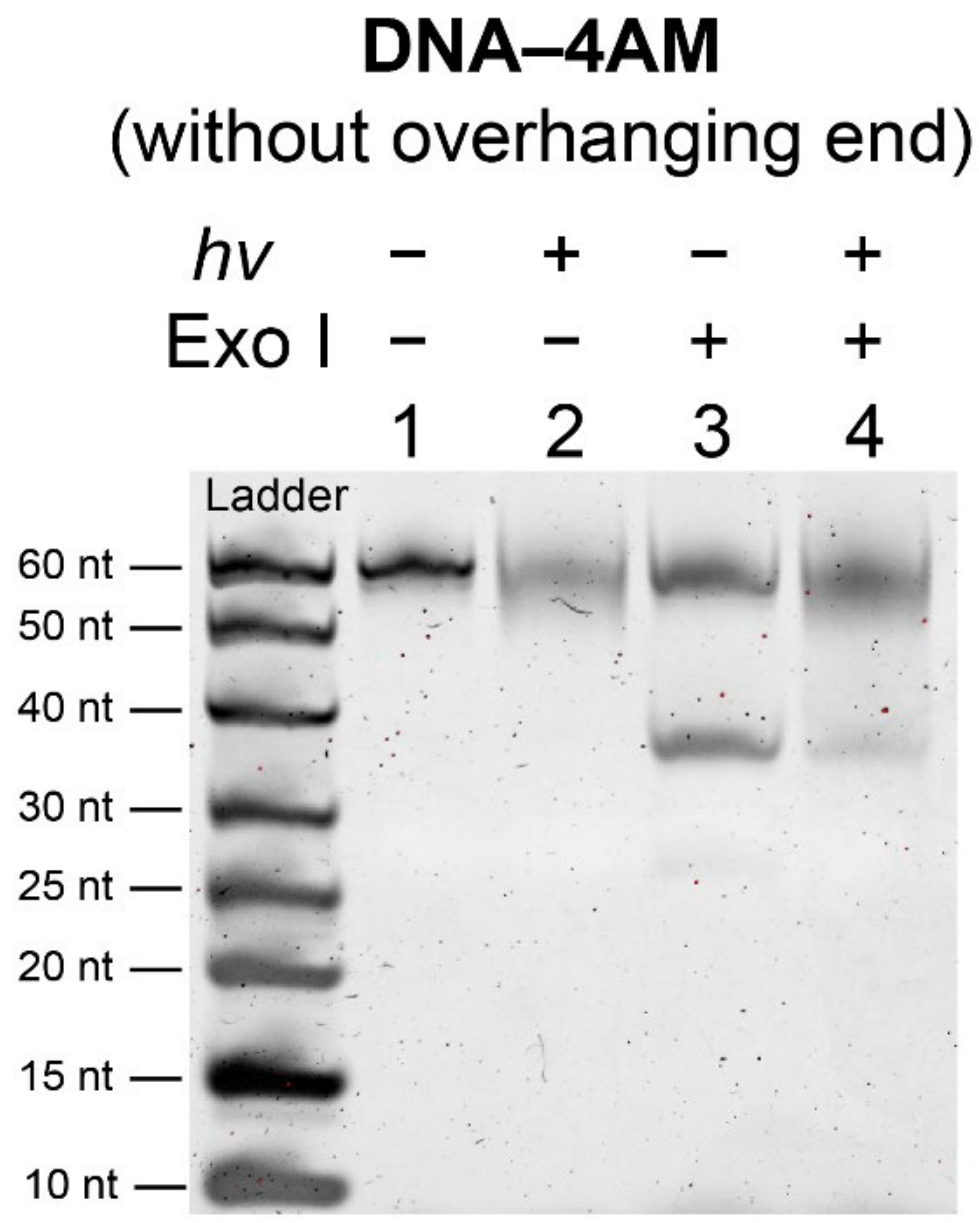

Figure S12. Denaturing PAGE (20%) analysis of Exo I digestion. Lane 1: nascent **DNA–4AM** (without overhanging end); Lane 2: irradiated **DNA–4AM** (without overhanging end) (365 nm, 2 h); Lane 3: nascent **DNA–4AM** (without overhanging end) treated with Exo I at 37 °C for 2 h; Lane 4: irradiated **DNA–4AM** (without overhanging end) treated with Exo I. A 10–60 nt ssDNA ladder is shown for size reference. An uncropped image of the original gel is shown in Fig. S65.

To more clearly visualize the mechanical caging effect, we synthesized a modified **DNA–4AM** construct lacking the 3′ overhang (i.e., by deleting the strand between point A and the 3′ terminus in Fig. 2h). This construct was subjected to the same UV irradiation and Exonuclease I digestion assays. As shown in Fig. S12, before UV irradiation, the unconstrained DNA was readily digested by Exo I, which proceeded past the first DBCO junction adjacent to the 3′ terminus, generating a distinctly shorter DNA fragment (Lane 3). Upon UV irradiation, the DNA adopted a compact conformation (Lane 2) that became almost completely resistant to Exo I digestion, yielding a single predominant intact band (Lane 4). These high-resolution results provide unambiguous evidence that UV irradiation induces a mechanically constrained state that strongly suppresses nuclease digestion.

Section XI. Studies of the DNA–Stiff-Stilbene Conjugate (DNA–4AS)

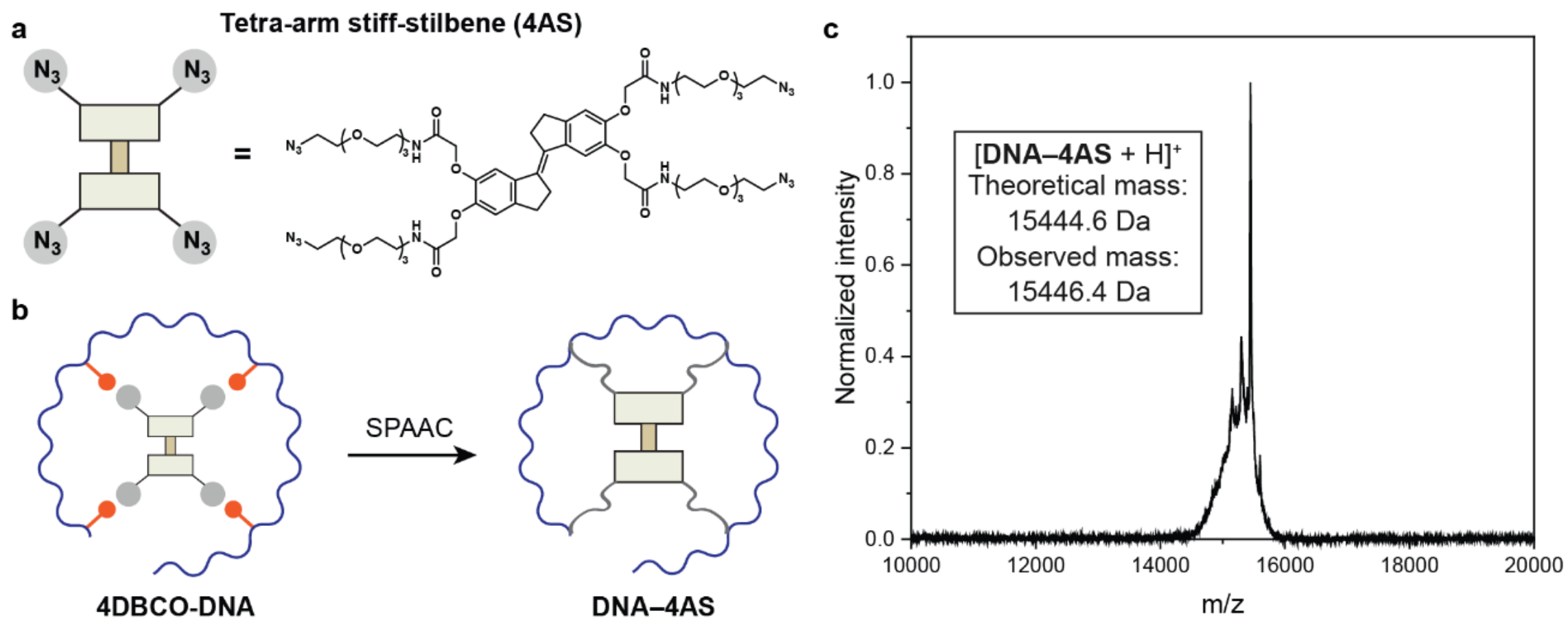

Figure S13. a) Chemical structure of the tetra-arm stiff-stilbene motor **4AS**, synthesized as a control molecule that undergoes only a half-turn rotation upon light irradiation. b) Conjugation of **4AS** with **4DBCO-DNA** to afford **DNA–4AS**. c) MALDI–MS characterization of **DNA–4AS**.

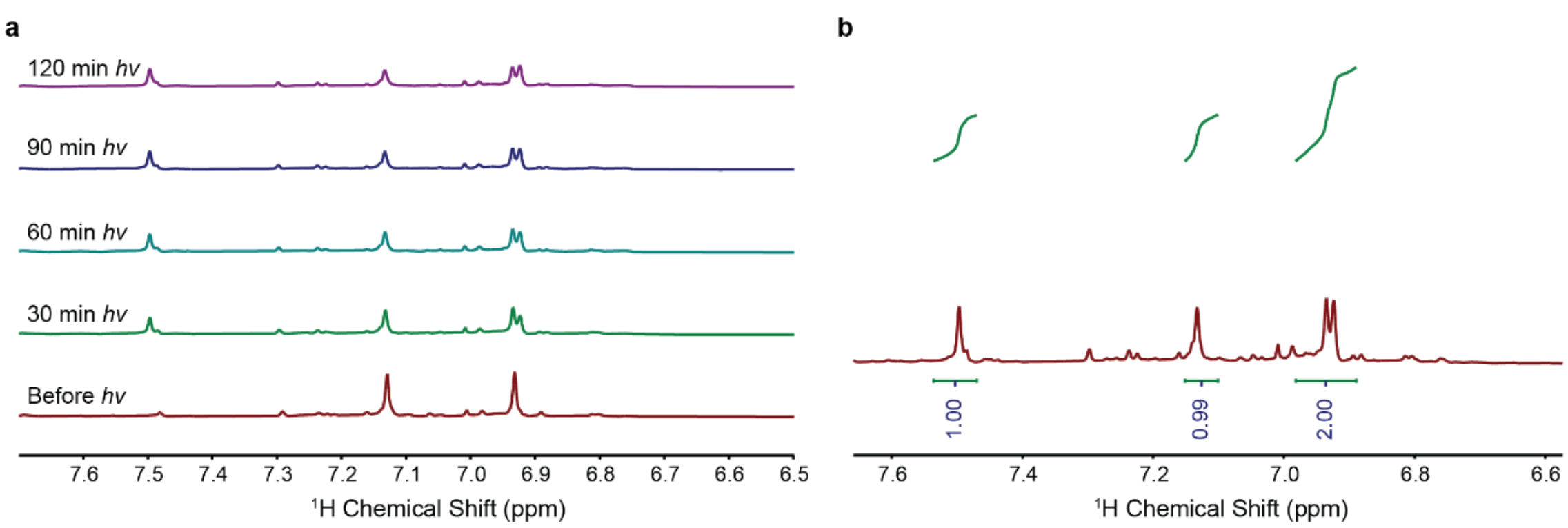

Figure S14. ^{1}H NMR analysis of the photoisomerization of **4AS** under UV irradiation. a) Time-resolved ^{1}H NMR spectra (16% D_2O/84% CD_3CN, 8 mM) recorded at the indicated irradiation times (0–120 min). b) Integrated ^{1}H NMR spectrum after 60 min of UV irradiation, corresponding to the PSS, as evidenced by the plateau in the intensity of the *cis* isomer resonance at 7.50 ppm. Peak integration indicates a *cis*:*trans* ratio of 50:50 at the PSS.

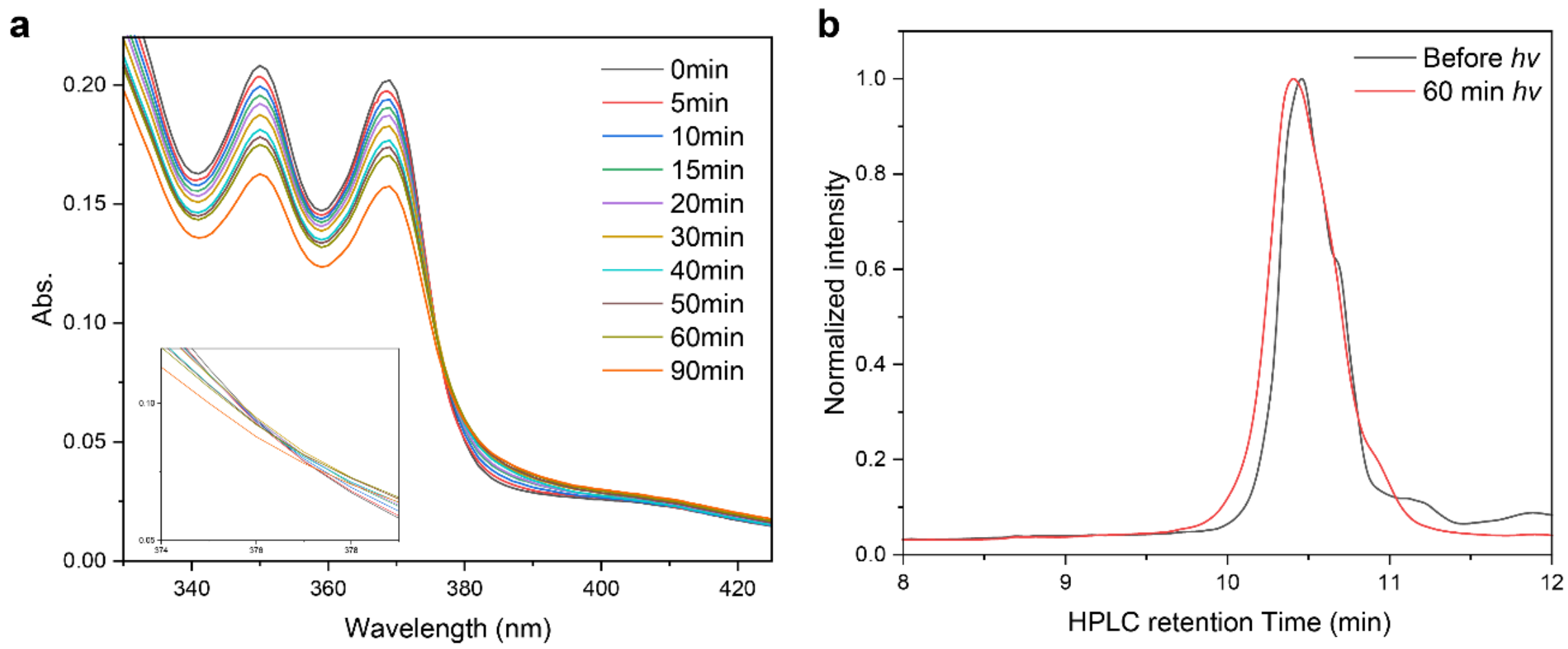

Figure S15. a) UV–Vis spectra of **DNA–4AS** (H_2O, 6 μM) for different irradiation times. b) Reversed-phase HPLC analysis (detected at 254 nm) of **DNA–4AS** before and after 365 nm irradiation.

To further determine whether the observed mechanical caging arises from the unidirectional rotation of the molecular motor rather than from its functioning merely as a photoswitch (i.e., a single half-turn rotation), we designed and synthesized a control system based on a tetra-arm stiff-stilbene (**4AS**) molecule (Fig. S13a), which was isolated in the pure *trans* configuration. Unlike the Feringa motor, stiff stilbene undergoes only nondirectional *trans*–*cis* photoisomerization under UV irradiation and therefore serves as an ideal control for evaluating the effect of a photoswitch capable of only a half-turn conformational change. Conjugation of **4AS** with **4DBCO-DNA** afforded **DNA–4AS** (Fig. S13b), whose structure was unambiguously confirmed by MALDI-TOF mass spectrometry (Fig. S13c).

Time-resolved ^{1}H NMR spectroscopy of **4AS** showed gradual *trans*-to-*cis* photoisomerization upon UV irradiation, reaching the PSS after approximately 60 min (Fig. S14a). Integration of the NMR signals indicated a *cis* ratio of approximately 50:50 at the PSS (Fig. S14b). UV–Vis spectroscopy of **DNA–4AS** similarly revealed a gradual decrease in the absorption band at 370 nm (corresponding to the *trans* isomer), accompanied by an increase in the band at 376 nm (characteristic of the *cis* isomer) consistent with *trans*-to-*cis* photoisomerization. A magnified view of the spectra (inset, Fig. S15a) shows that the system reached the PSS after approximately 60 min of irradiation. Prior to reaching the PSS, the spectral evolution exhibited a well-defined isosbestic point, consistent with a clean unimolecular *trans*–*cis* photoisomerization involving only two absorbing species. Beyond 60 min, the isosbestic point gradually shifted, indicating the onset of photodegradation.

Based on the combined NMR analysis of **4AS** and UV–Vis characterization of **DNA–4AS**, we selected 60 min as the irradiation time for all subsequent experiments. Under these conditions, the stiff-stilbene photoswitch reaches the PSS while photodegradation remains negligible. Because the PSS consists of an approximately 1:1 mixture of *cis* and *trans* isomers, at least half of the **DNA–4AS** molecules have undergone a half-turn photoisomerization. We therefore sought to determine whether this half-turn rotation alone is sufficient to induce the same degree of mechanical caging observed for **DNA–4AM**. If **DNA–4AS** fails to produce comparable mechanical caging despite extensive *trans*–*cis* isomerization, it would provide strong evidence that the mechanical caging observed in **DNA–4AM** requires the molecular motor to rotate beyond a half-turn, supporting the conclusion that the motor undergoes continuous unidirectional rotation rather than acting merely as a photoswitch.

As shown by the HPLC mobility profile (Fig. S15b), UV irradiation of **DNA–4AS** resulted in only a negligible retention-time shift, indicating that half-turn photoisomerization alone failed to drive the DNA into a compact, mechanically constrained conformation. This absence of mechanical caging

was further corroborated by functional assays. Native PAGE analysis (Fig. 3c) demonstrated that UV-irradiated **DNA–4AS** readily hybridized with compDNA, indistinguishable from the non-irradiated control. Likewise, denaturing PAGE analysis (Fig. 3d) showed that UV irradiation of **DNA–4AS** provided no enhanced protection against Exo I digestion relative to the non-irradiated control. Together, these results demonstrate that a half-turn photoisomerization is insufficient to induce mechanical caging of the DNA. Therefore, the pronounced hybridization suppression and enhanced nuclease resistance observed for irradiated **DNA–4AM** must arise from the molecular motor undergoing continuous rotation beyond a half-turn, thereby generating the mechanically constrained DNA conformation responsible for the observed caging effects.

Section XII. Experiments with 0DBCO-DNA

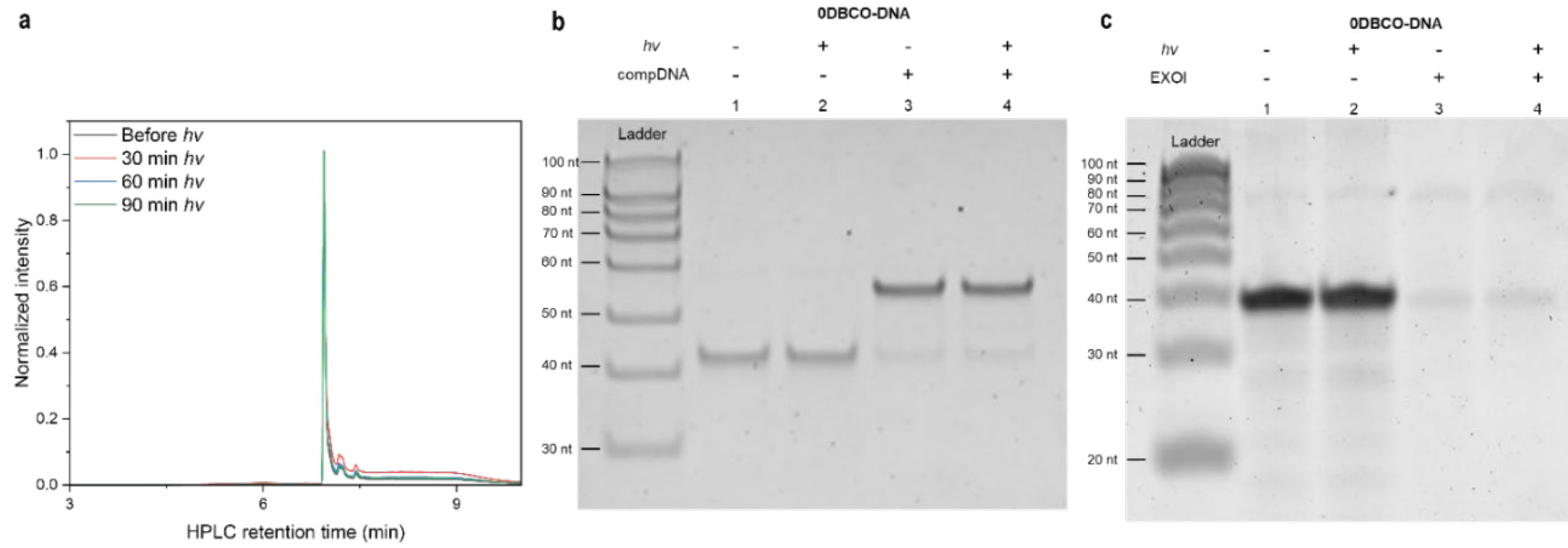

Figure S16. (a) Reversed-phase HPLC analysis (detected at 254 nm) of **0DBCO-DNA** during 365 nm irradiation. (b) Native PAGE (20%, GelRed-stained) analysis of **0DBCO-DNA** subjected to hybridization with compDNA. Lane 1: nascent **0DBCO-DNA**; Lane 2: **0DBCO-DNA** after 365 nm irradiation for 2 h; Lane 3: nascent **0DBCO-DNA** incubated with compDNA in 0.2 M NaCl at room temperature overnight; Lane 4: post-irradiated **0DBCO-DNA** incubated with compDNA. An uncropped image of the original gel is shown in Fig. S66. (c) Denaturing PAGE (20%, GelRed-stained) analysis of **0DBCO-DNA** following Exo I digestion. Lane 1: nascent **0DBCO-DNA**; Lane 2: **0DBCO-DNA** after 365 nm irradiation for 2 h; Lane 3: nascent **0DBCO-DNA** treated with Exo I at 37 °C for 2 hours; Lane 4: post-irradiated **0DBCO-DNA** treated with Exo I. A 20–100 nt ssDNA ladder is shown for size reference. An uncropped image of the original gel is shown in Fig. S67.

Section XIII. HPLC and PAGE Characterization of DNA–2AM

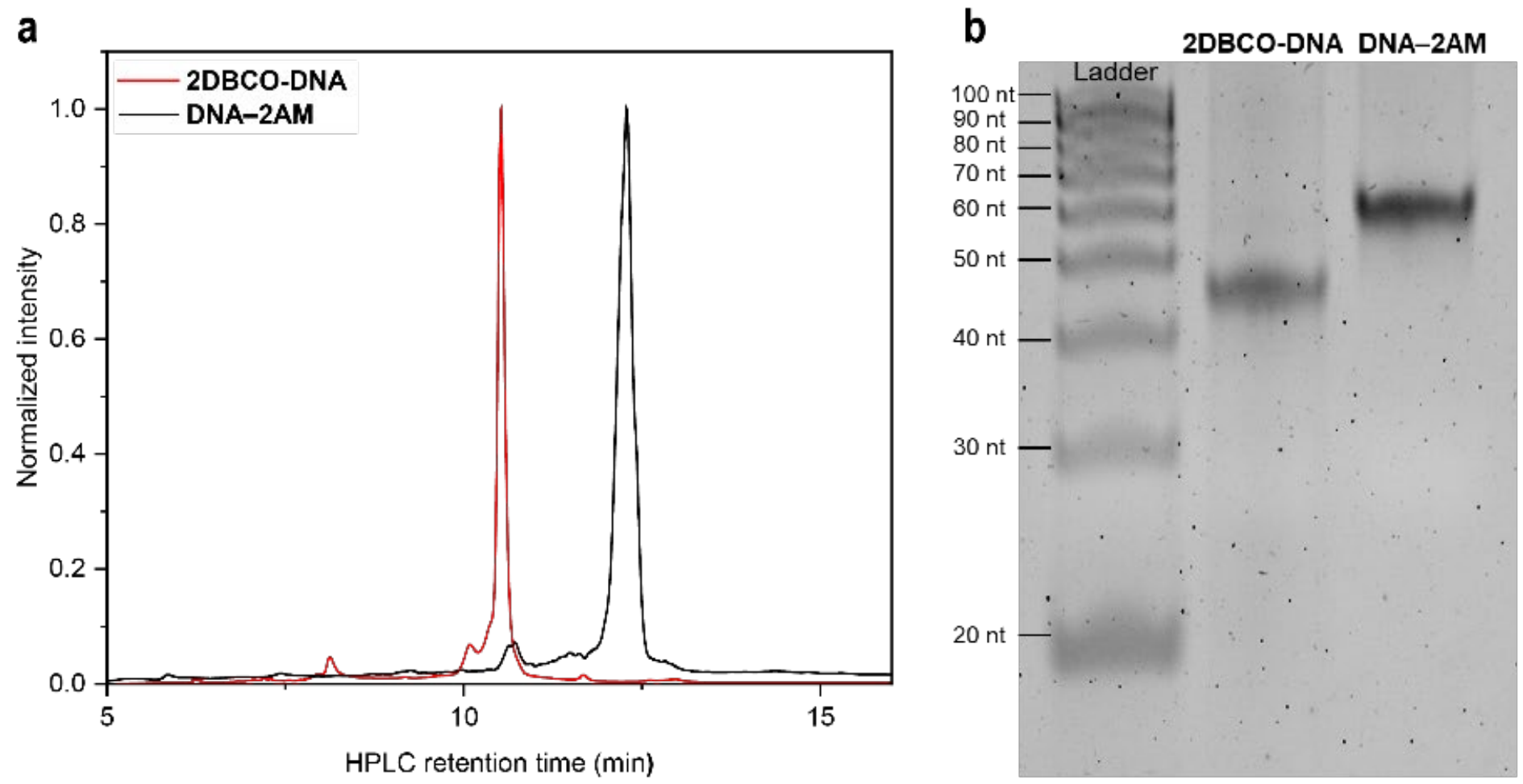

Figure S17. (a) Reversed-phase HPLC analysis (detected at 254 nm) of **2DBCO-DNA** and **DNA–2AM**. (b) Denaturing PAGE (20%, GelRed-stained) analysis of **2DBCO-DNA** and **DNA–2AM**. An uncropped image of the original gel is shown in Fig. S44.

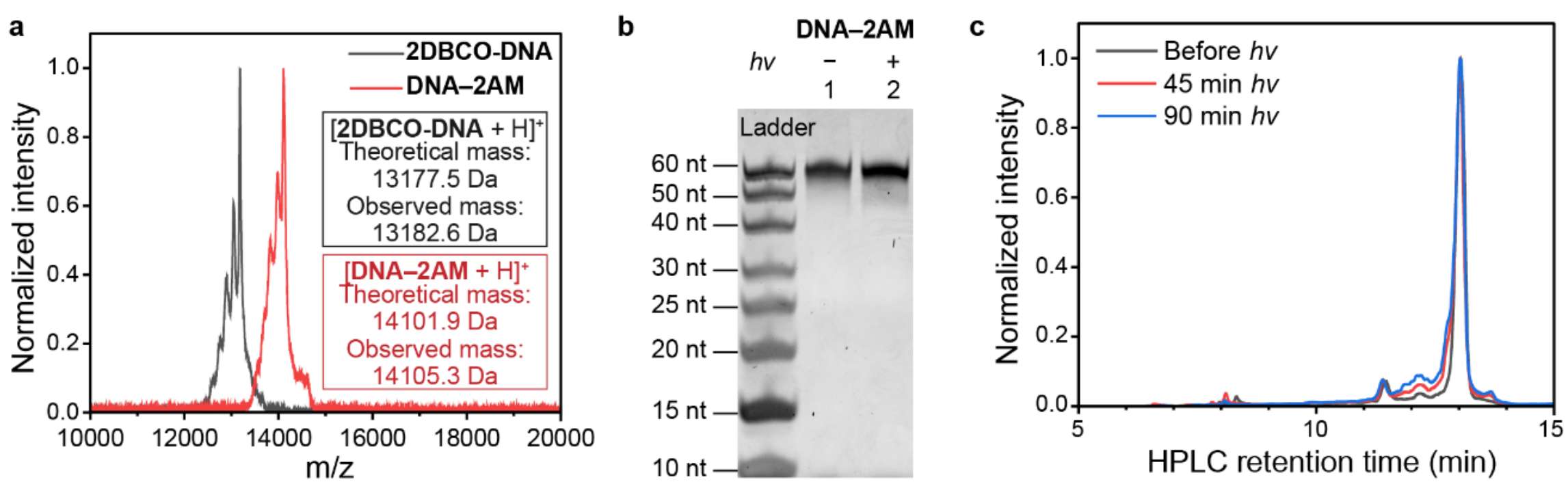

Figure S18. (a) MALDI-MS analysis confirming formation of **DNA–2AM**. (b) Denaturing PAGE (20%, GelRed-stained) analysis of **DNA–2AM** before and after irradiation. Lane 1: nascent **DNA–2AM**; Lane 2: **DNA–2AM** irradiated at 365 nm for 2 h. A 10–60 nt ssDNA ladder is shown for size reference. An uncropped image of the original gel is shown in Fig. S68. (c) Reversed-phase HPLC analysis (detected at 254 nm) showing no detectable difference between nascent and irradiated **DNA–2AM**.

Section XIV. Simulations of Motor–DNA Conjugates

The generalized work (*W*) required to rotate each motor was computed by integrating the applied force over the course of the SMD simulations. We note that, due to the arbitrary manner in which the force is imposed in the SMD simulations, the absolute magnitude of *W* does not carry direct physical significance. However, the trend in *W* as a function of motor rotation provides insight into whether the system experiences progressively increasing resistance—indicative of accumulated mechanical constraints—during rotation. The facile motor rotation in **DNA–2AM** yielded an approximately linear trend in *W* as shown in Fig. S19a. In contrast, all three **DNA–4AM** cases yielded a parabolic trend (Fig. S19b–d).

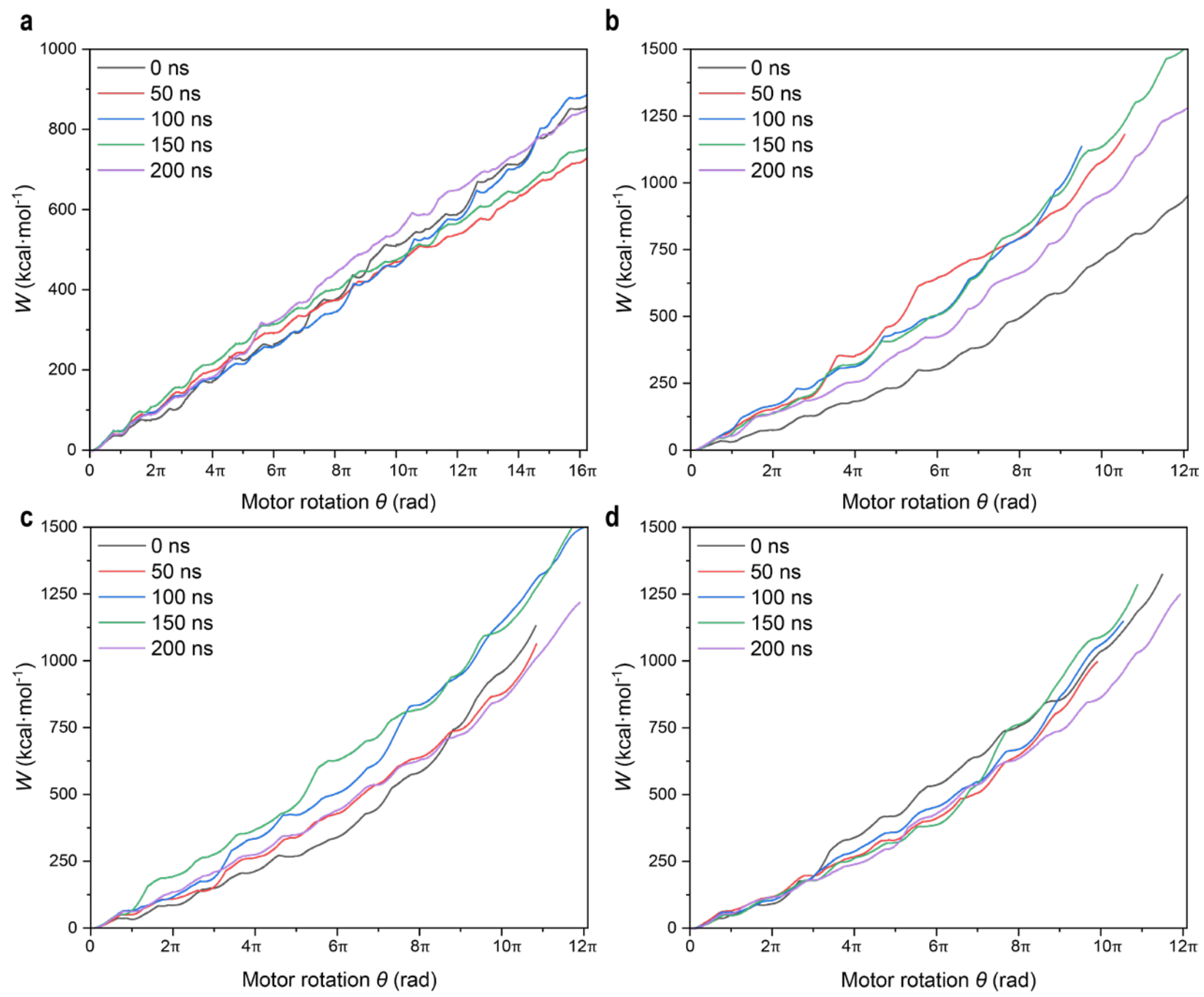

Figure S19. Total work required to rotate (a) **DNA–2AM**, (b) **DNA–4AM** (Case 1), (c) **DNA–4AM** (Case 2), and (d) **DNA–4AM** (Case 3), plotted as a function of *θ*, calculated from five SMD runs initiated from structures sampled at 0, 50, 100, 150, and 200 ns of the corresponding production simulations.

For comparison, we averaged the **DNA–4AM** work curves over the five trials plotted in Fig S20 and performed parabolic fits. As shown in Fig. S20a, there is substantial overlap among the curves for each **DNA–4AM** case, indicating that all three isomers exhibit qualitatively similar winding behavior. This is further exemplified by taking the derivative of *W* (*dW/dθ*), to obtain the torque. As shown in Fig. S20b, the torque increases linearly over the course of **4AM** motor rotation in each case.

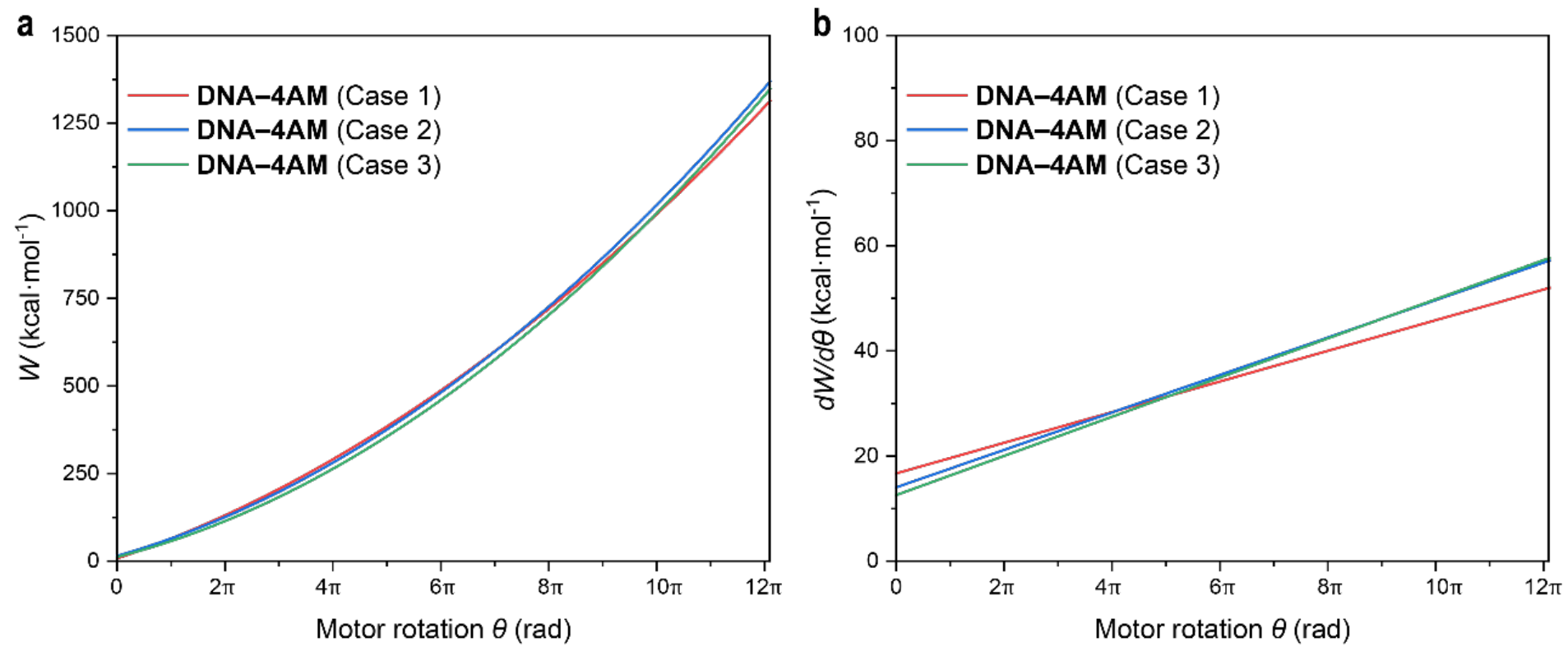

Figure S20. Parabolic fits to the average work curves obtained from SMD simulations. (a) total work (W) required to sustain constant rotational velocity as a function of θ. (b) torque ($dW/d\theta$) as a function of θ, obtained as the derivative of W. All curves represent average fits from five independent SMD simulations.

Umbrella sampling simulations with Hamiltonian replica exchange were used to sample motor rotation under equilibrium conditions. 72 replicas were used to obtain replica exchange acceptance ratios generally above 0.2 as shown in Fig. S21. An exchange bottleneck occurs for **DNA–4AM** when the rotor and stator are in plane, but the acceptance ratio is non-zero.

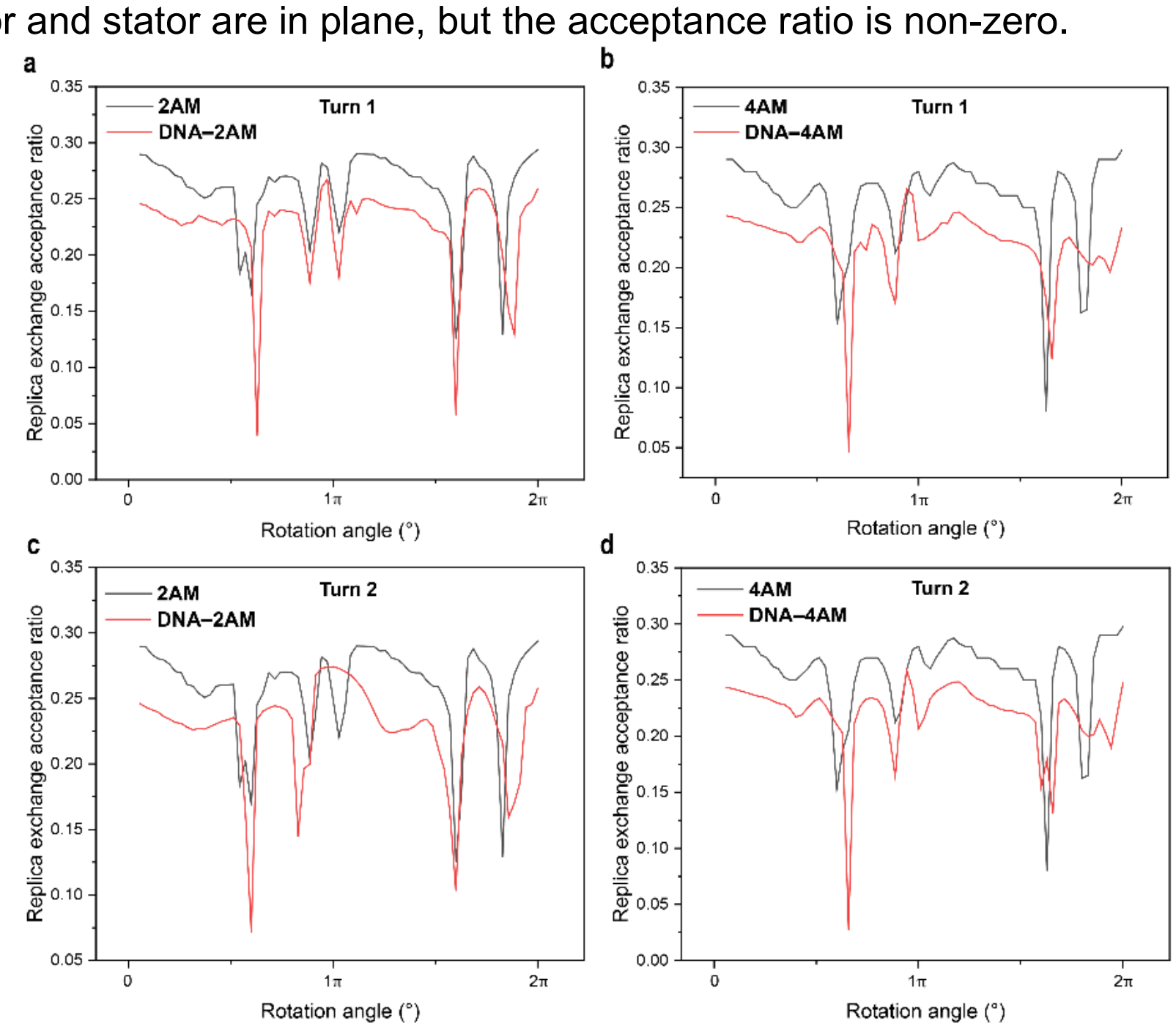

Figure S21. Hamiltonian replica exchange acceptance ratios as a function of rotation angle for (a) Turn 1 of **2AM** and **DNA–2AM**, (b) Turn 1 of **4AM** and **DNA–4AM**, (c) Turn 2 of **2AM** and **DNA–2AM**, and (d) Turn 2 of **4AM** and **DNA–4AM**.

The conformational energy increase of the DNA was extracted from the umbrella sampling simulation results (Fig. 4a) by subtracting the free-energy difference ($\Delta G_{\mathbf{DNA–4AM}} - \Delta G_{\mathbf{4AM}}$) and cumulatively summing the values over three full rotational cycles. The resulting profile (Fig. S22) reveals a steady rise in DNA conformational energy up to approximately 1.5 turns (3π), beyond which the energy increases abruptly. This behavior suggests that the motor might approach a thermodynamic limit for further rotation after roughly 1.5 cycles.

Notably, prior to this abrupt increase, the conformational energy gain of the DNA remains below 15 kcal/mol, which is within the upper bound of the motor's energy output—estimated from the energy of a 365 nm photon minus the typical fluorescence energy of the strained motor (461–469 nm) [36]. Any deeper quantitative interpretation of these absolute energy values would constitute overanalysis, as umbrella sampling is inherently statistical mechanical in nature and does not provide quantum-chemically rigorous energy determinations.

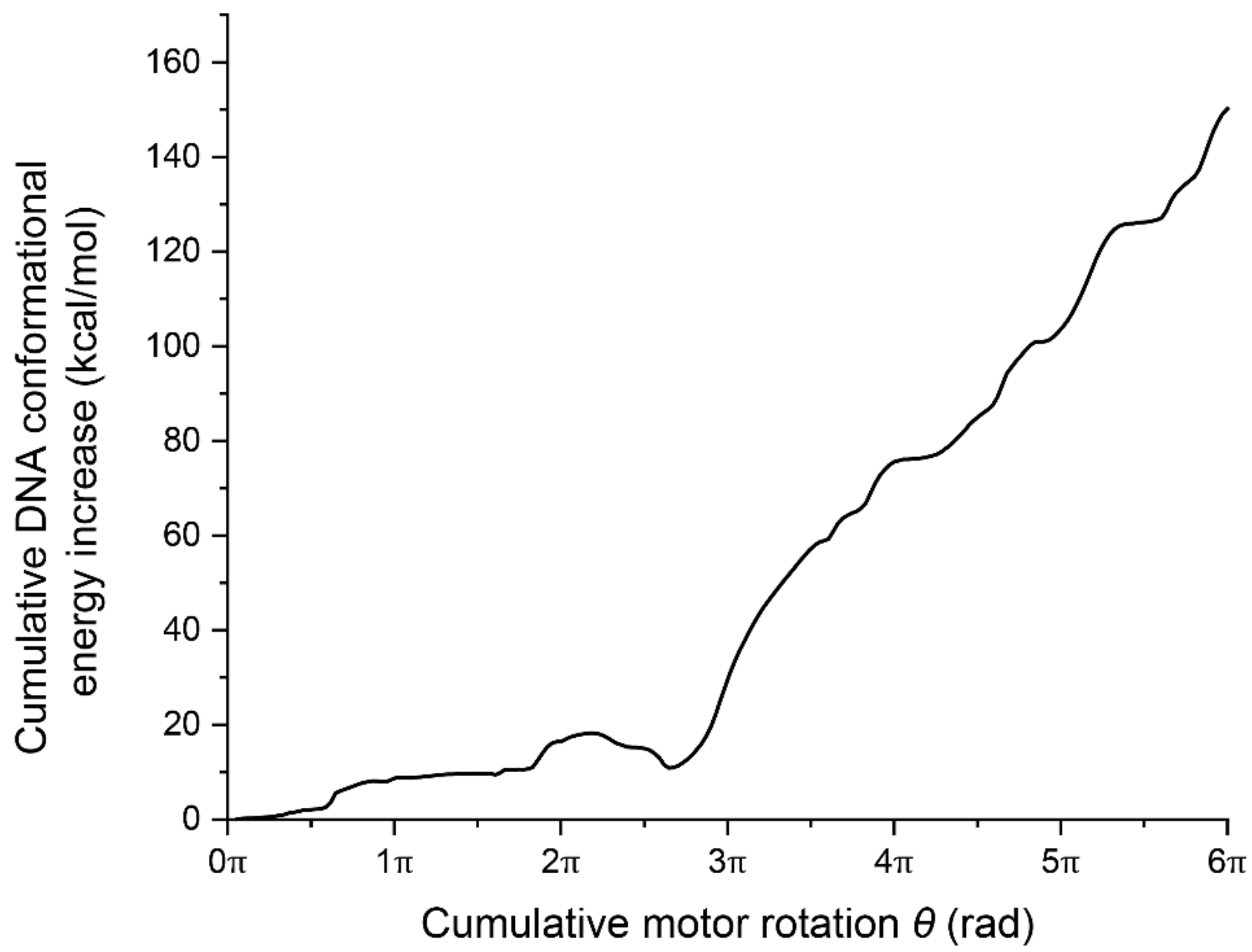

Figure S22. Cumulative increase in DNA conformational energy as a function of motor rotation, extracted from the umbrella sampling simulation results.

The mechanical constraint of ssDNA is reflected in the base stacking overlap area, which serves as a proxy for its helical character [37-38]. As shown in Table S4, **DNA–2AM** exhibits a slight increase in overall helicity after motor rotation (positive Δturn), whereas **DNA–4AM** shows reduced helicity (negative Δturn). The stacking overlap area correlates with the ability of ssDNA to hybridize with a complementary strand—smaller values generally indicate a lower propensity to form duplex DNA. These findings align with our experimental observation that motor rotation in **DNA–4AM** impairs its ability to hybridize with the complementary strand.

Table S4. Mean overlap area (Å^2) from umbrella sampling between stacked nucleobases before and after 1.5 motor rotation cycles.

	**All DNA bases**			**Complementary region**		
	Before turning	After turning	Δturn	Before turning	After turning	Δturn
**DNA–2AM**	5.66	5.73	0.07	5.52	5.90	0.38
**DNA–4AM** (Case 2)	5.56	5.46	-0.1	5.19	4.99	-0.20

[a]Δturn = After turning – Before turning. Means are taken from four umbrella sampling trials.

Section XV. A Reversible System via Cleavable Tether Design

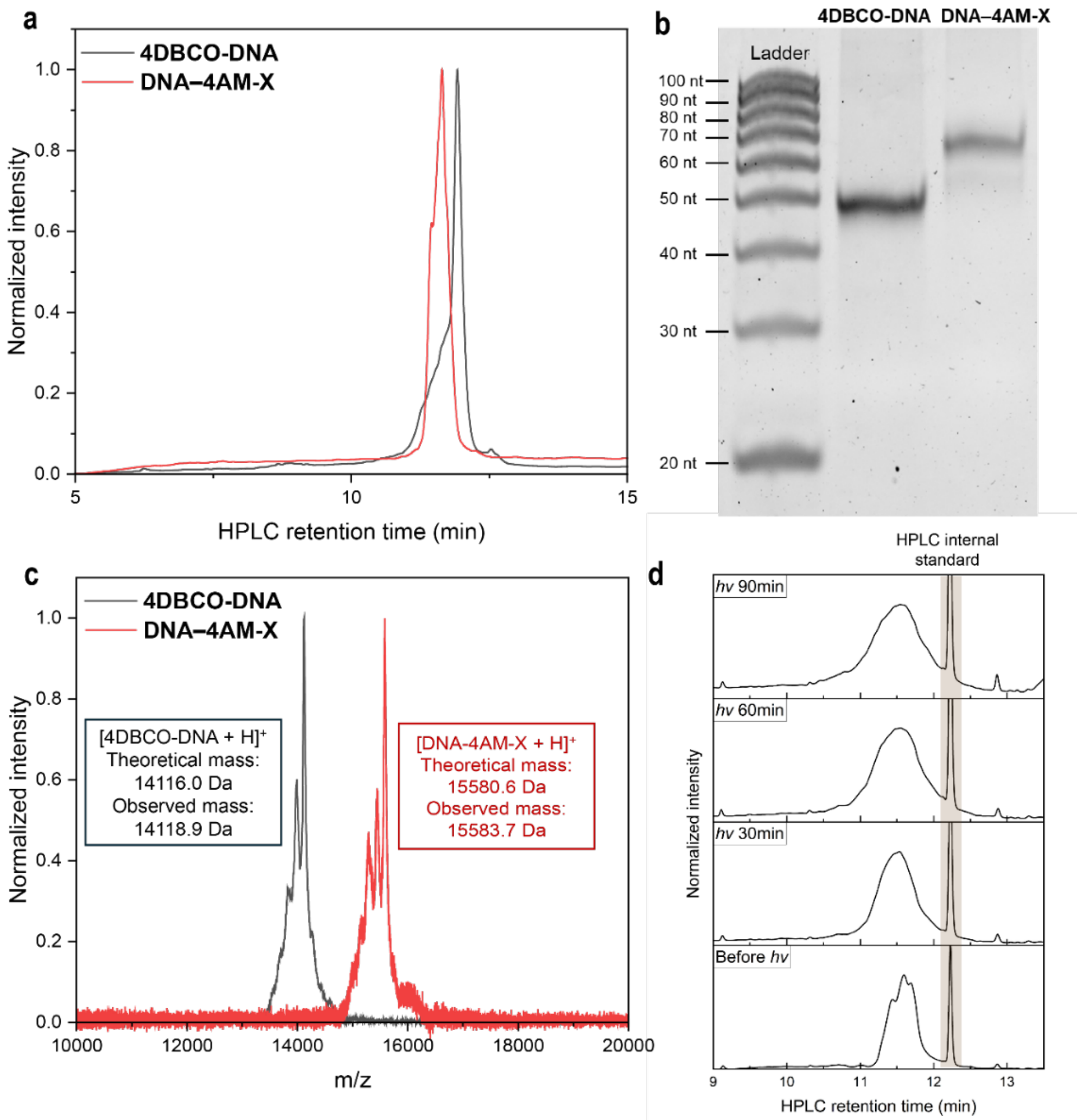

Figure S23. (a) Reversed-phase HPLC analysis (detected at 254 nm) of **4DBCO-DNA** and **DNA–4AM-X**. (b) Denaturing PAGE (20%, GelRed-stained) analysis of **4DBCO-DNA** and **DNA–4AM-X. A** 20–100 nt ssDNA ladder was included for size reference. An uncropped image of the original gel is shown in Fig. S69. (c) MALDI-MS analysis of **4DBCO-DNA** and **DNA–4AM-X**. (d) Reversed-phase HPLC analysis (detected at 254 nm) of **DNA–4AM-X** during 365 nm irradiation.

Section XVI. Spectroscopic Data

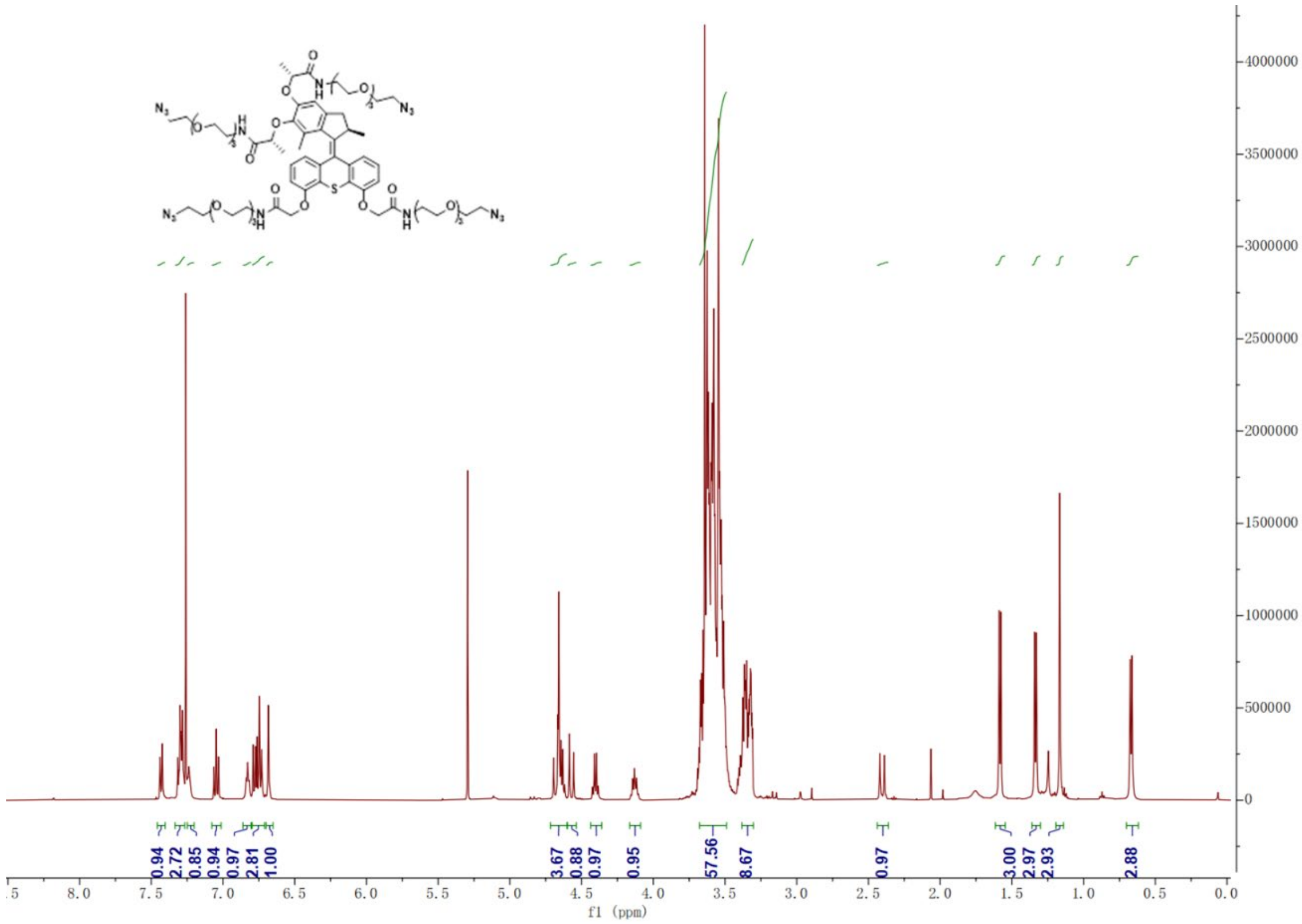

Figure S24. ^{1}H NMR spectrum of **Compound 3** (**4AM**)

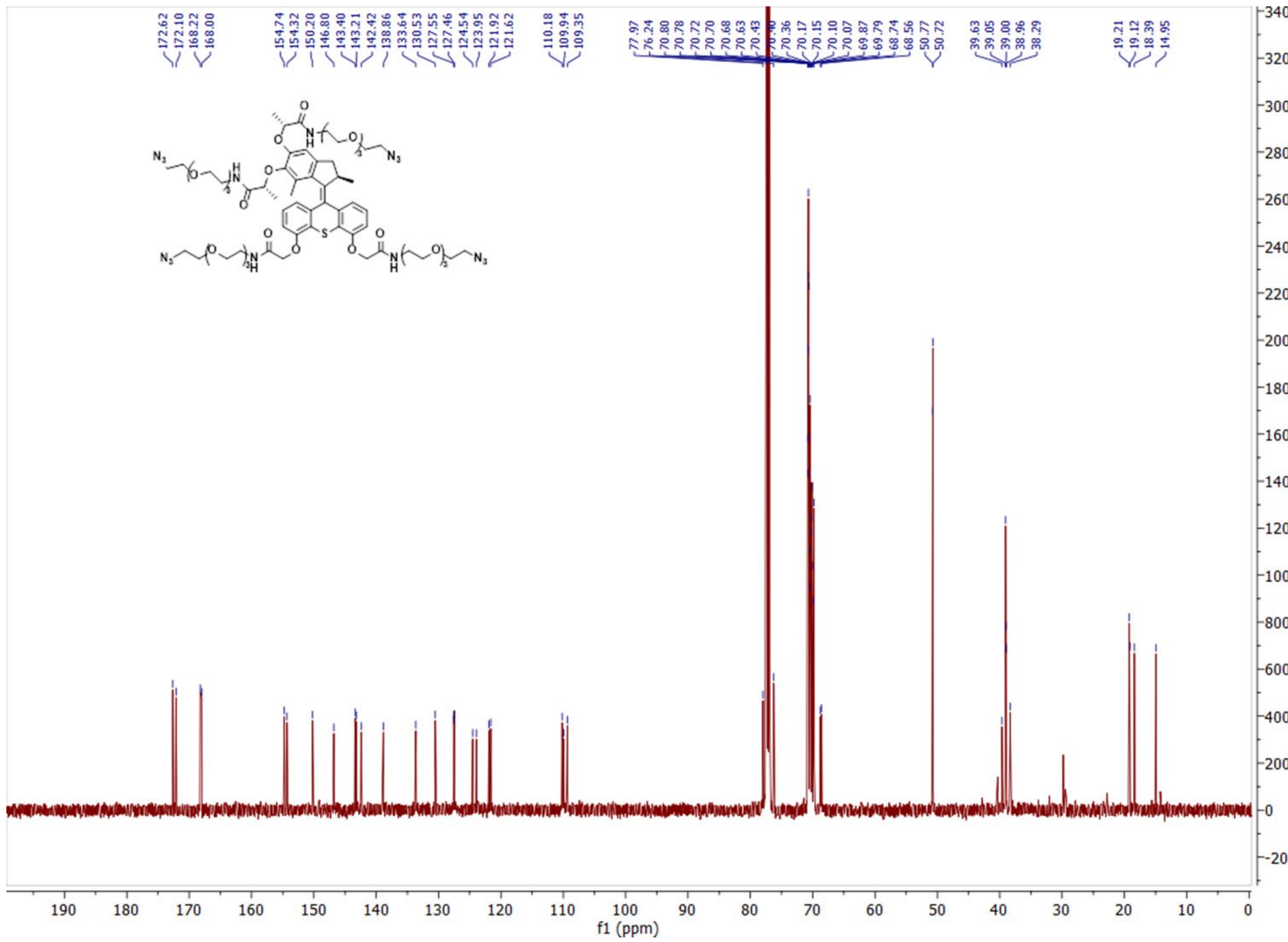

Figure S25. ^{13}C NMR spectrum of **Compound 3** (**4AM**)

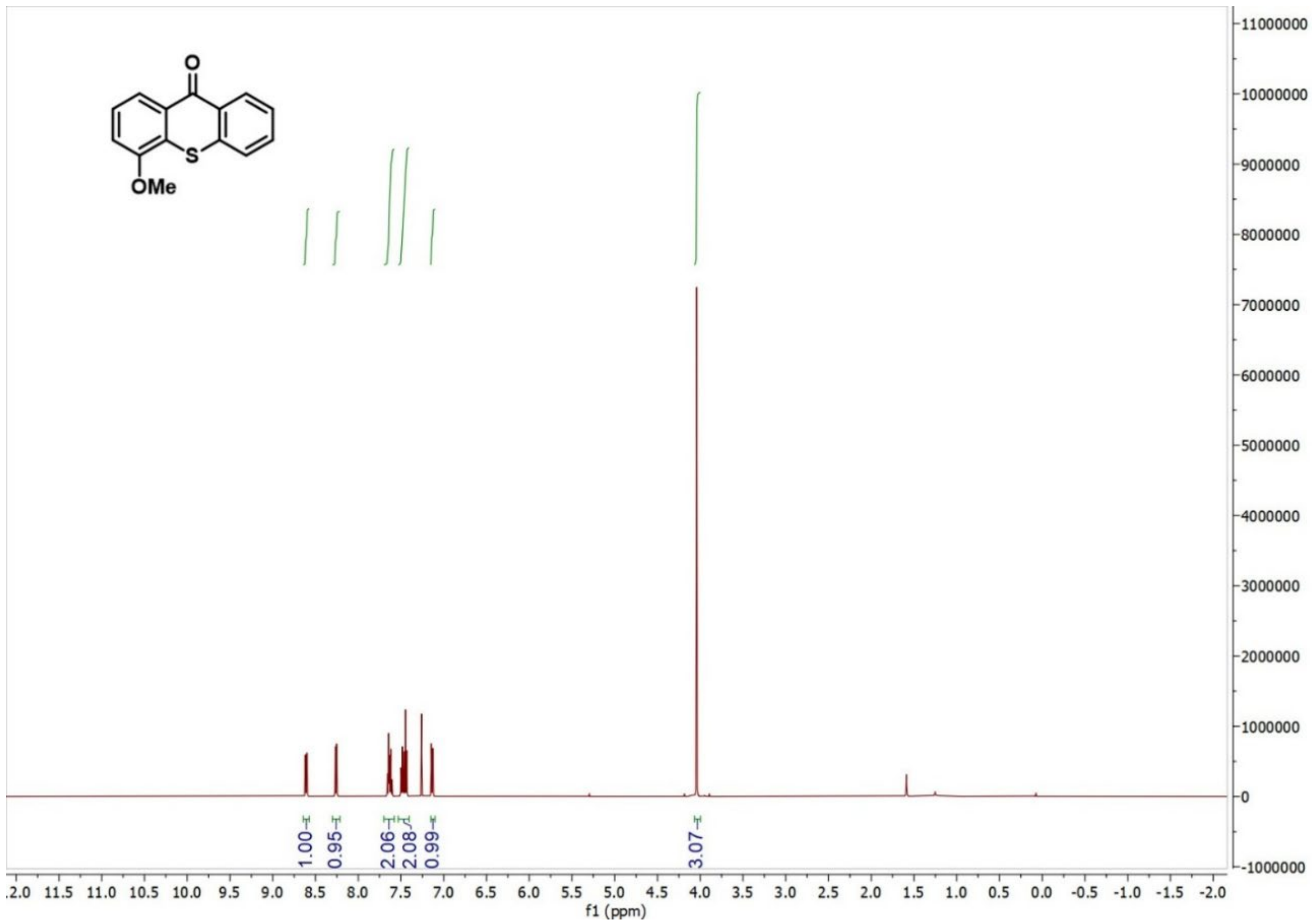

Figure S26. ^{1}H NMR spectrum of **Compound 6**

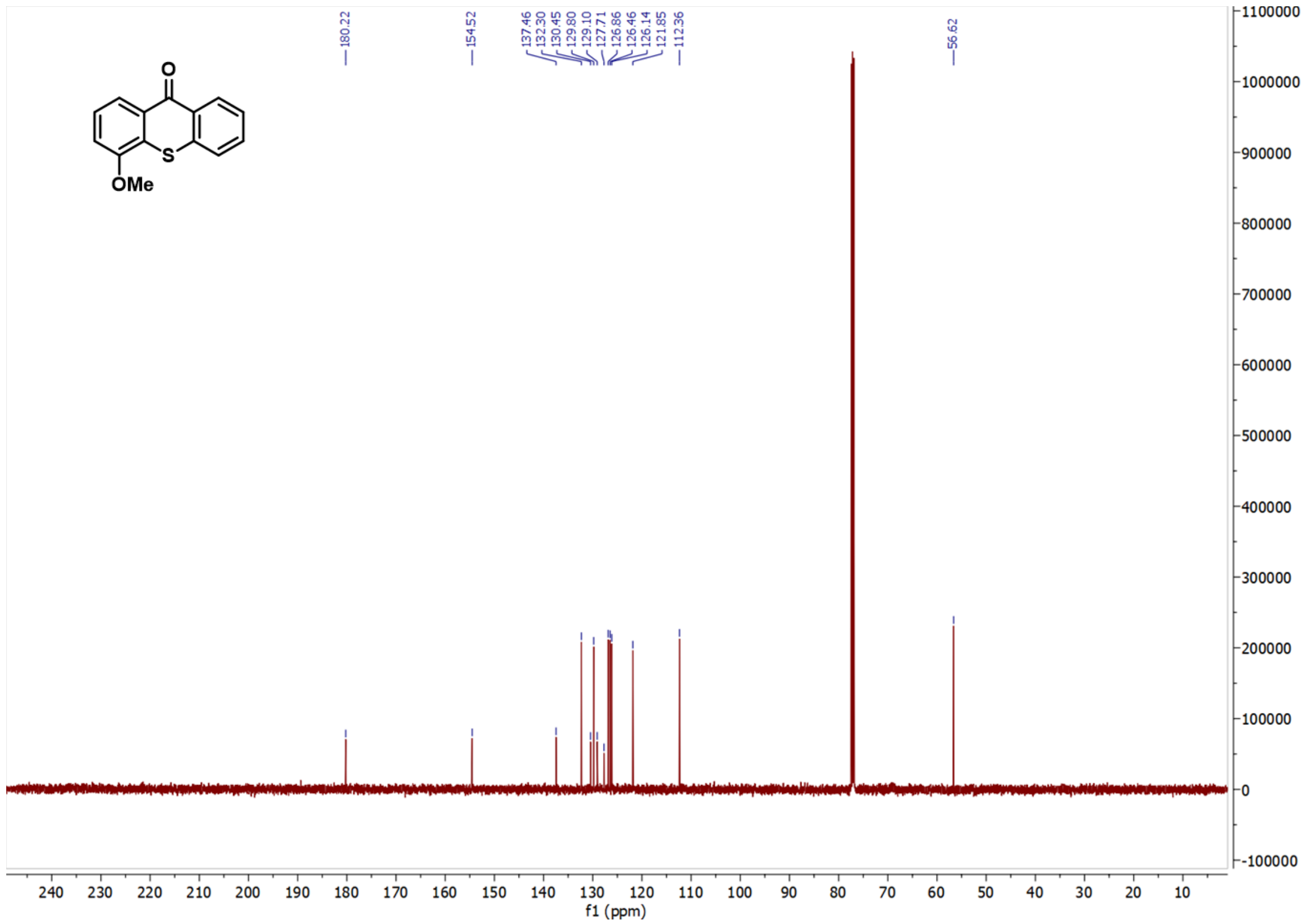

Figure S27. ^{13}C NMR spectrum of **Compound 6**

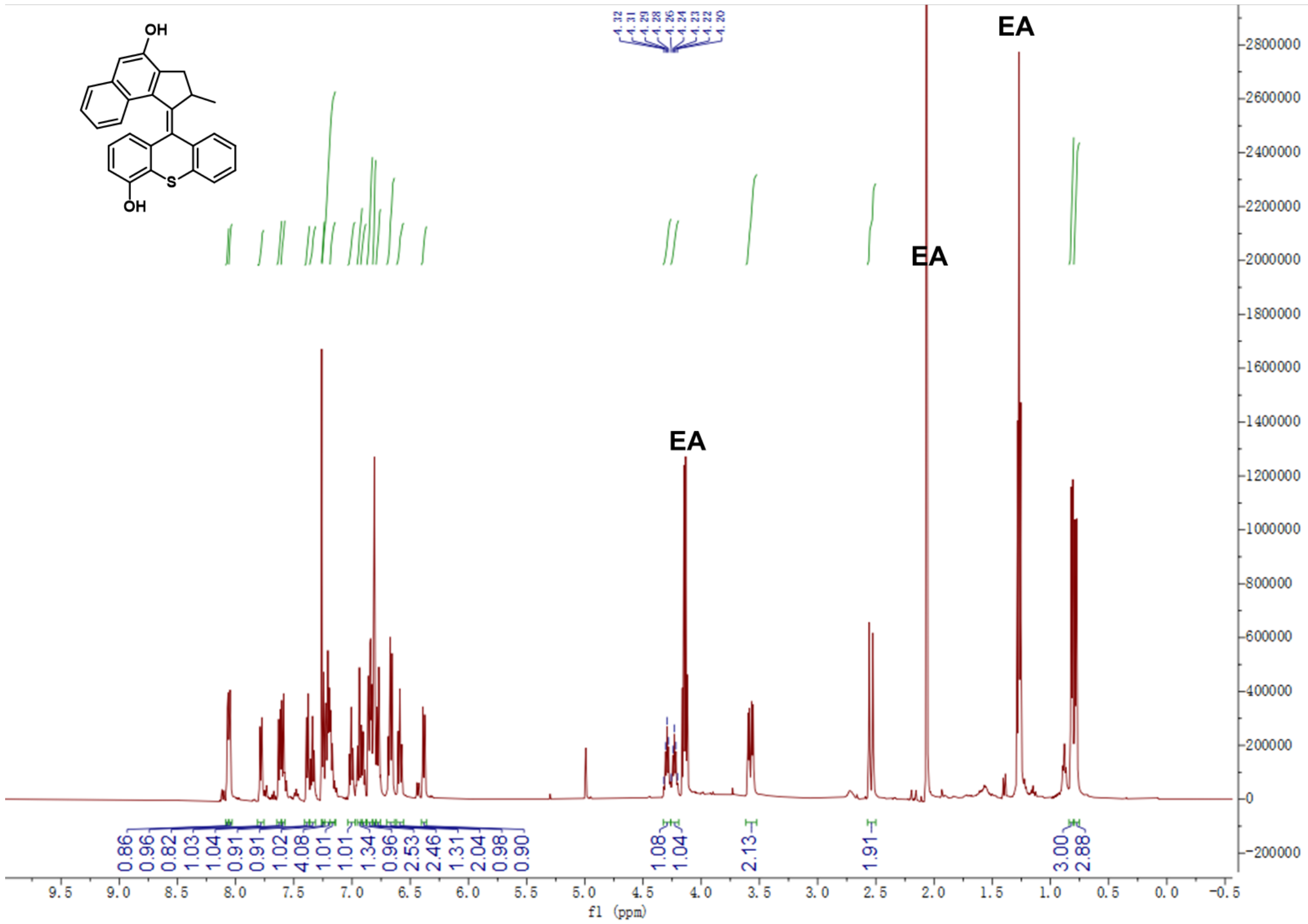

Figure S28. ^{1}H NMR spectrum of **Compound 12**

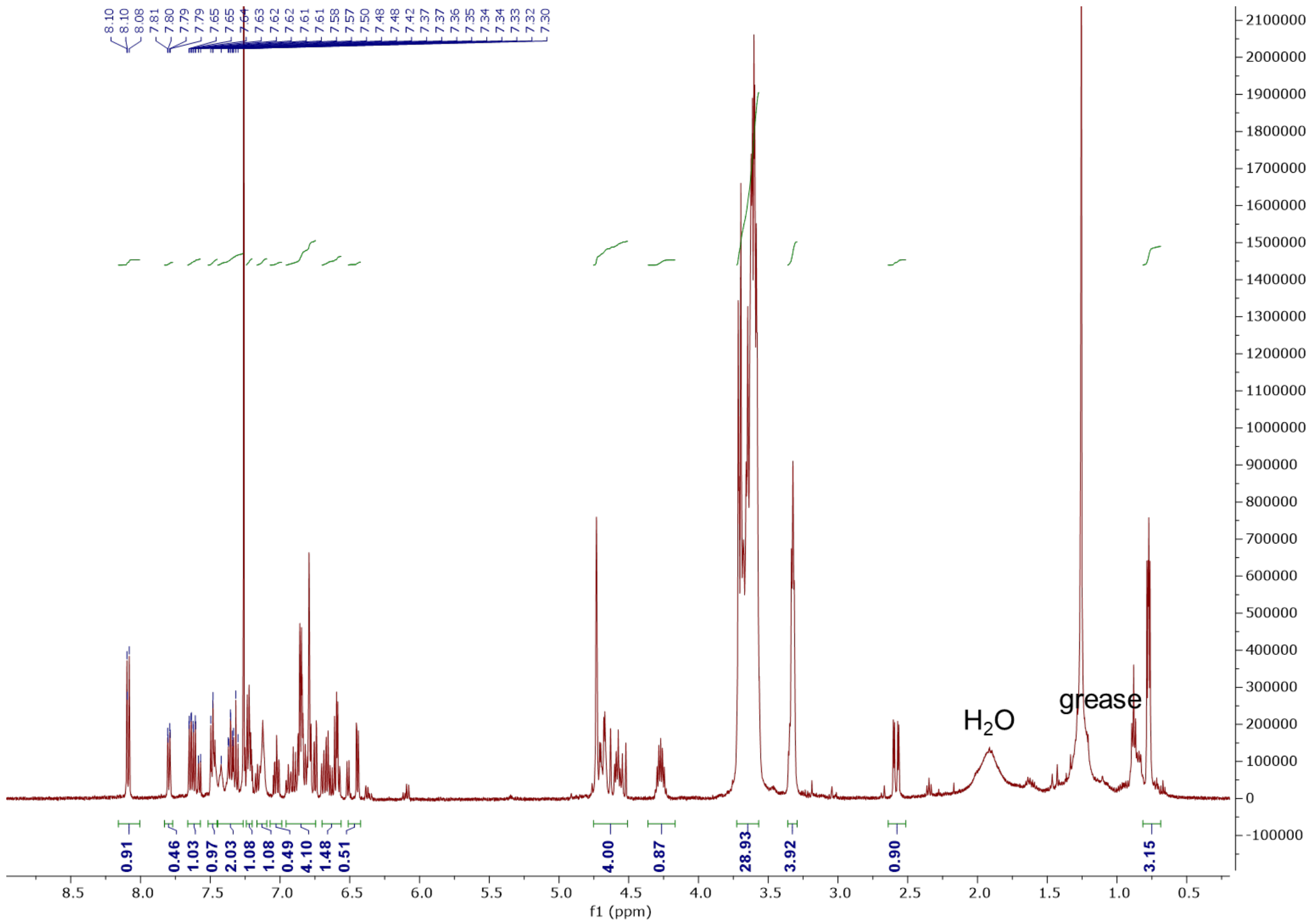

Figure S29. ¹H NMR spectrum of **Compound 14** (**2AM**)

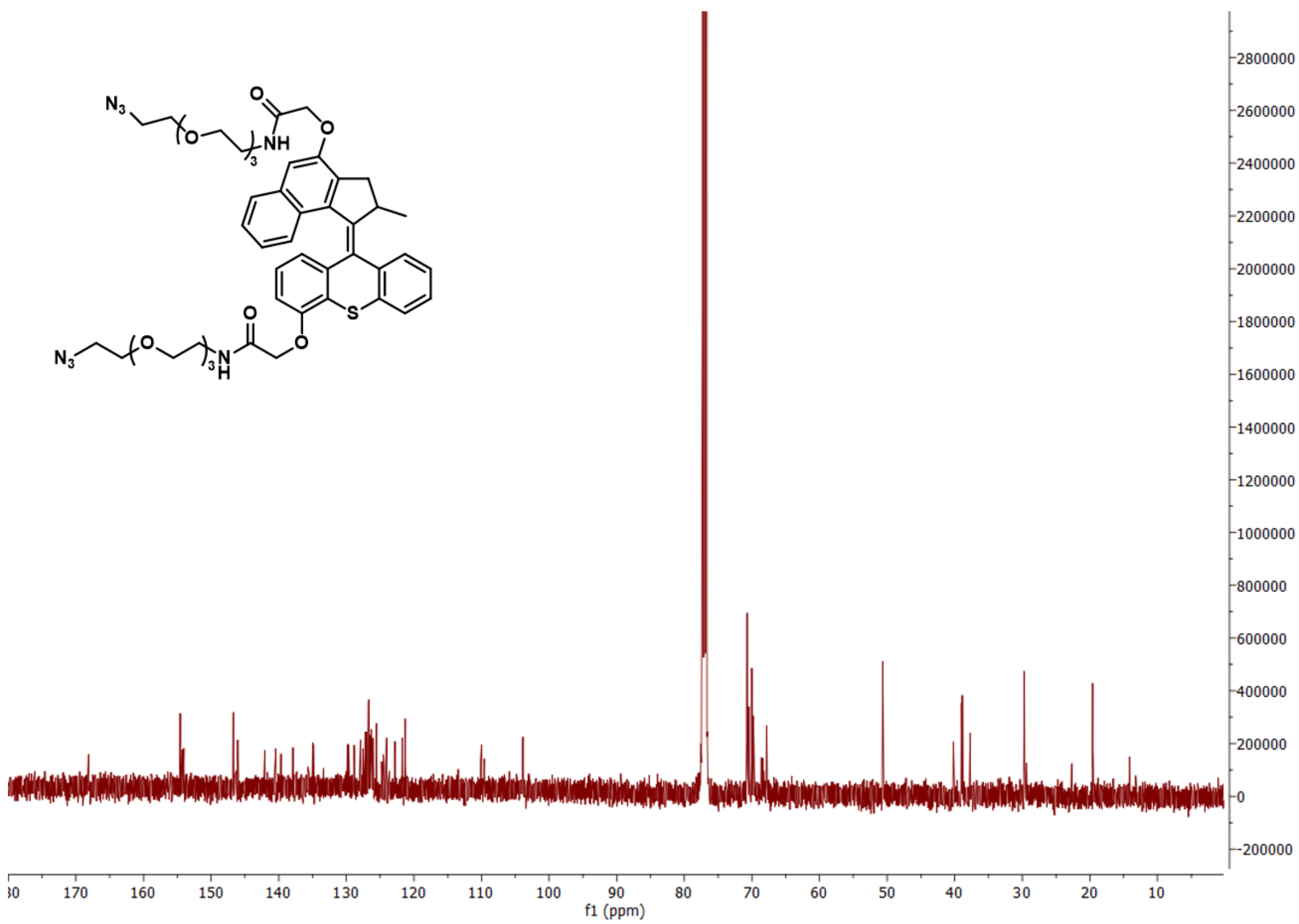

Figure S30. ^{13}C NMR spectrum of **Compound 14** (**2AM**)

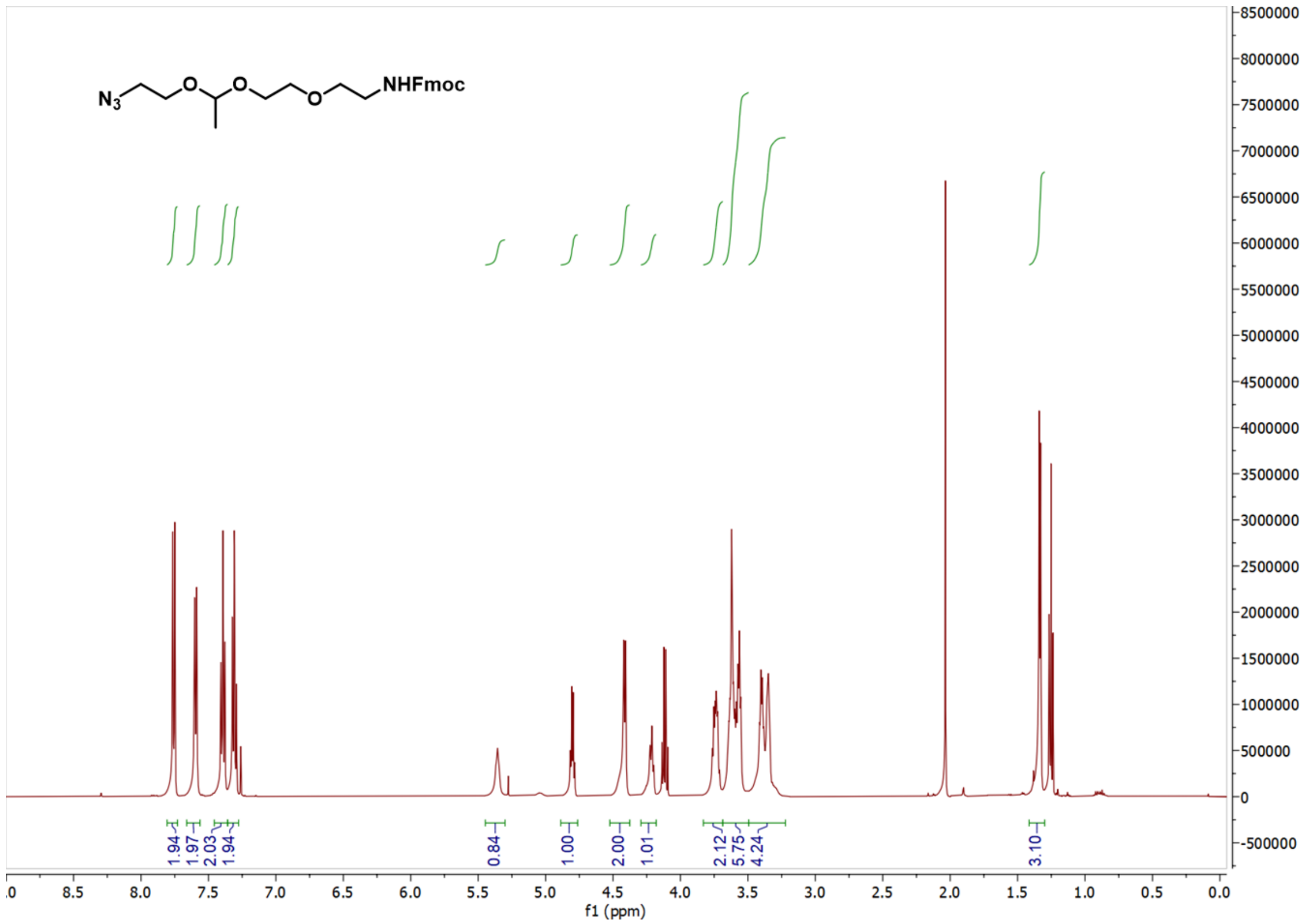

Figure S31. ^{1}H NMR spectrum of **Compound 16**

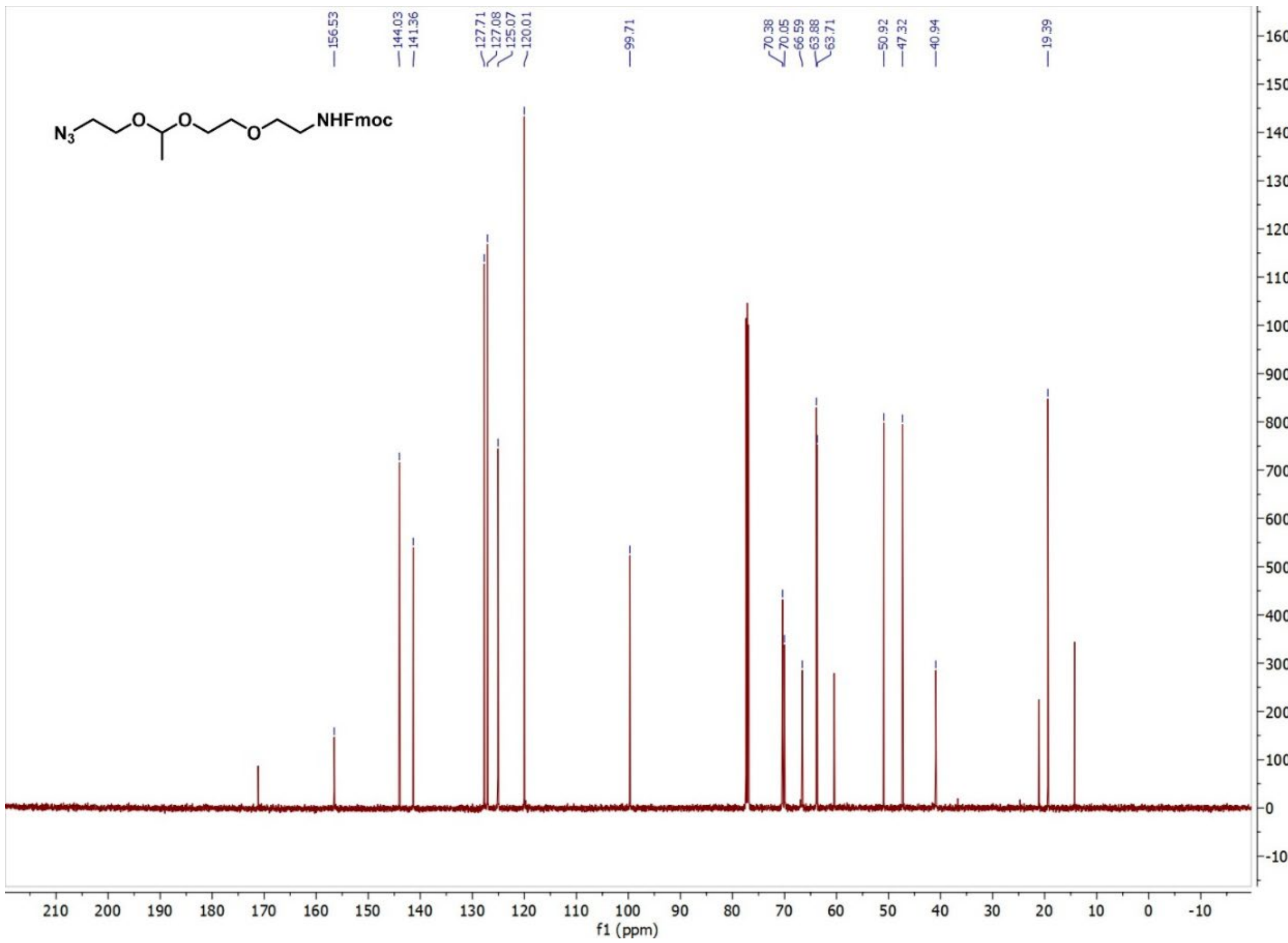

Figure S32.^{13}C NMR spectrum of **Compound 16**

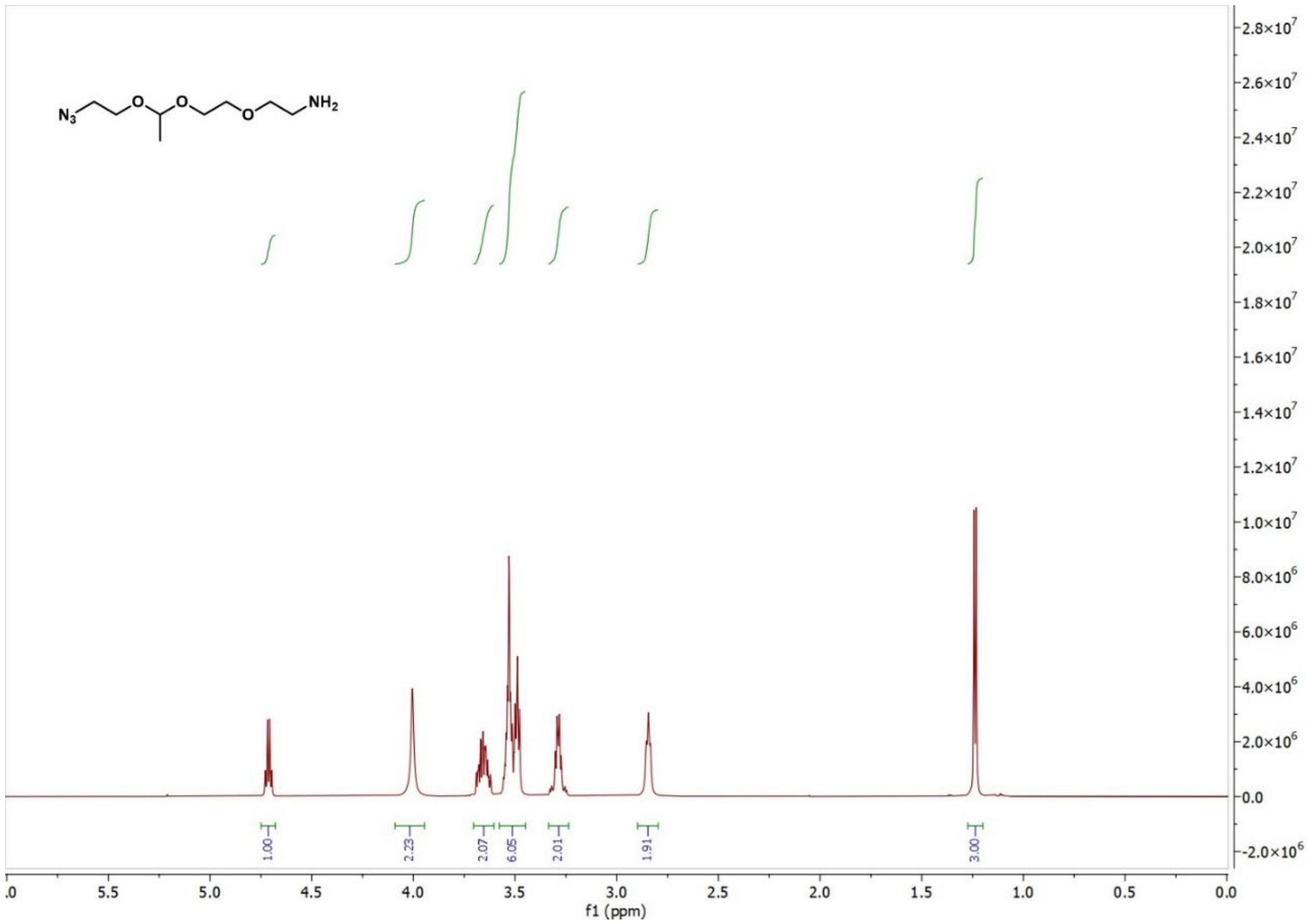

Figure S33. ^{1}H NMR spectrum of **Compound 17**

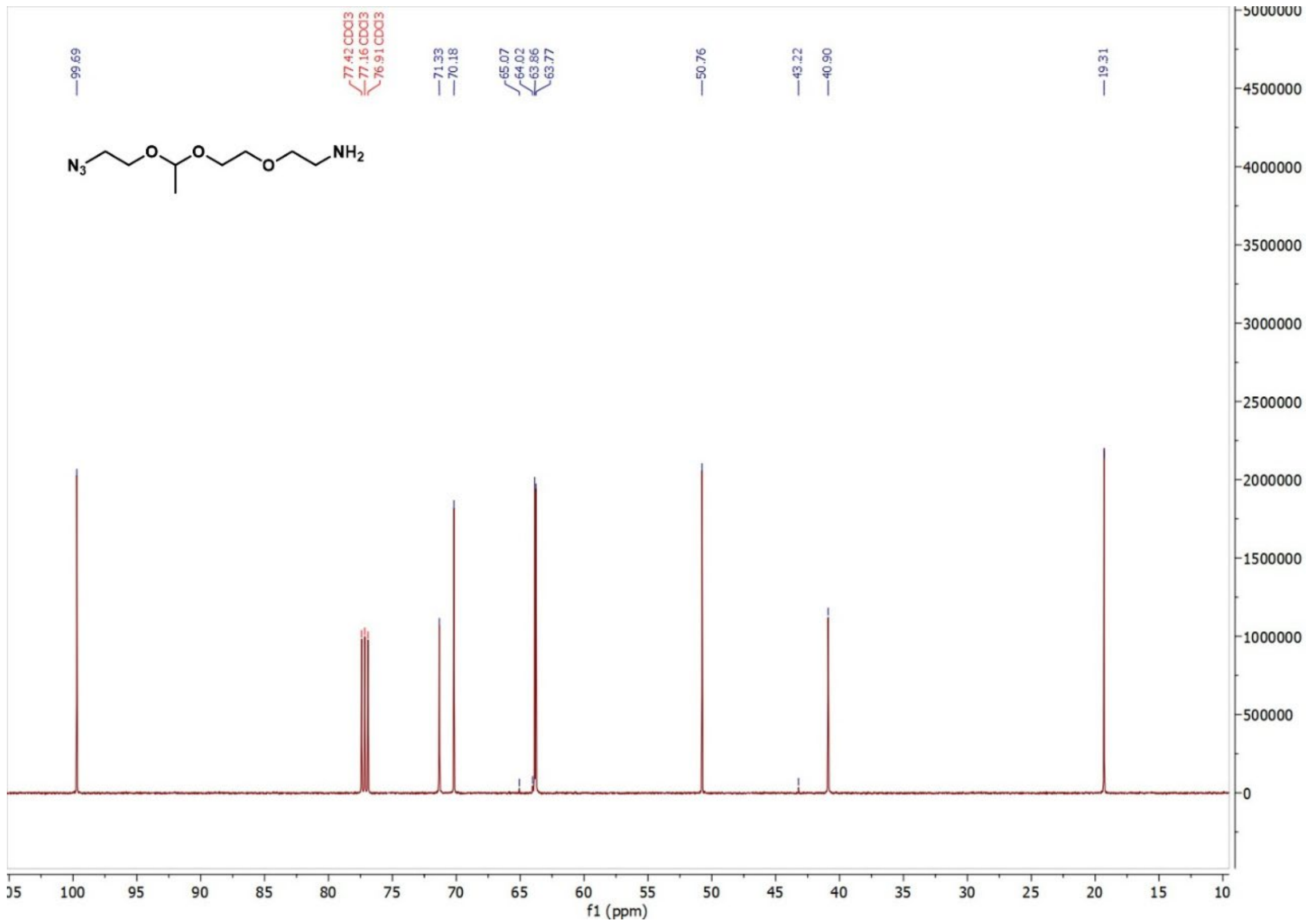

Figure S34. ^{13}C NMR spectrum of **Compound 17**

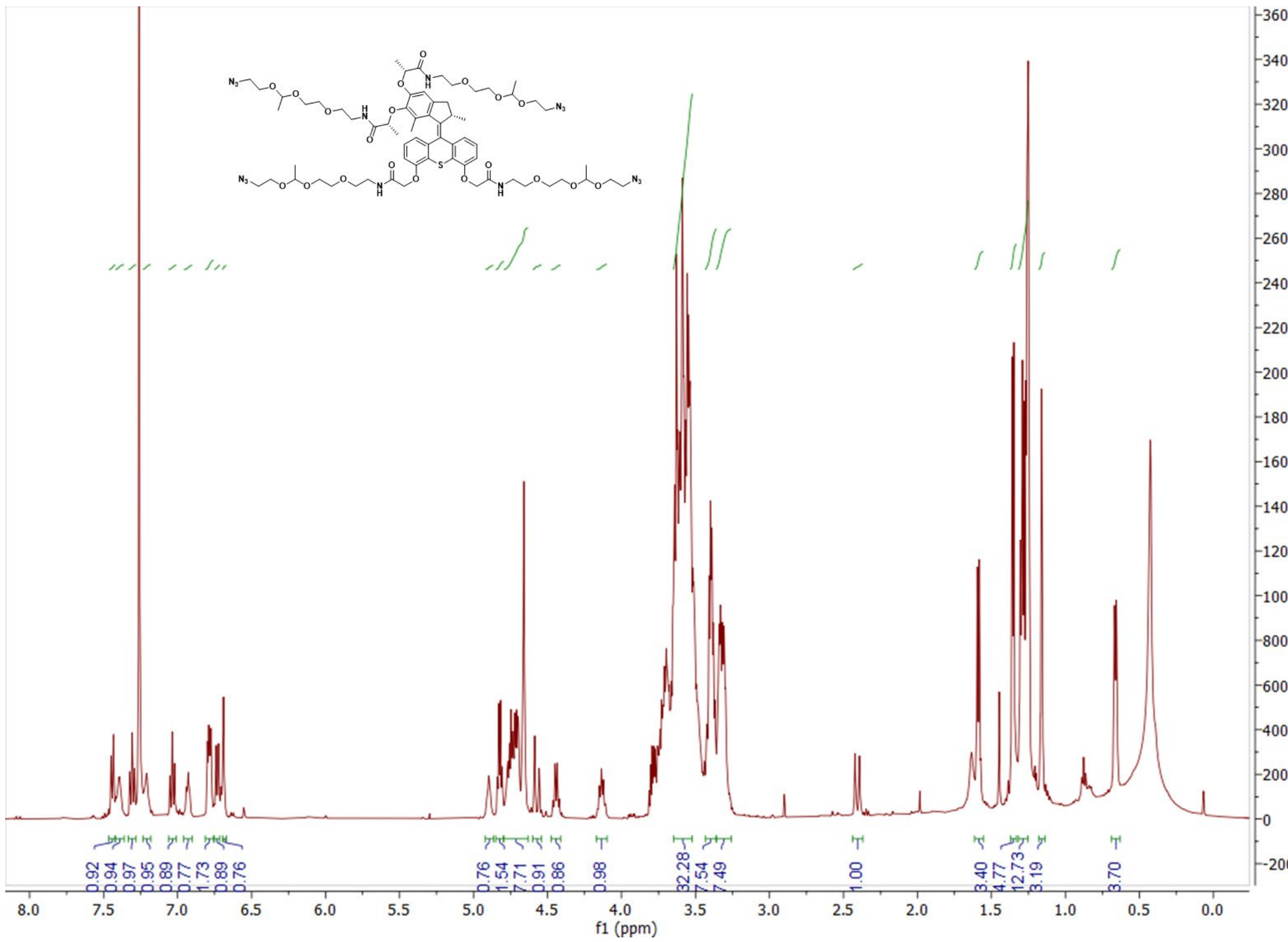

Figure S35. ^{1}H NMR spectrum of **Compound 18** (**4AM-X**)

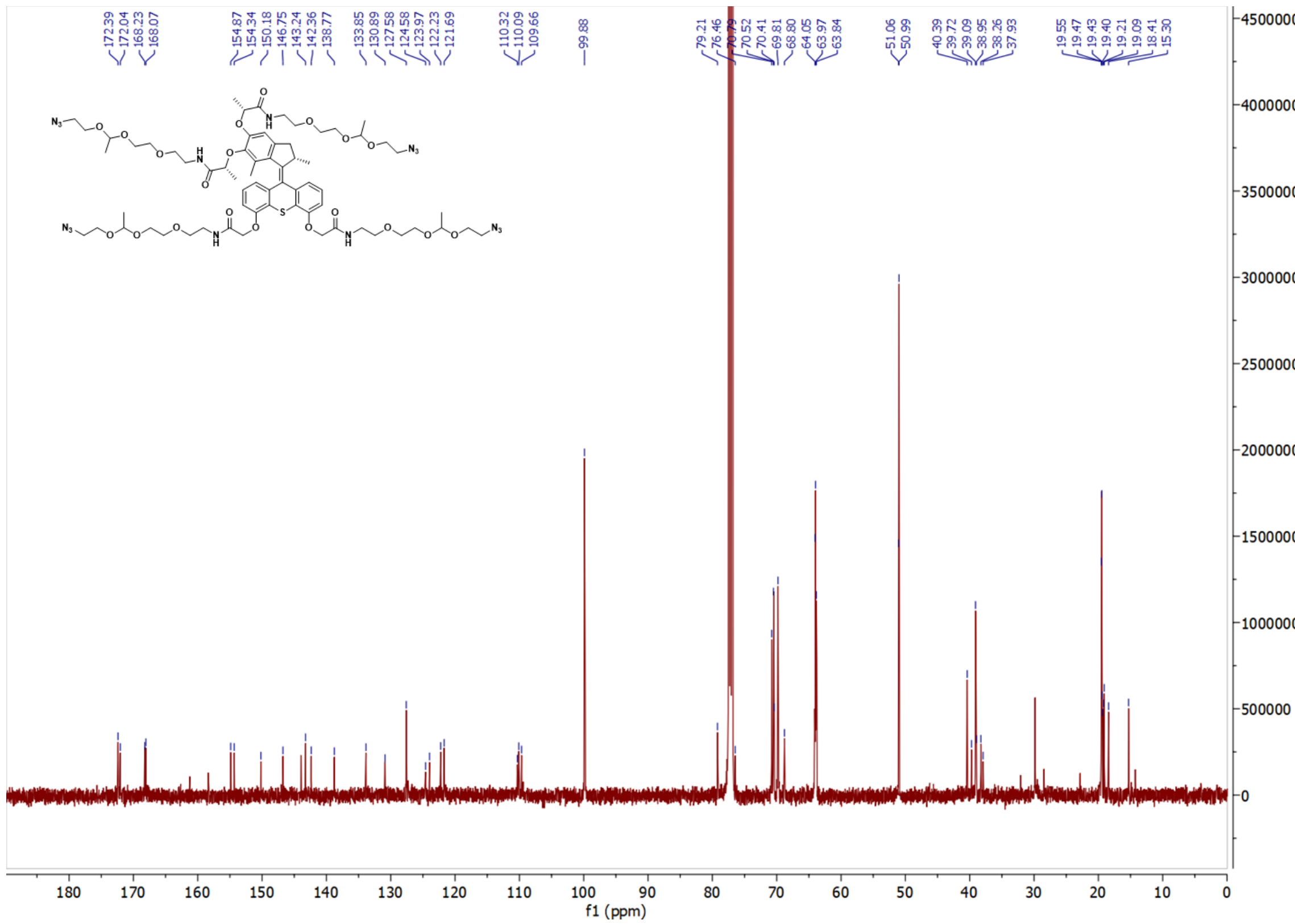

Figure S36. ^{13}C NMR spectrum of **Compound 18** (**4AM-X**)

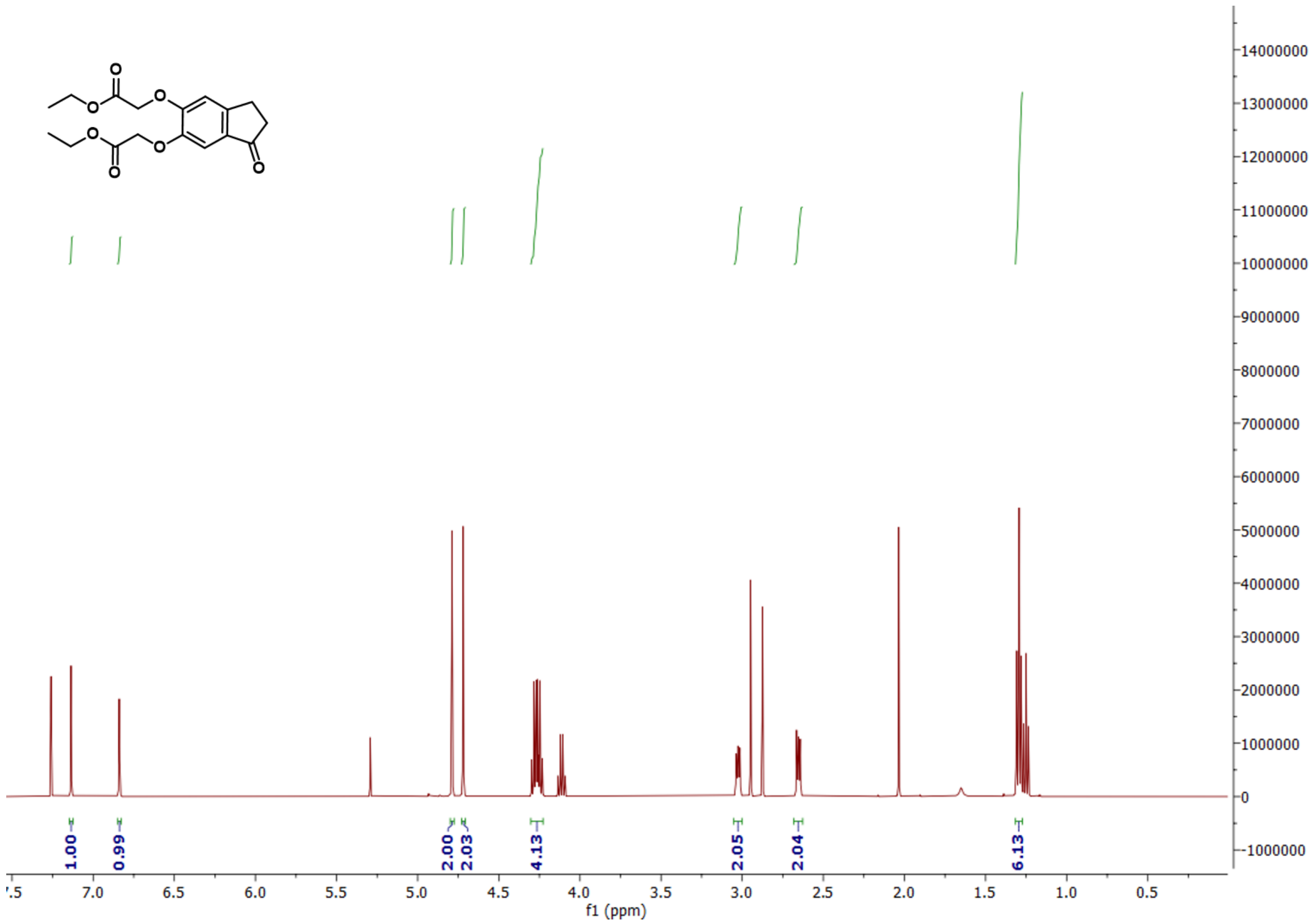

Figure S37. ^{1}H NMR spectrum of **Compound 20**

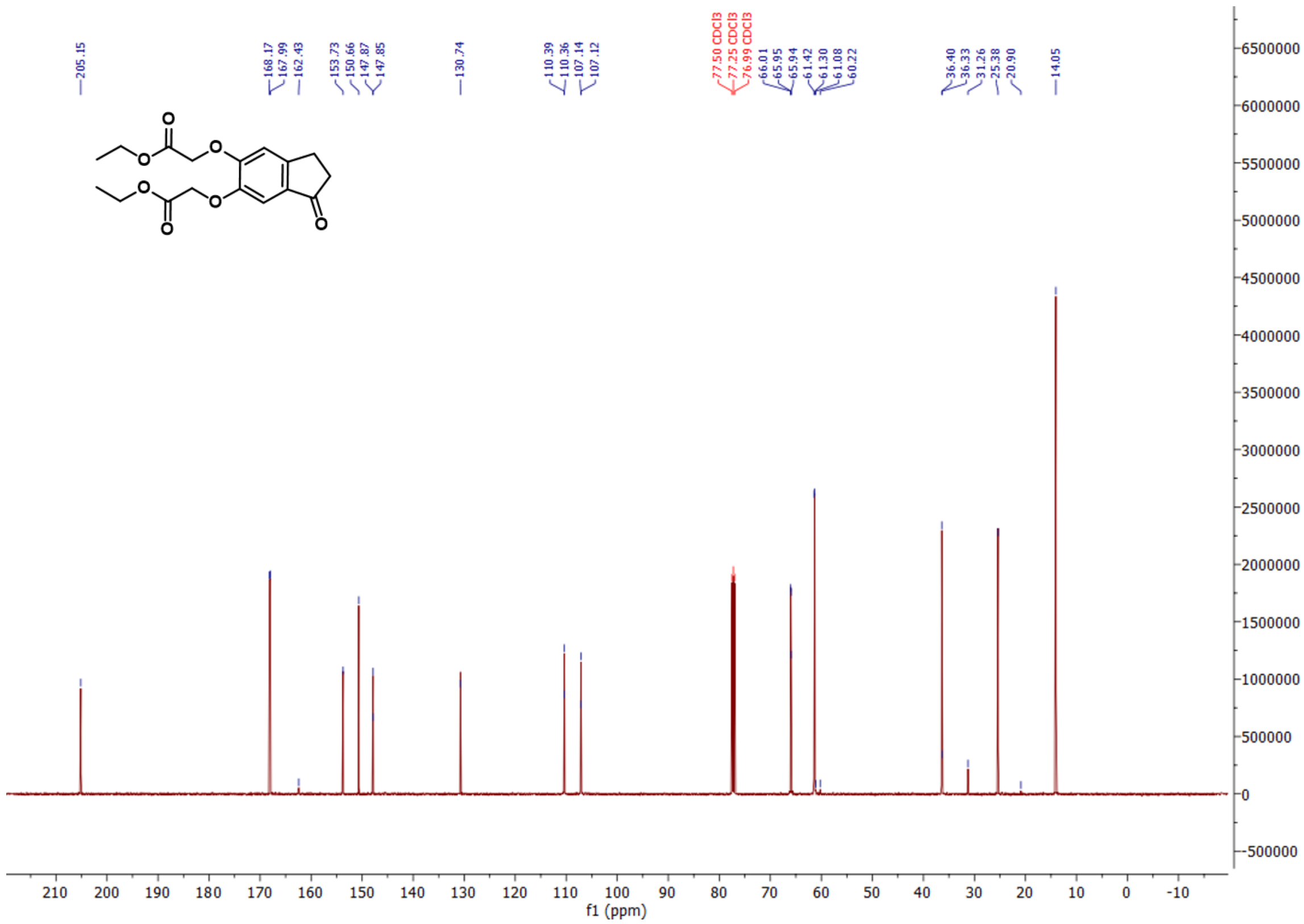

Figure S38.^{13}C NMR spectrum of **Compound 20**

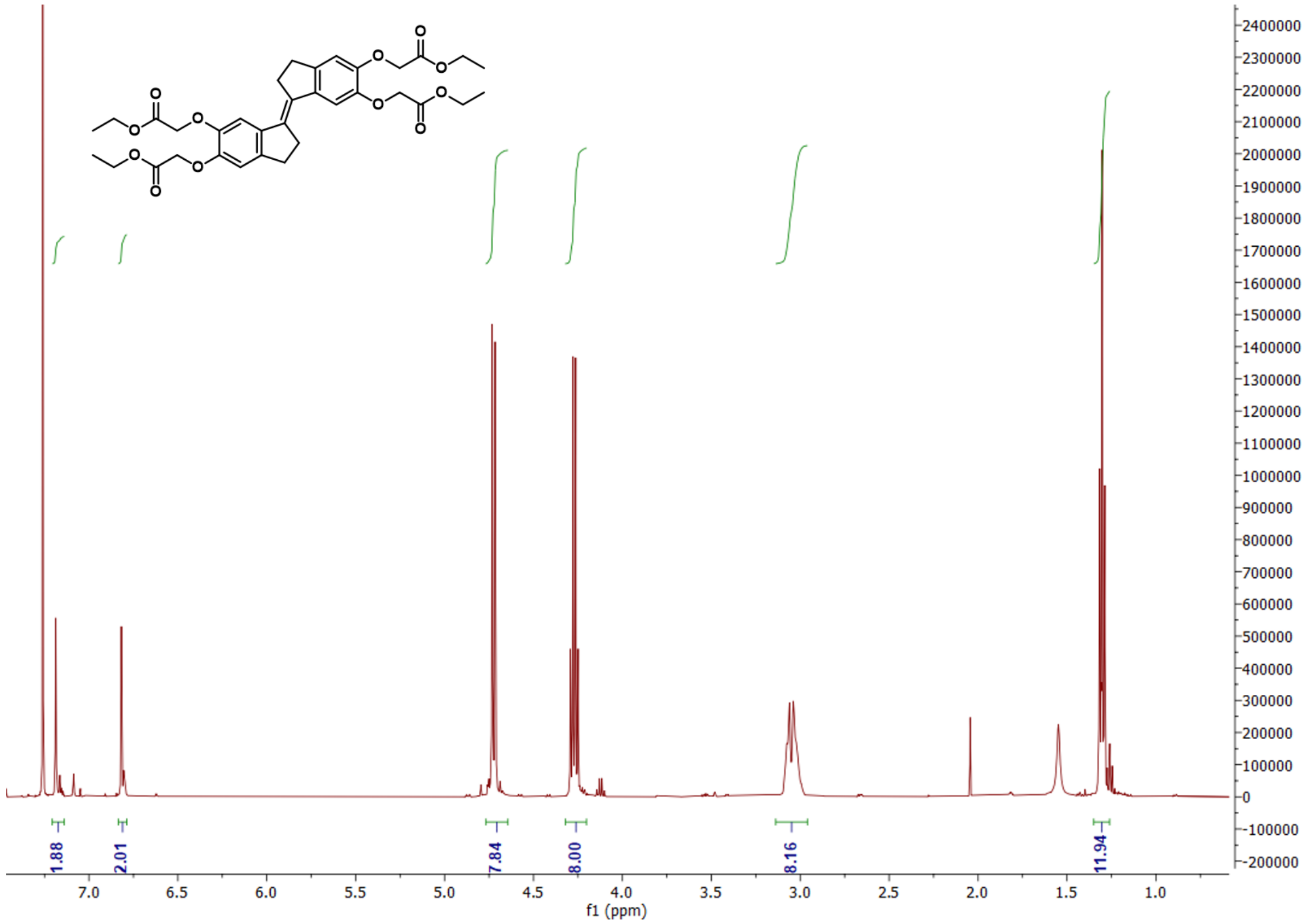

Figure S39. ^{1}H NMR spectrum of **Compound 21**

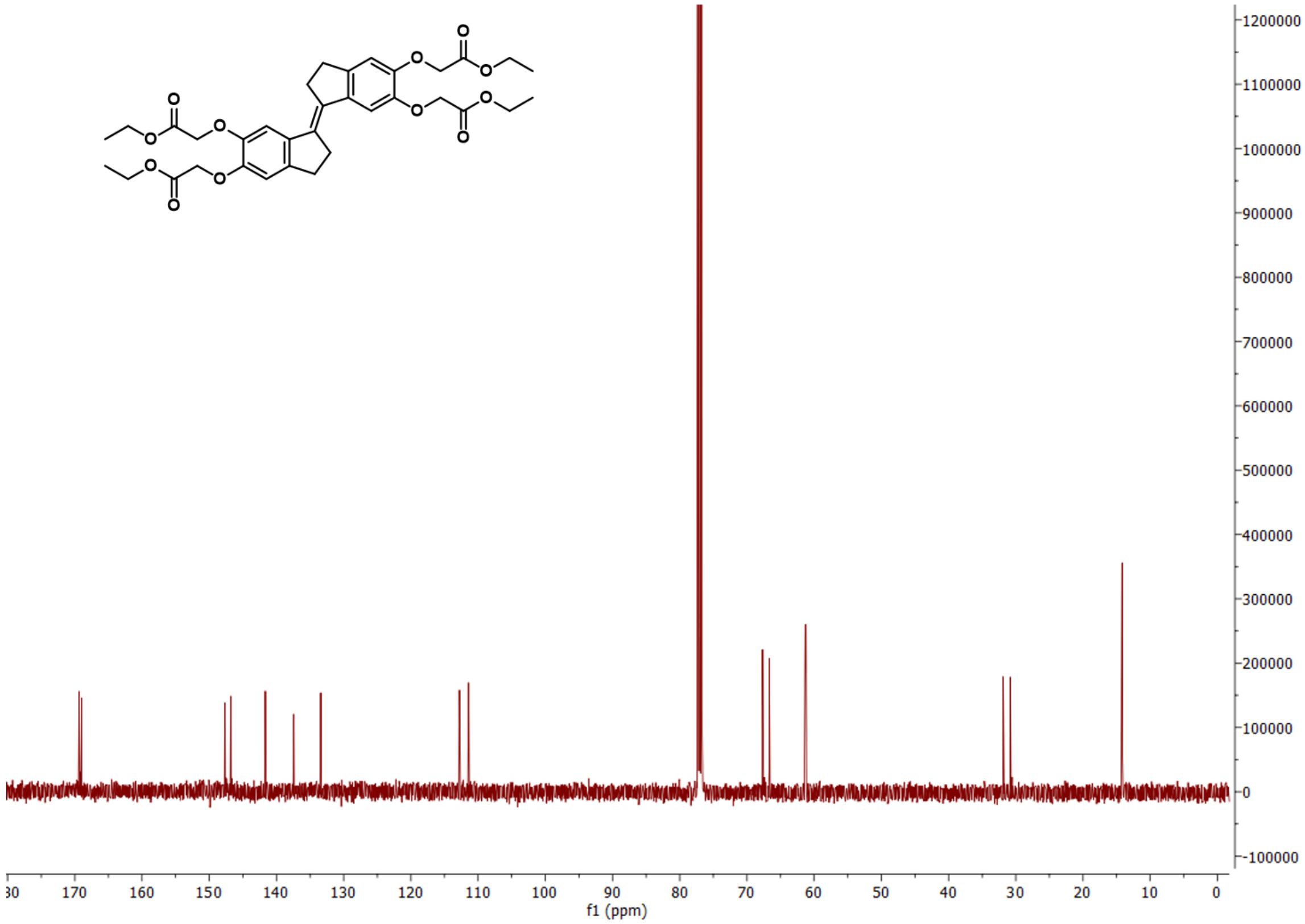

Figure S40. ^{13}C NMR spectrum of **Compound 21**

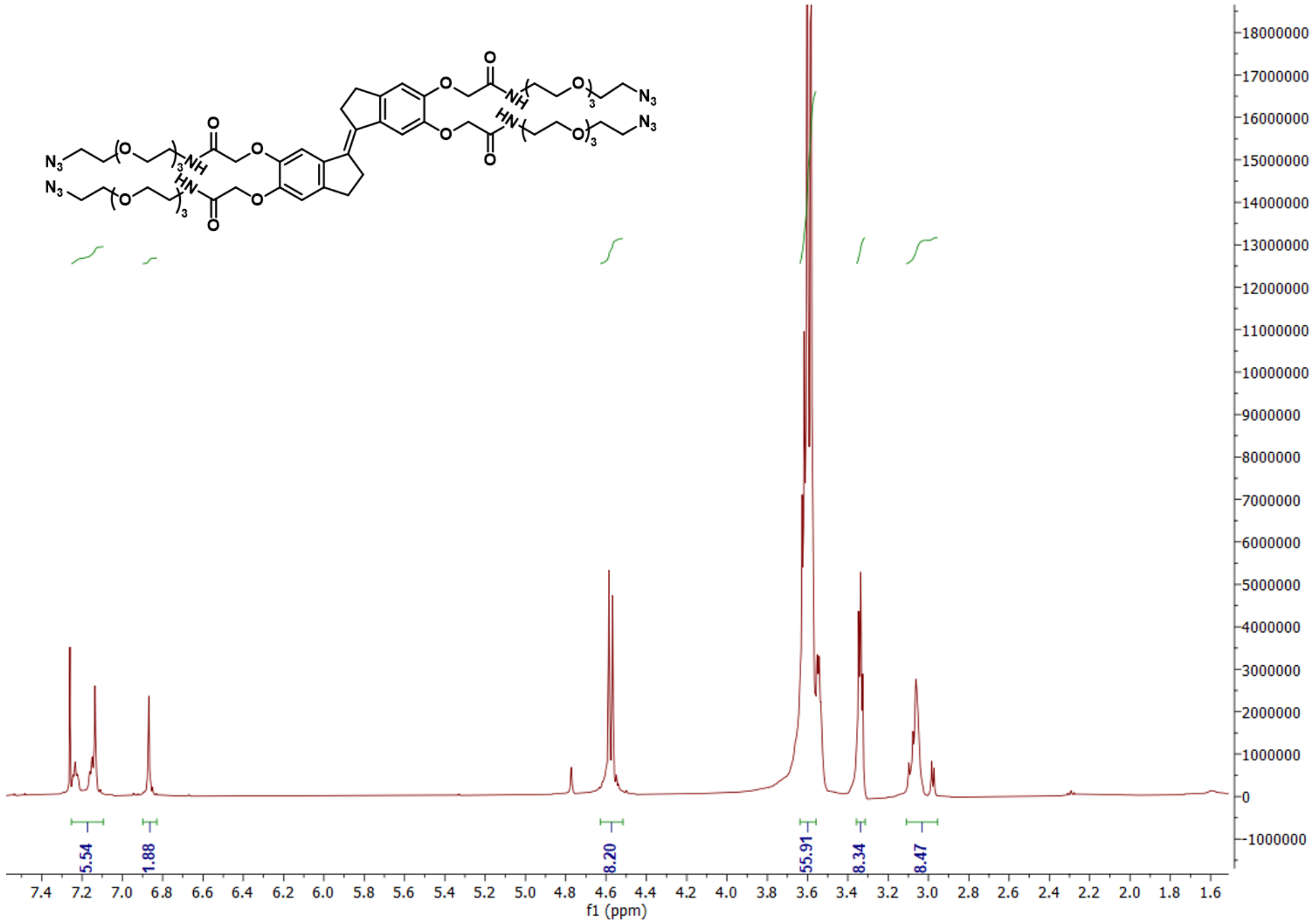

Figure S41. ^{1}H NMR spectrum of **Compound 22 (4AS)**

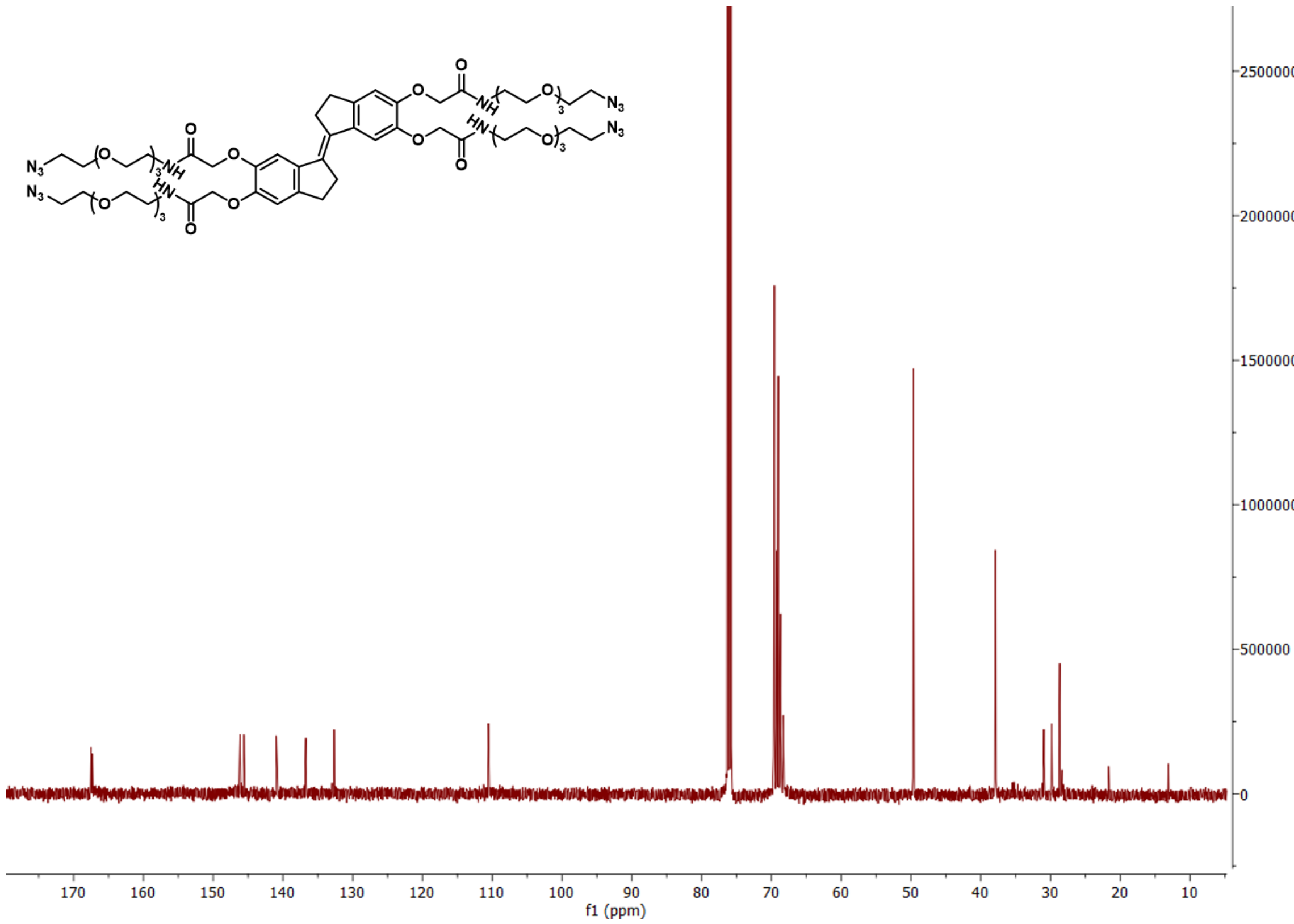

Figure S42.^{13}C NMR spectrum of **Compound 22 (4AS)**

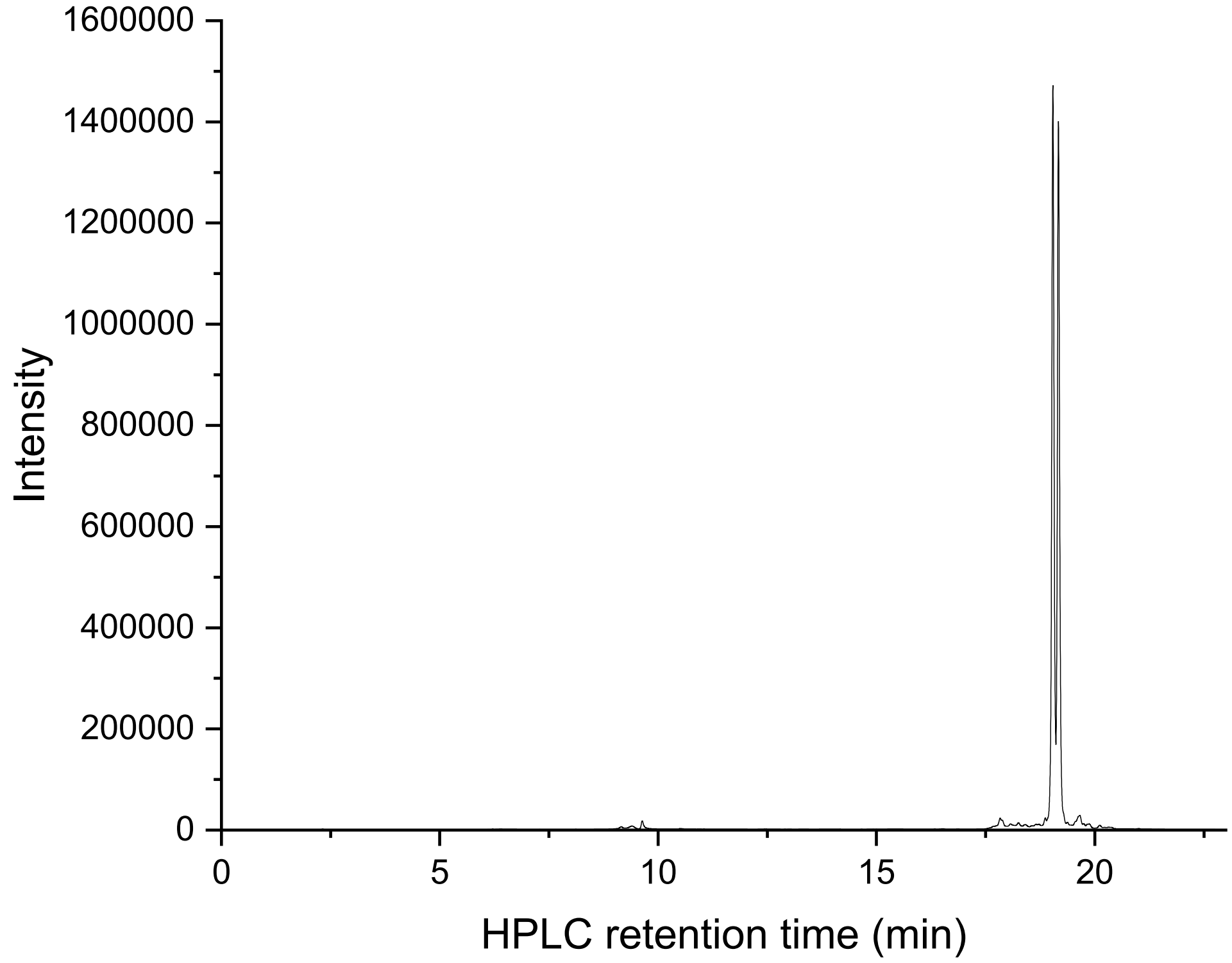

Figure S43. Reversed-phase HPLC (detected at 254 nm) of **Compound 14** (**2AM**)

Section XVII. Uncropped gel images

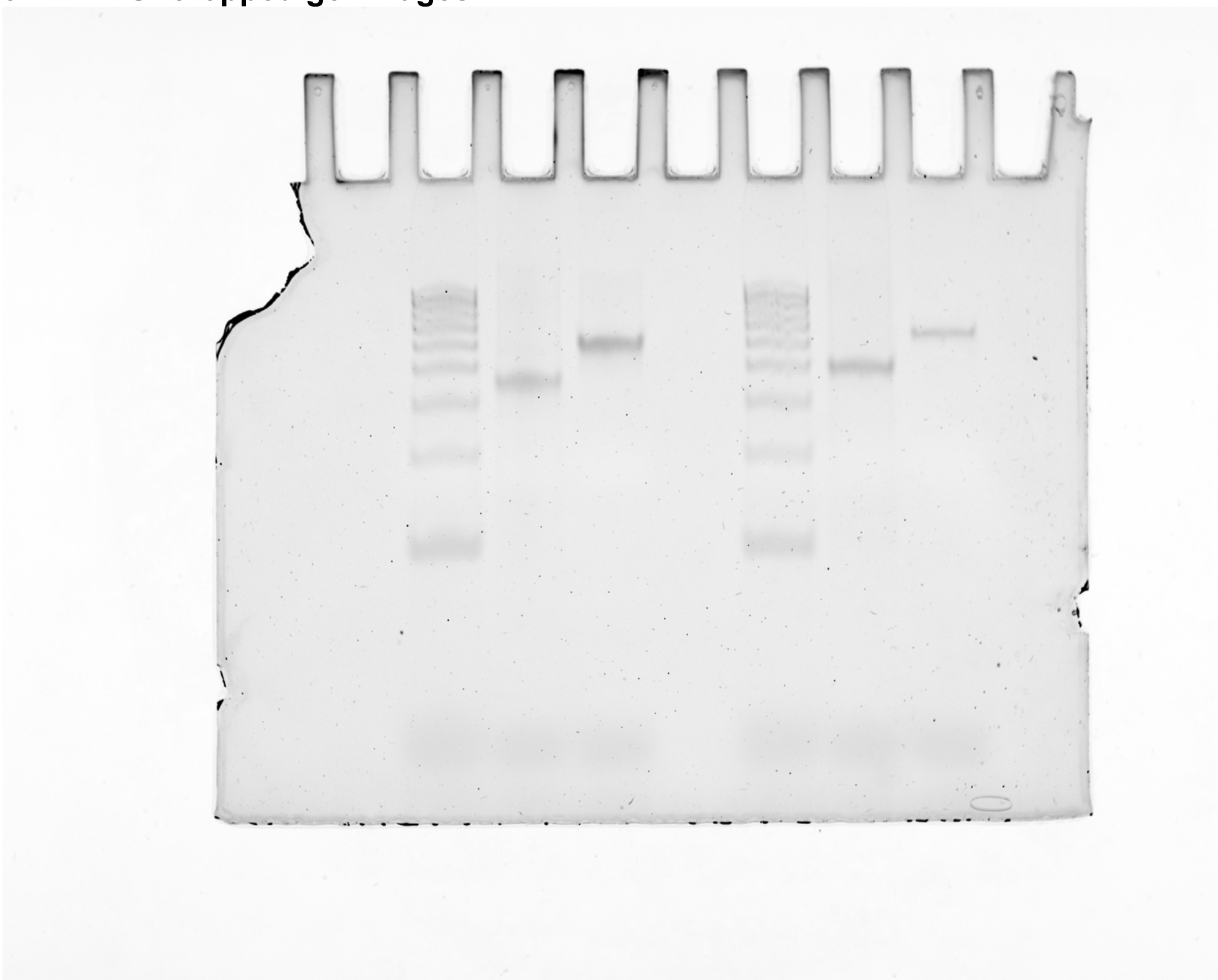

Figure S44. Uncropped image of the original gel, with the three rightmost lanes corresponding to those shown in Fig. 1f, and the three leftmost lanes corresponding to those shown in Fig. S17b.

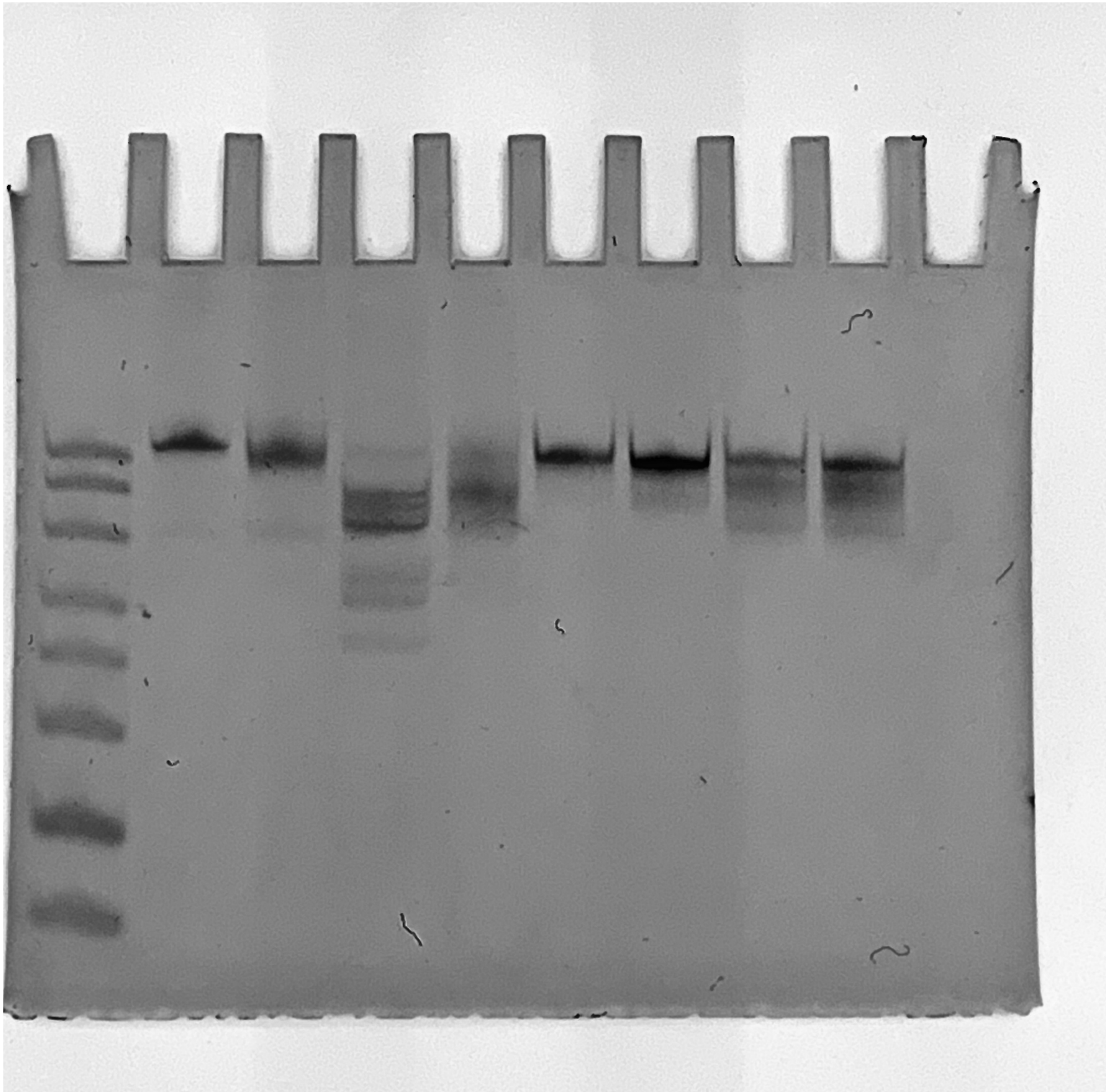

Figure S45. Uncropped image of the original gel, with the three leftmost lanes corresponding to those shown in Fig. 2a.

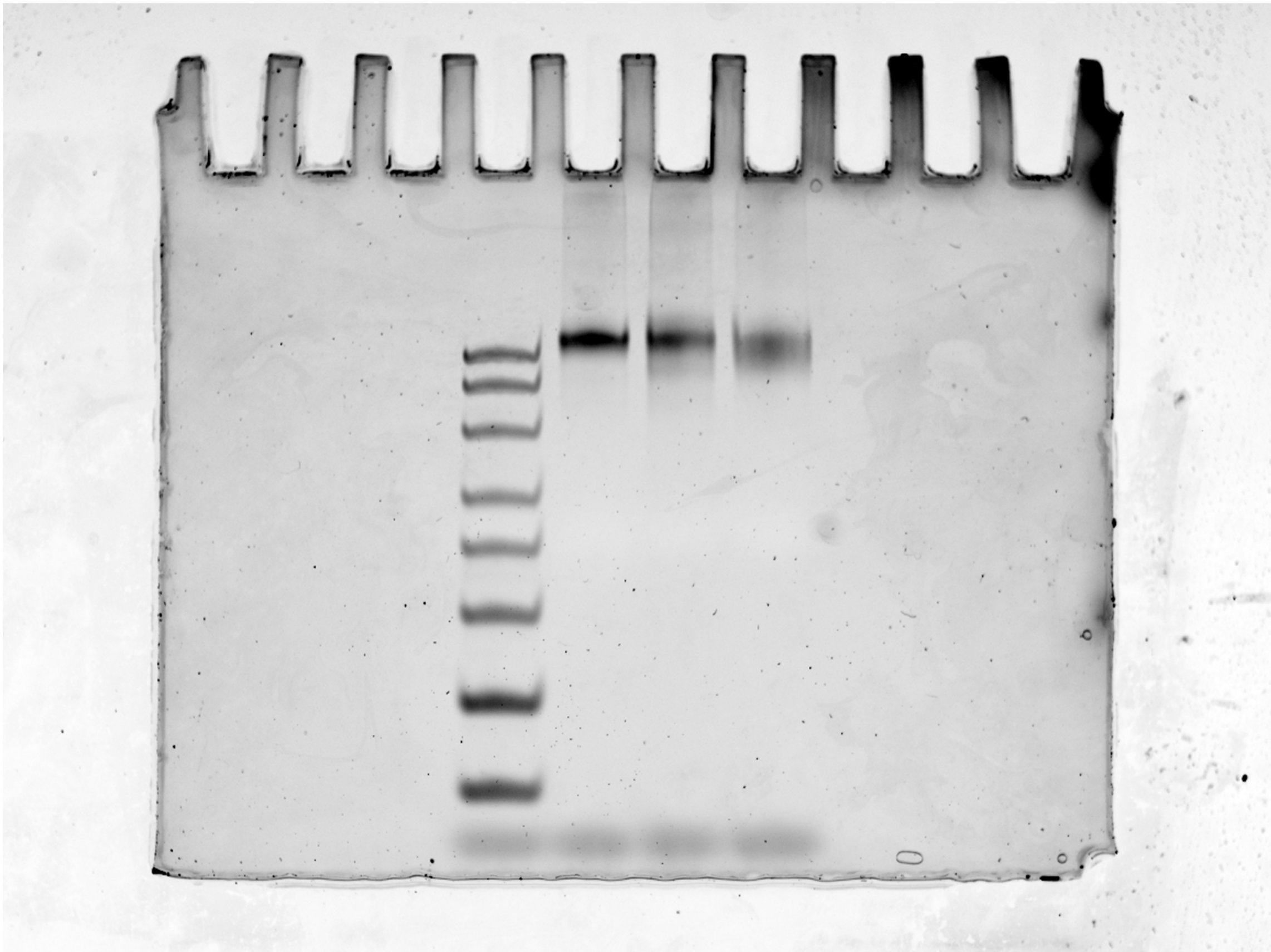

Figure S46. Uncropped image of the original gel shown in Fig. 2b.

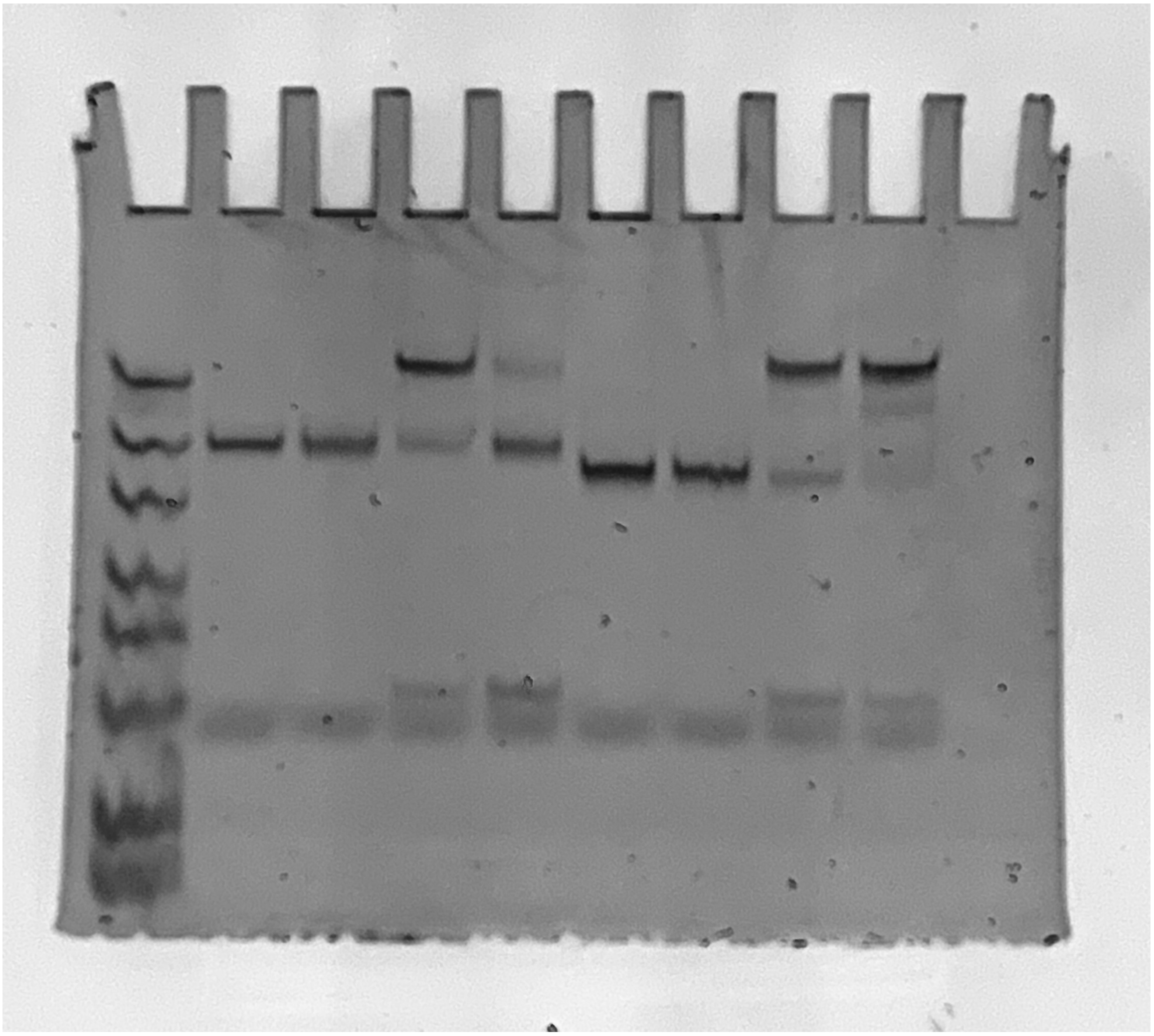

Figure S47. Uncropped image of the original gel, with the four leftmost lanes corresponding to those shown in Fig. 2g (top), and the four rightmost lanes corresponding to those shown in Fig. 3h.

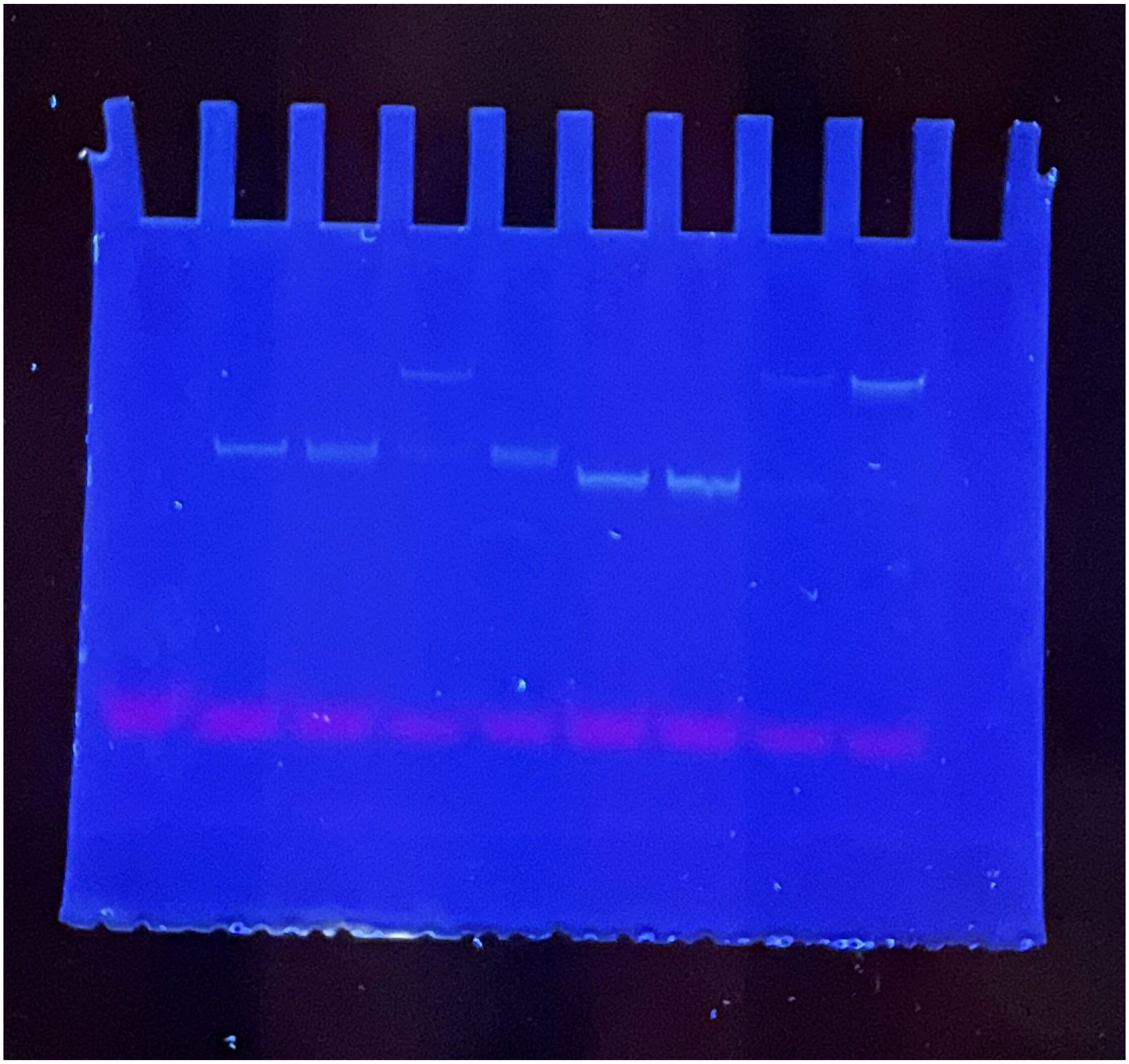

Figure S48. Uncropped image of the original gel, with the four leftmost lanes corresponding to those shown in Fig. 2g (bottom).

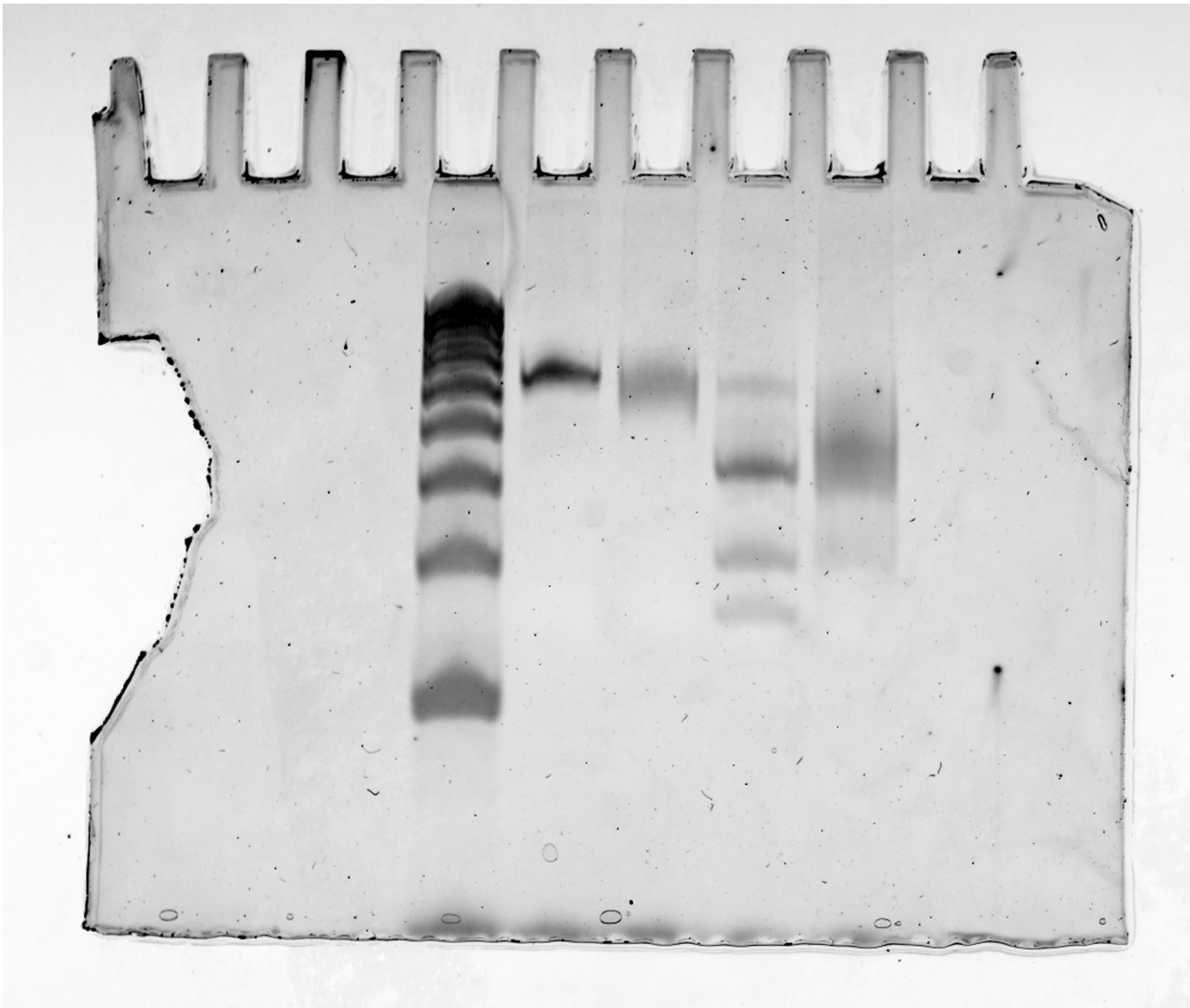

Figure S49. Uncropped image of the original gel shown in Fig. 2i.

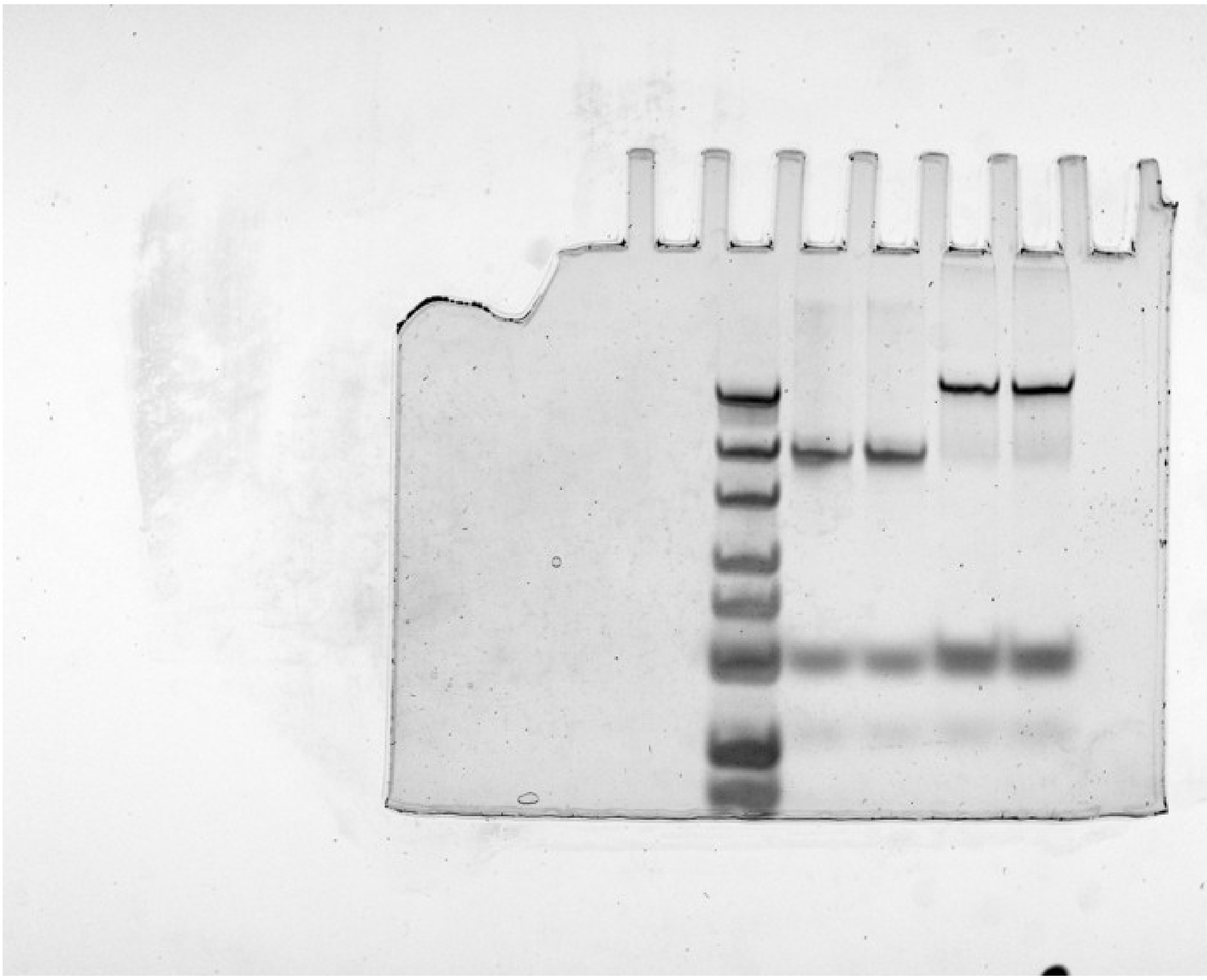

Figure S50. Uncropped image of the original gel shown in Fig. 3c.

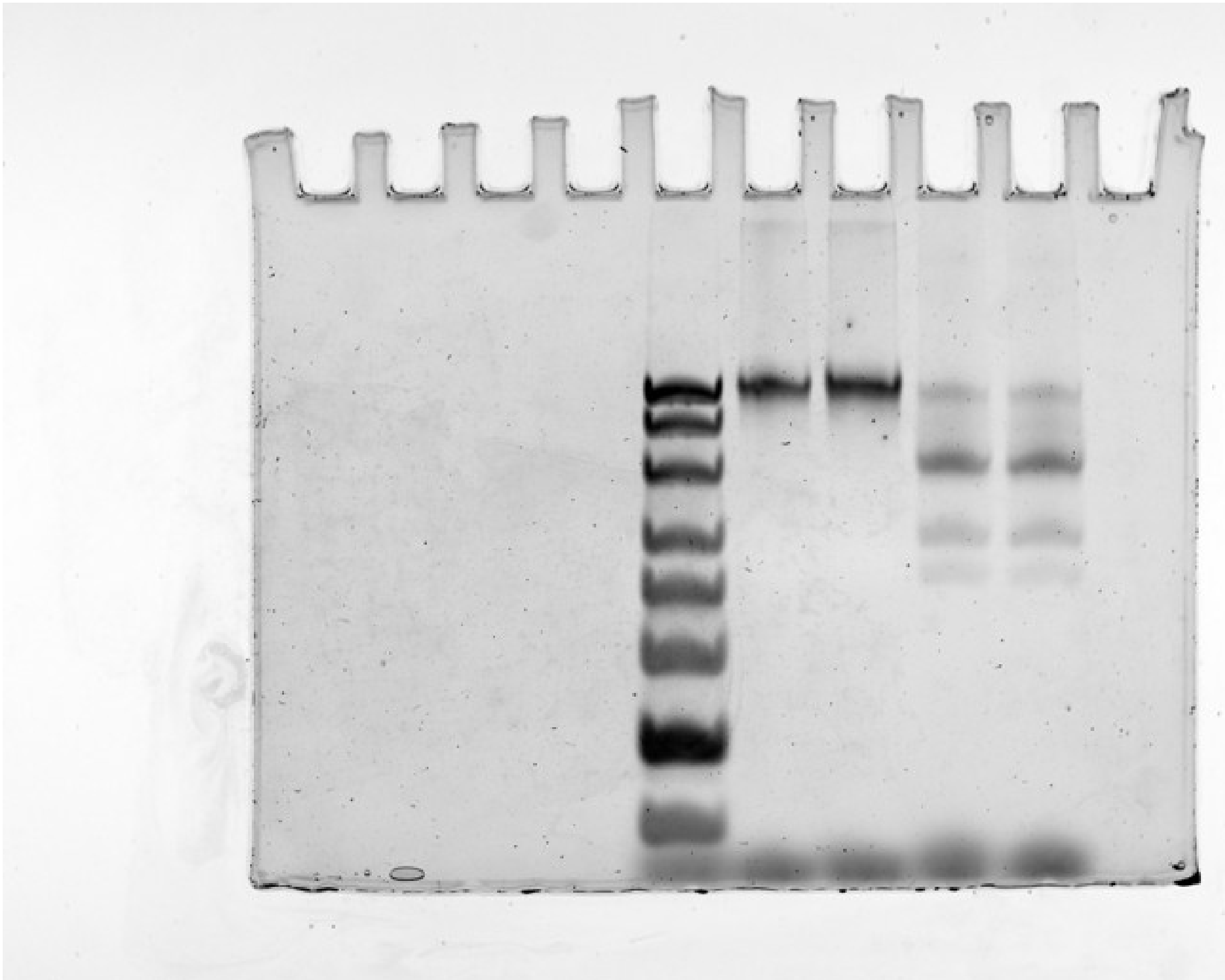

Figure S51. Uncropped image of the original gel shown in Fig. 3d.

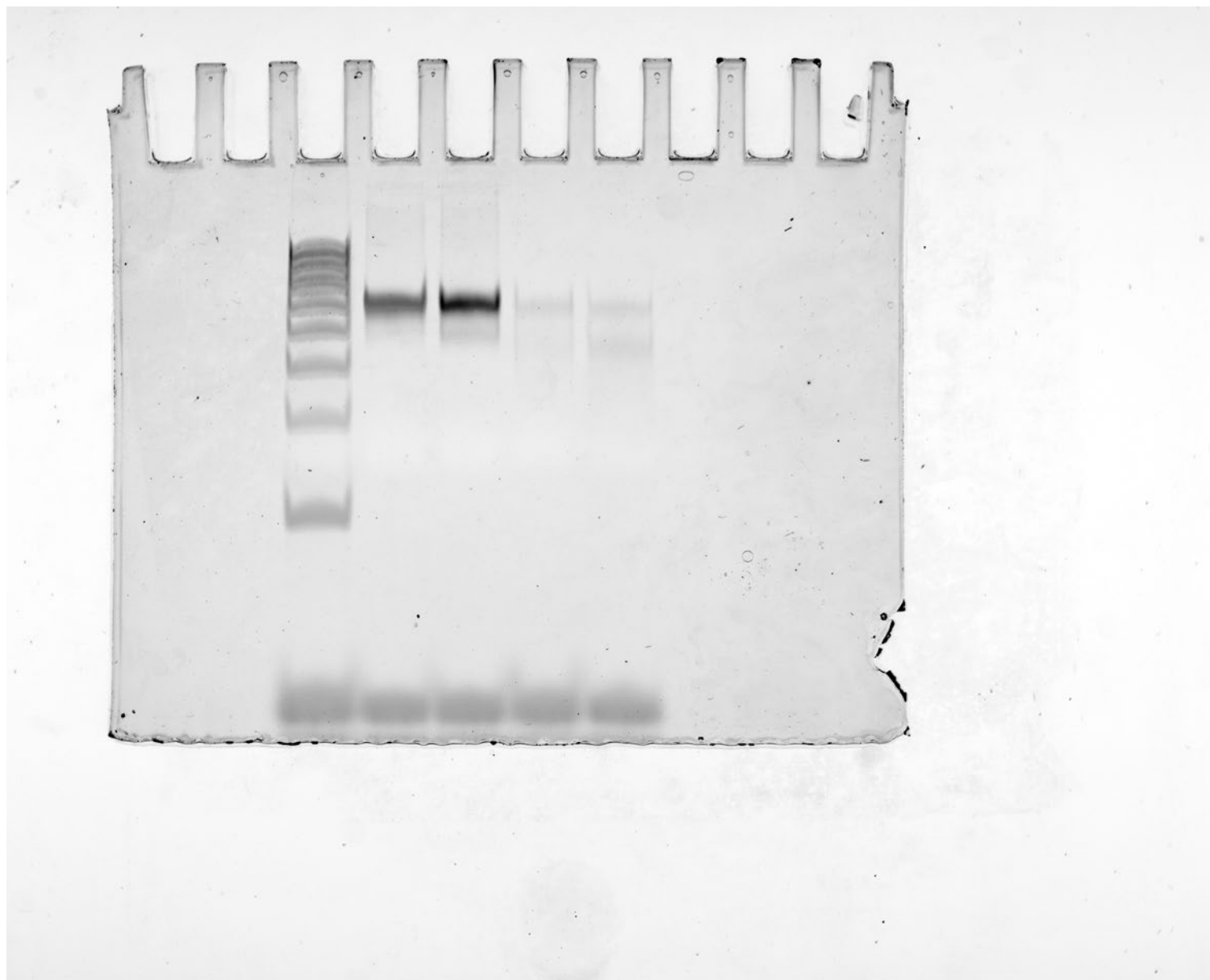

Figure S52. Uncropped image of the original gel shown in Fig. 3i.

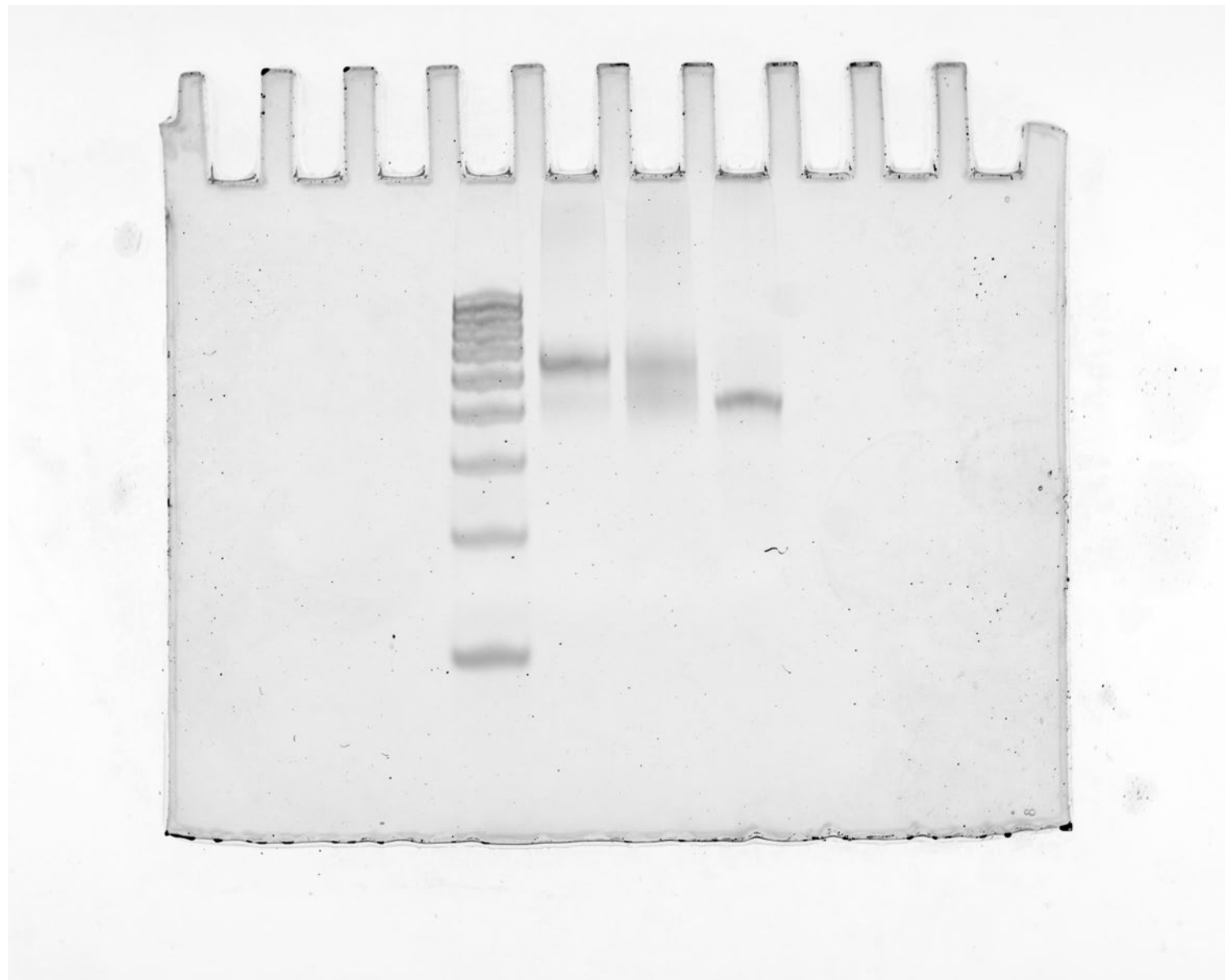

Figure S53. Uncropped image of the original gel shown in Fig. 5c.

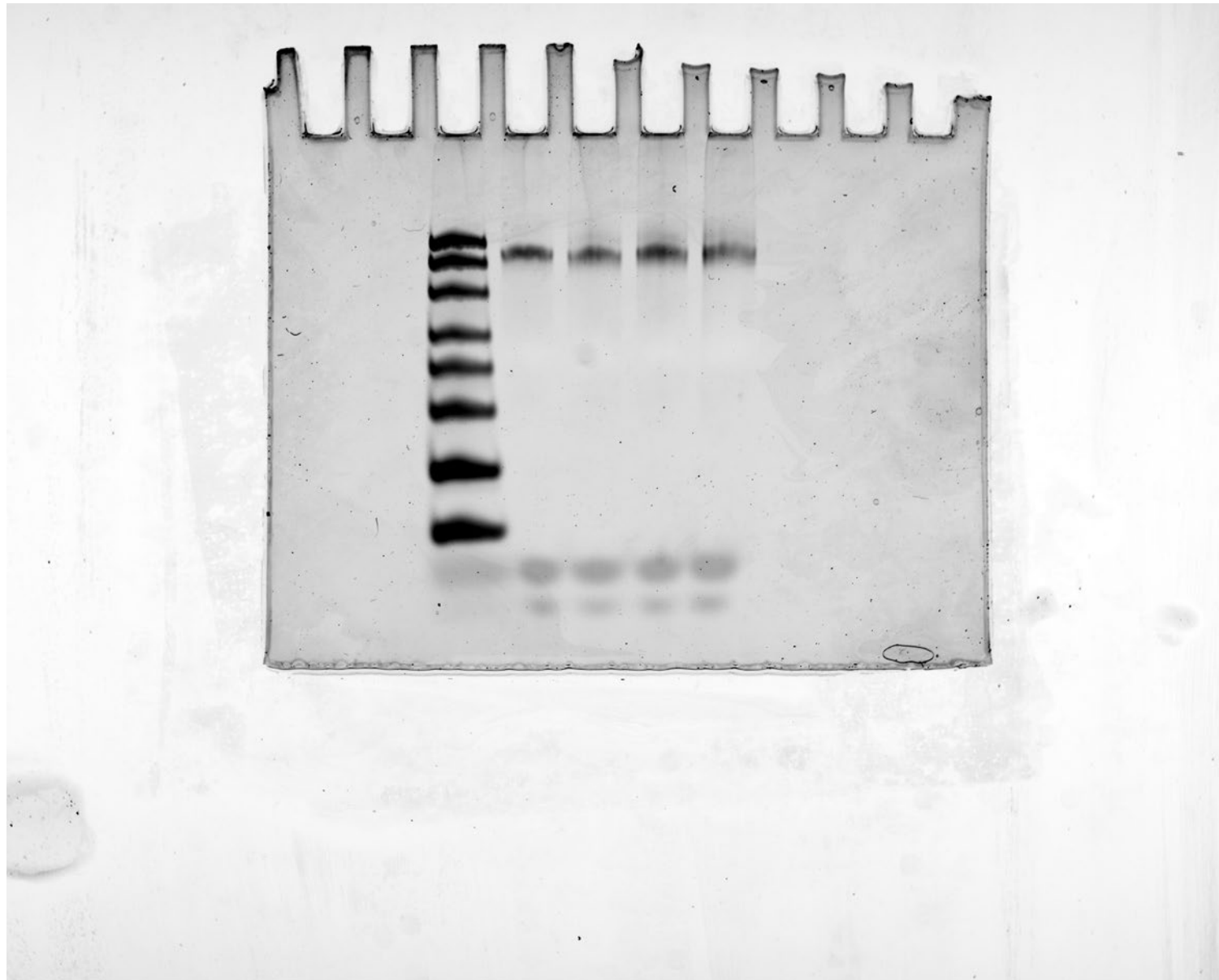

Figure S54. Uncropped image of the original gel shown in Fig. 5d.

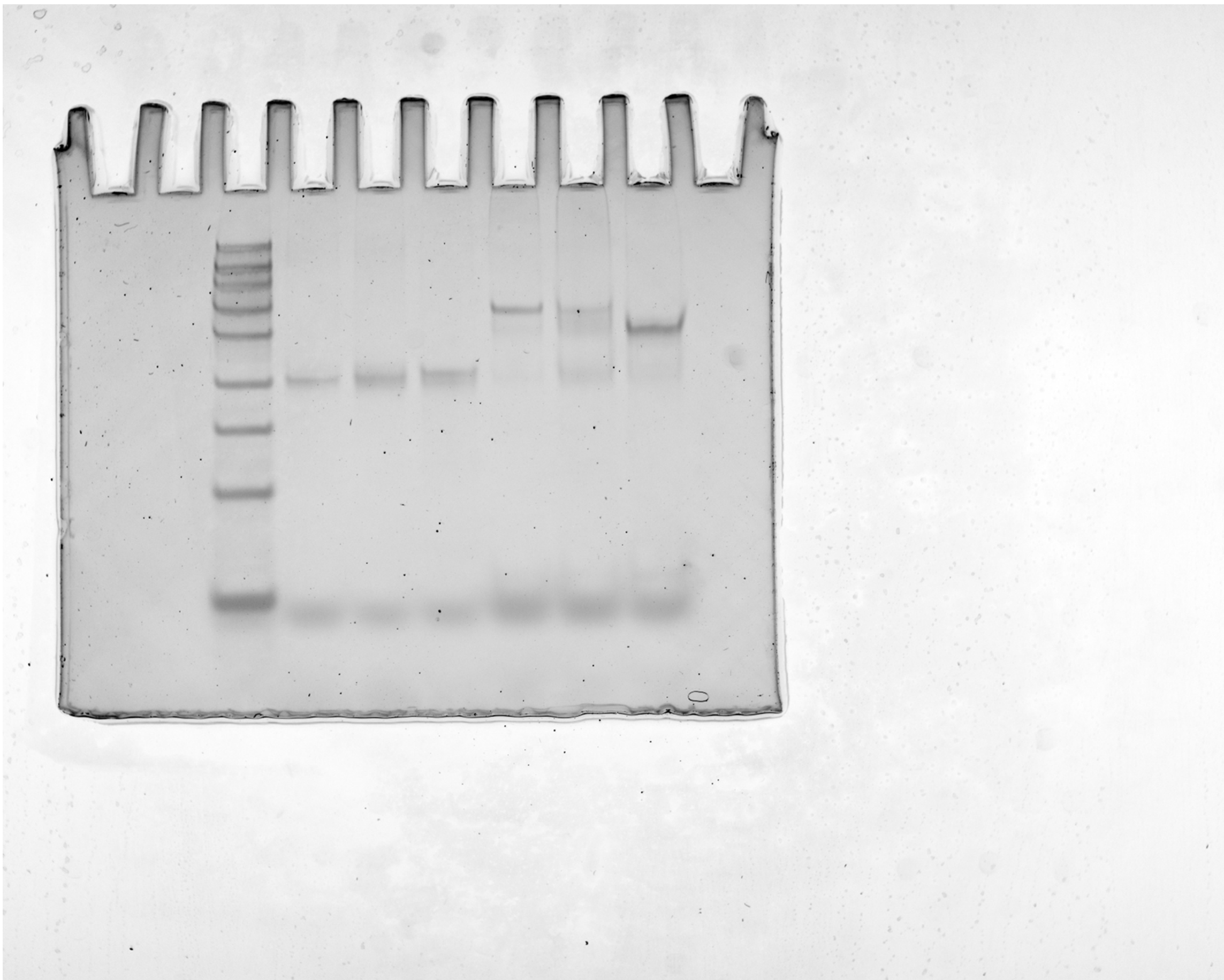

Figure S55. Uncropped image of the original gel shown in Fig. 5e.

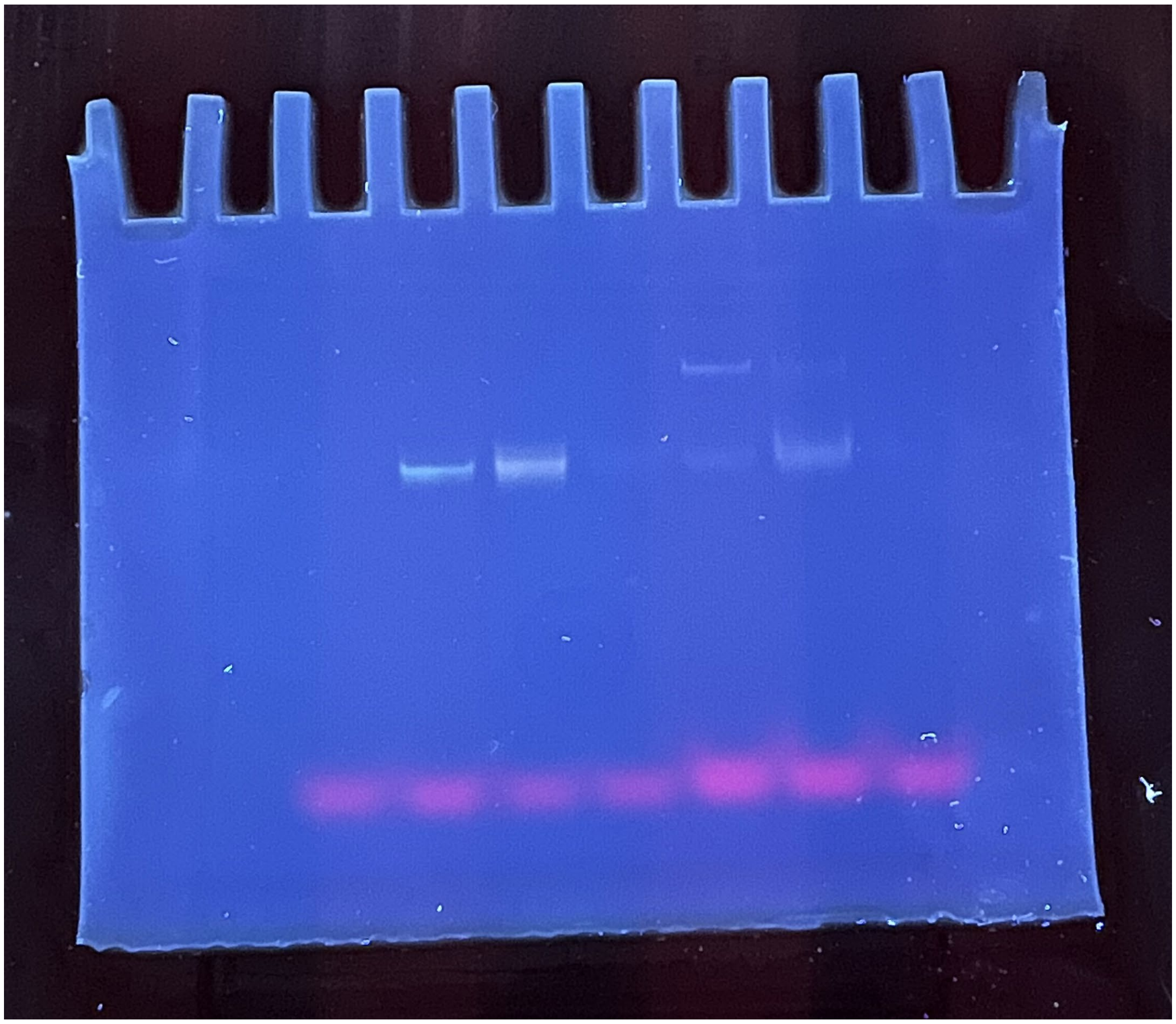

Figure S56. Uncropped image of the original gel shown in Fig. 5f.

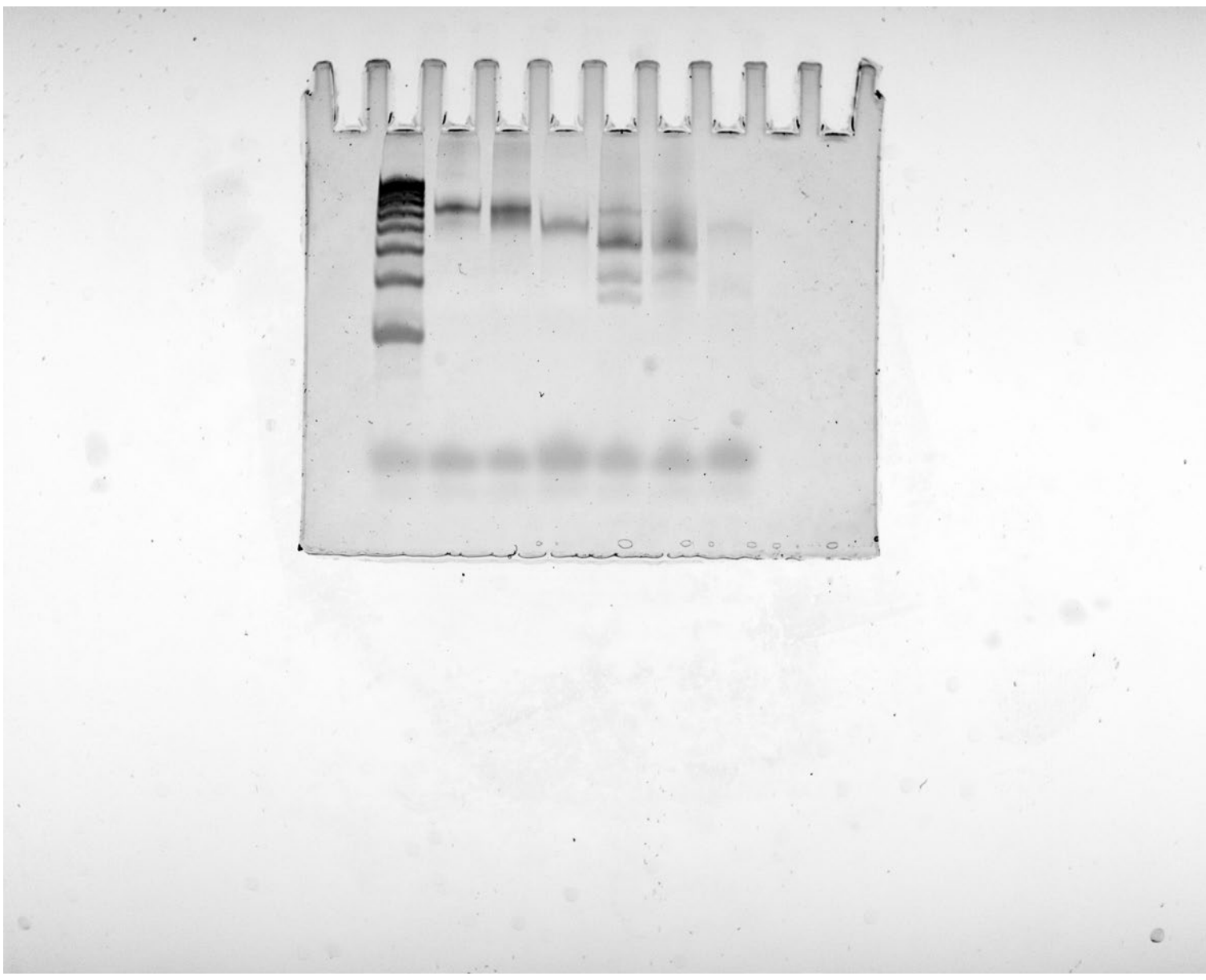

Figure S57. Uncropped image of the original gel shown in Fig. 5g.

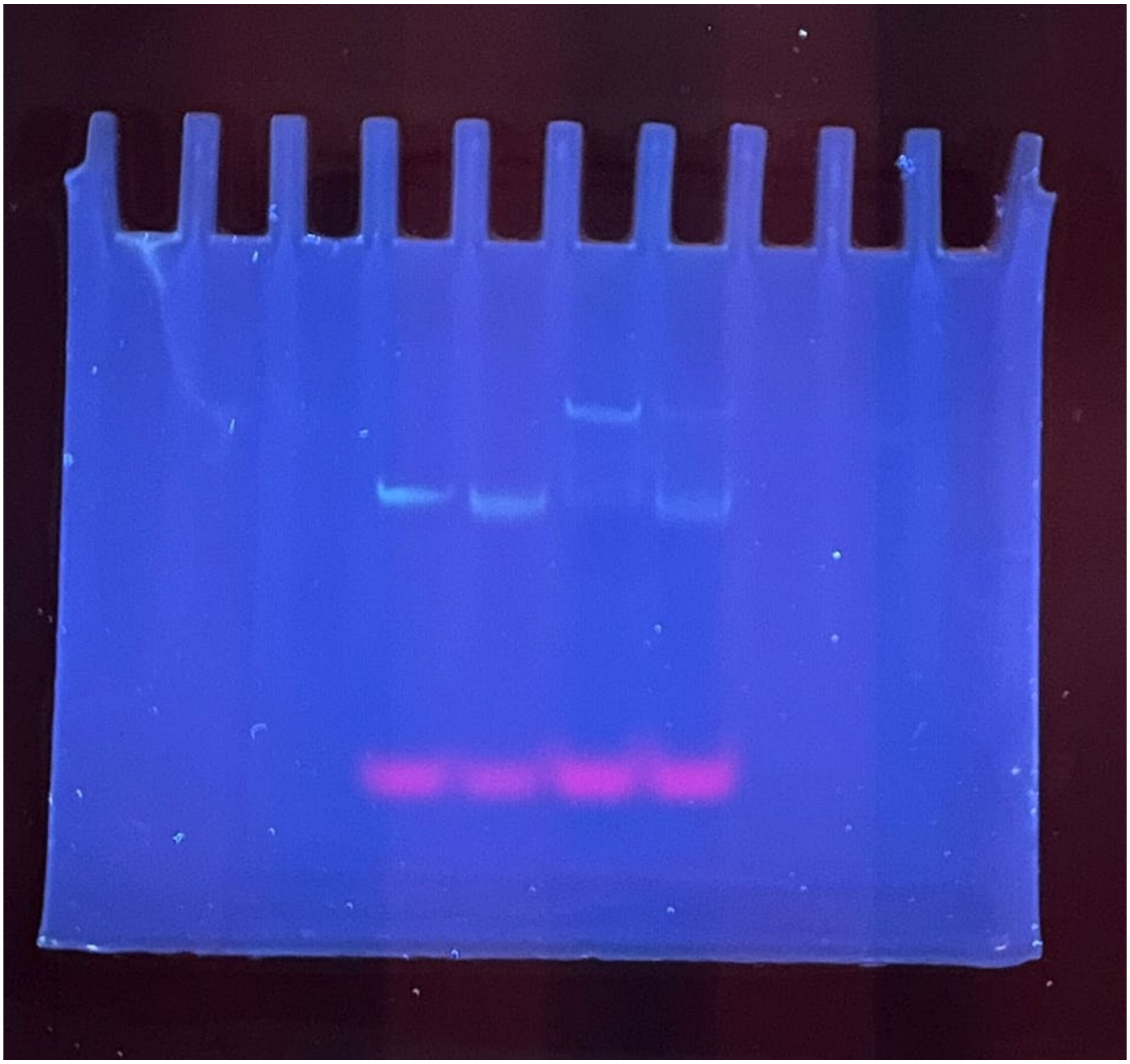

Figure S58. Uncropped image of the original gel shown in Fig. S6a.

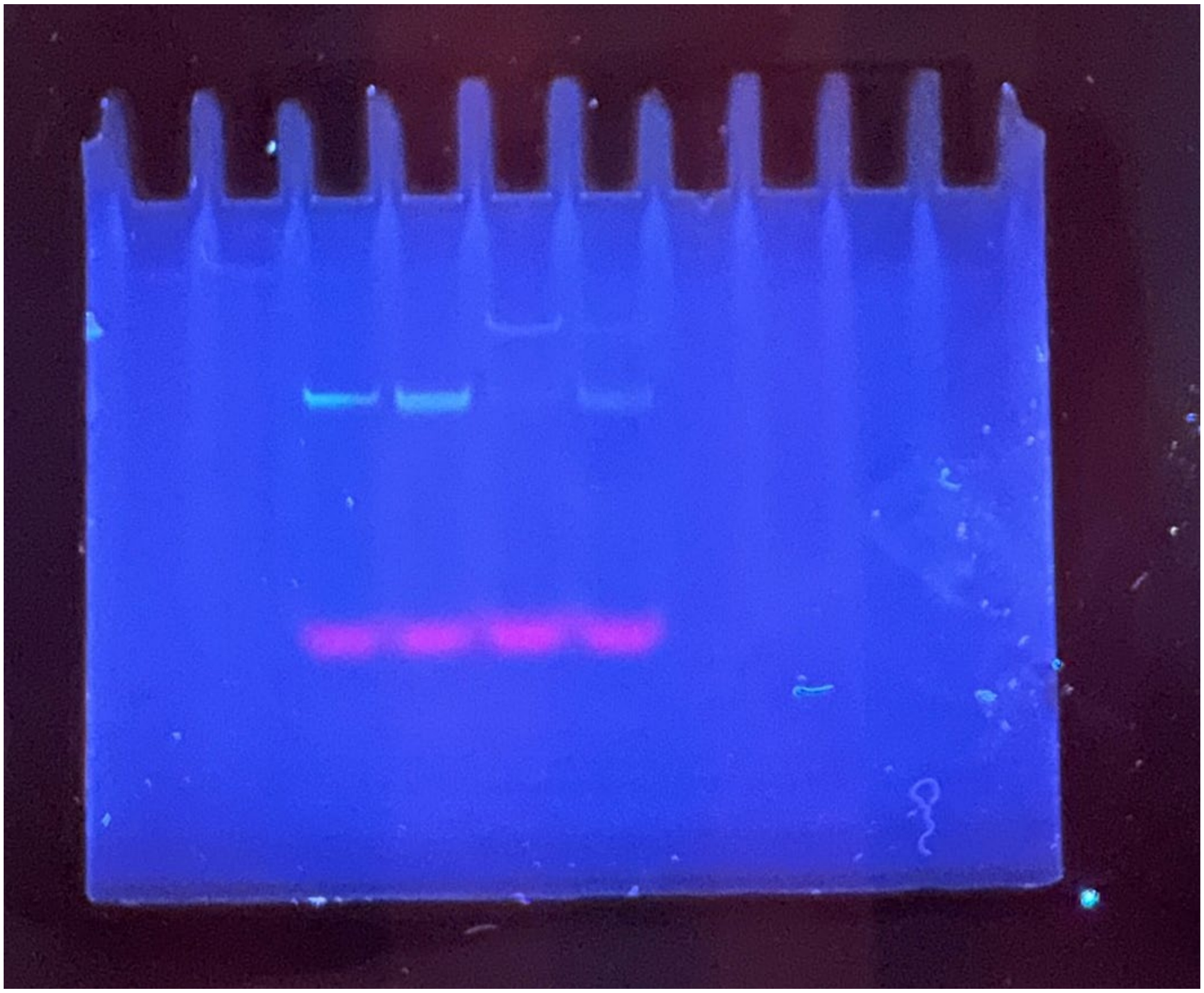

Figure S59. Uncropped image of the original gel shown in Fig. S6b.

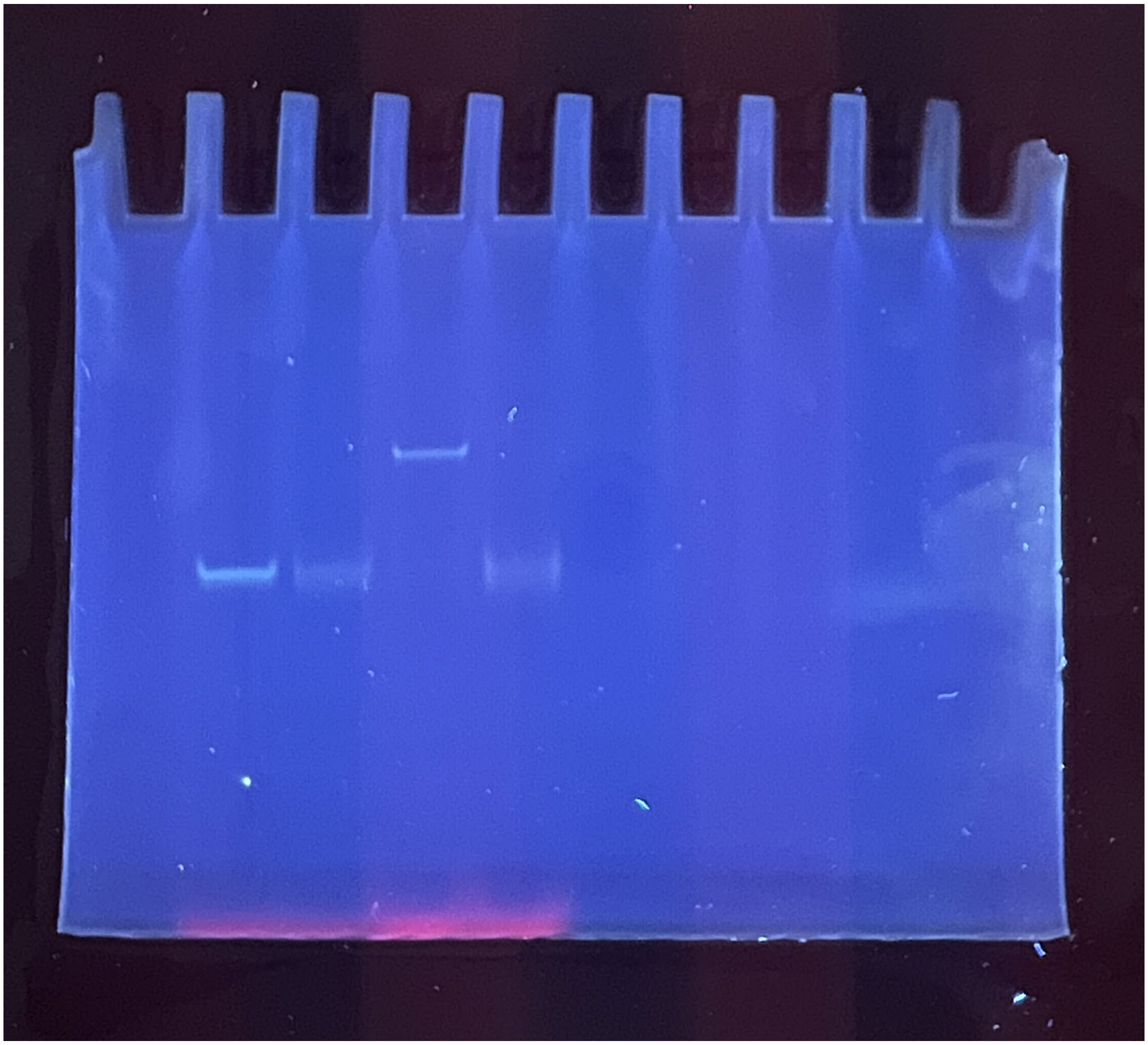

Figure S60. Uncropped image of the original gel shown in Fig. S6c.

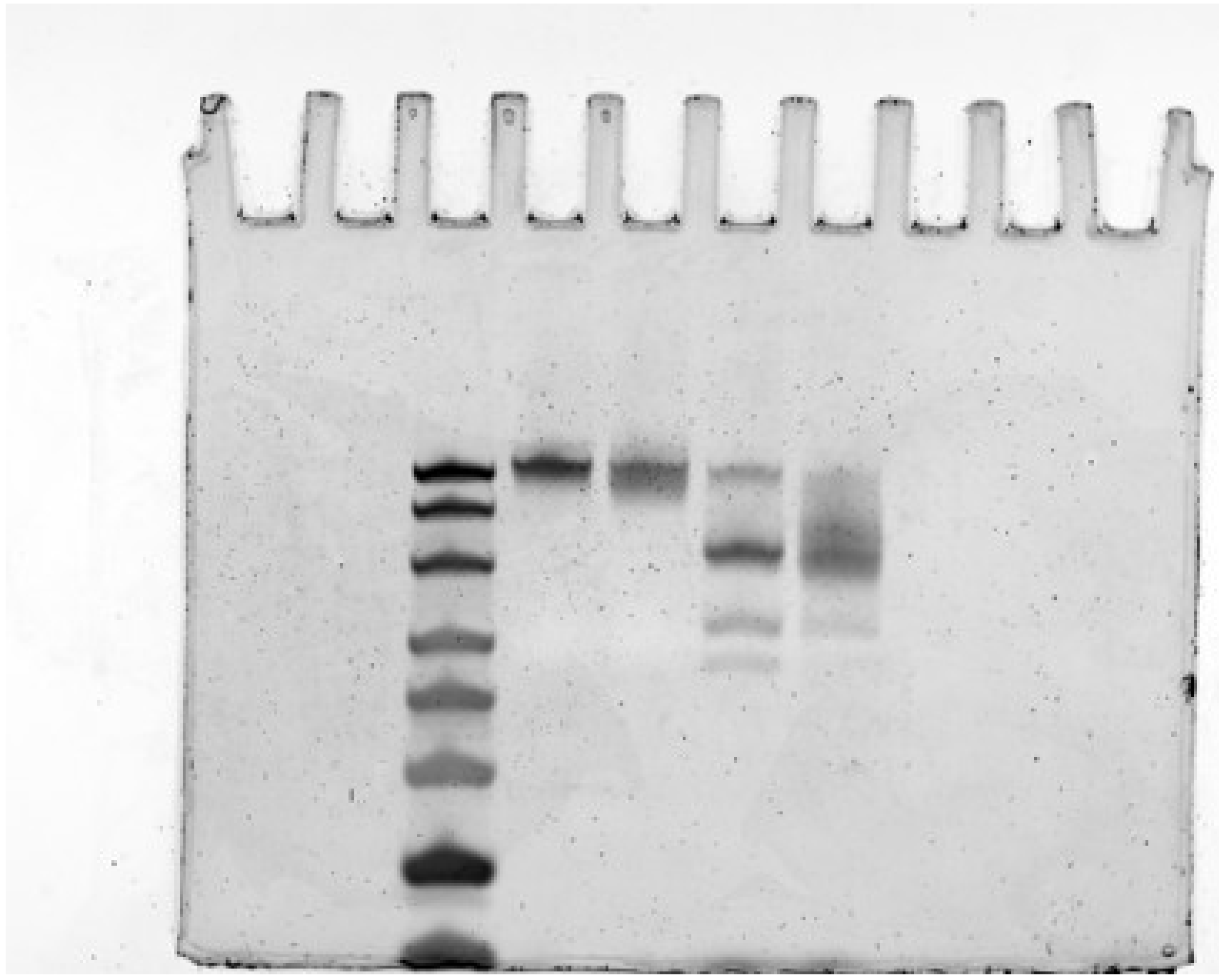

Figure S61. Uncropped image of the original gel shown in Fig. S6d.

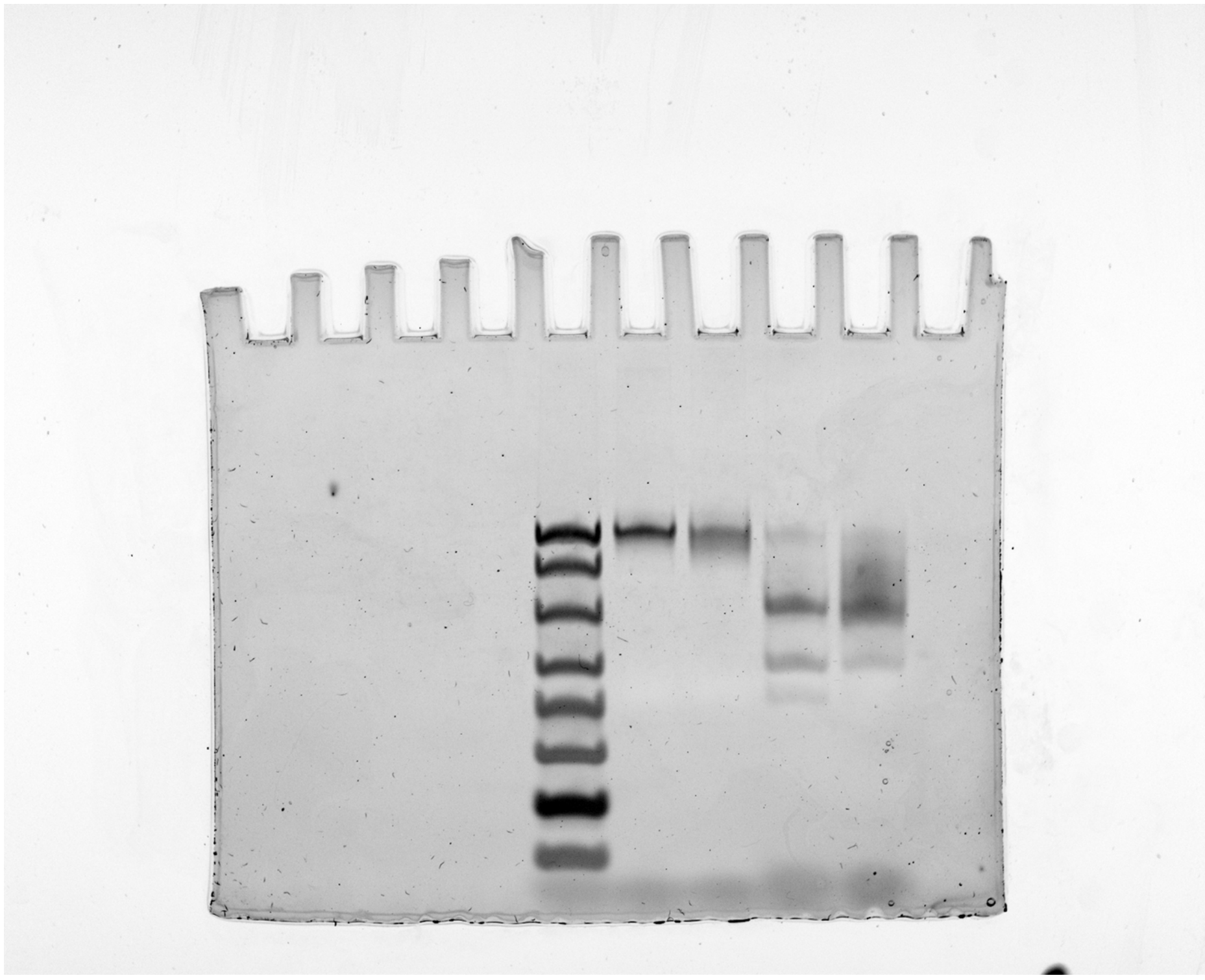

Figure S62. Uncropped image of the original gel shown in Fig. S6e.

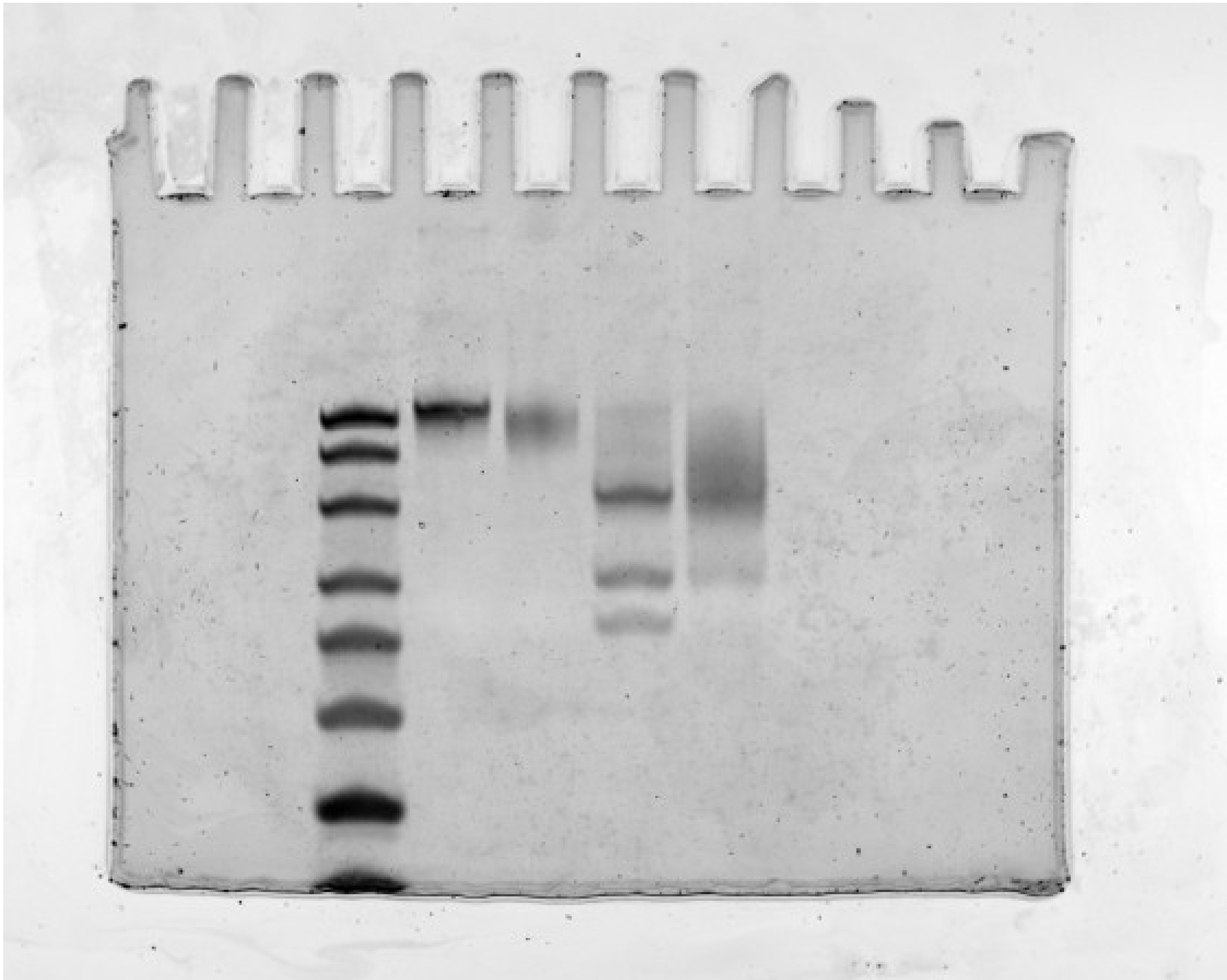

Figure S63. Uncropped image of the original gel shown in Fig. S6f.

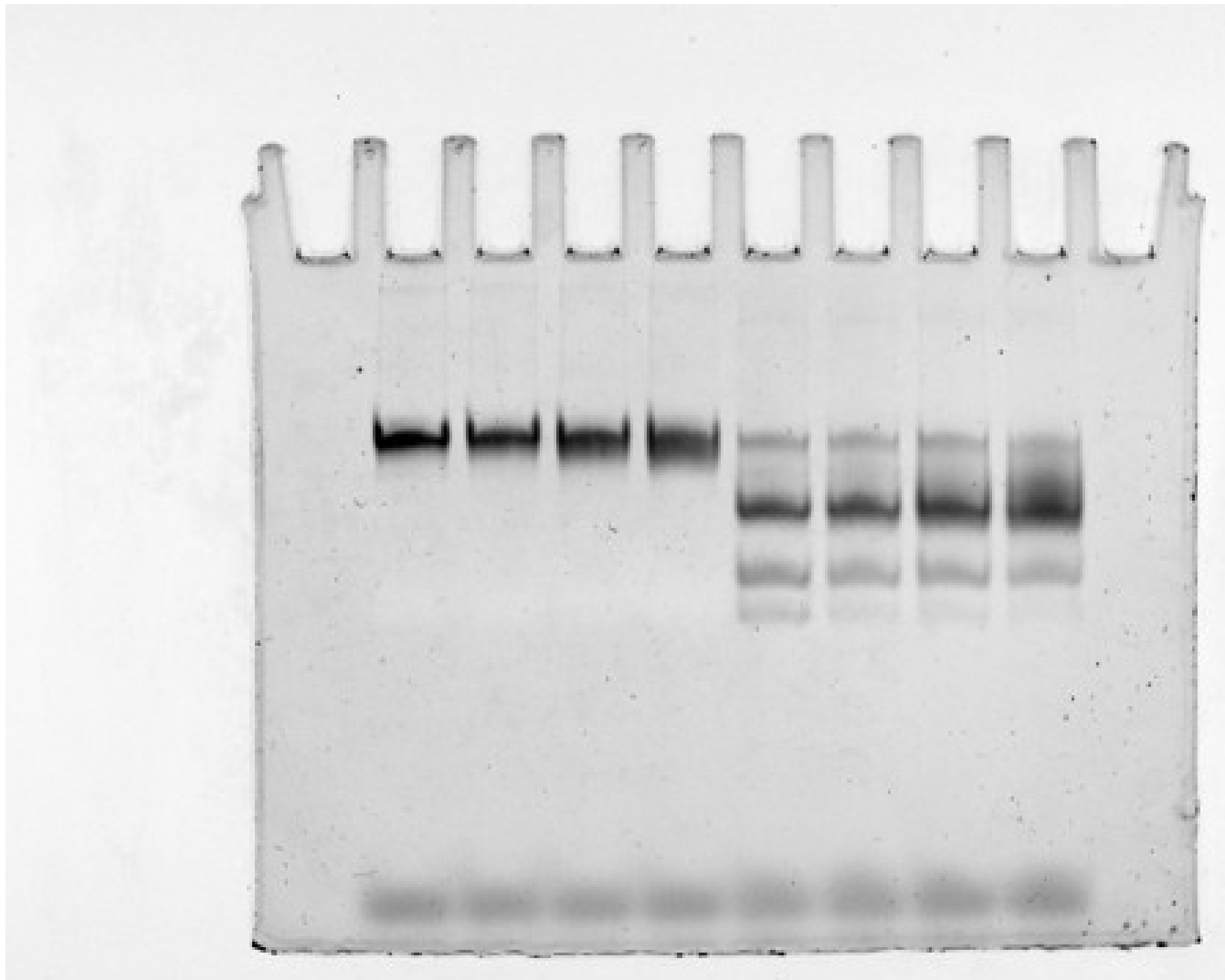

Figure S64. Uncropped image of the original gel shown in Fig. S9.

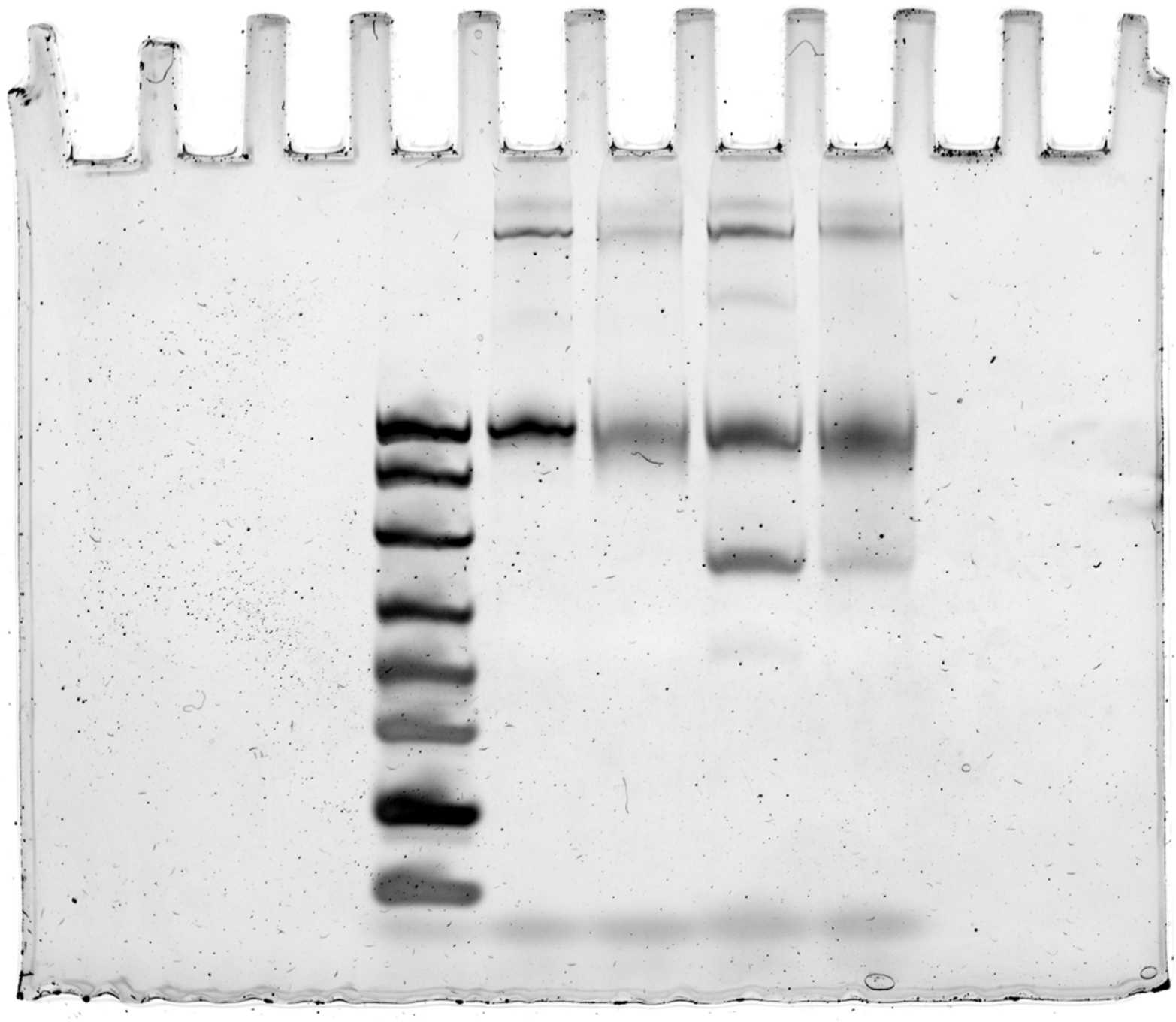

Figure S65. Uncropped image of the original gel shown in Fig. S12.

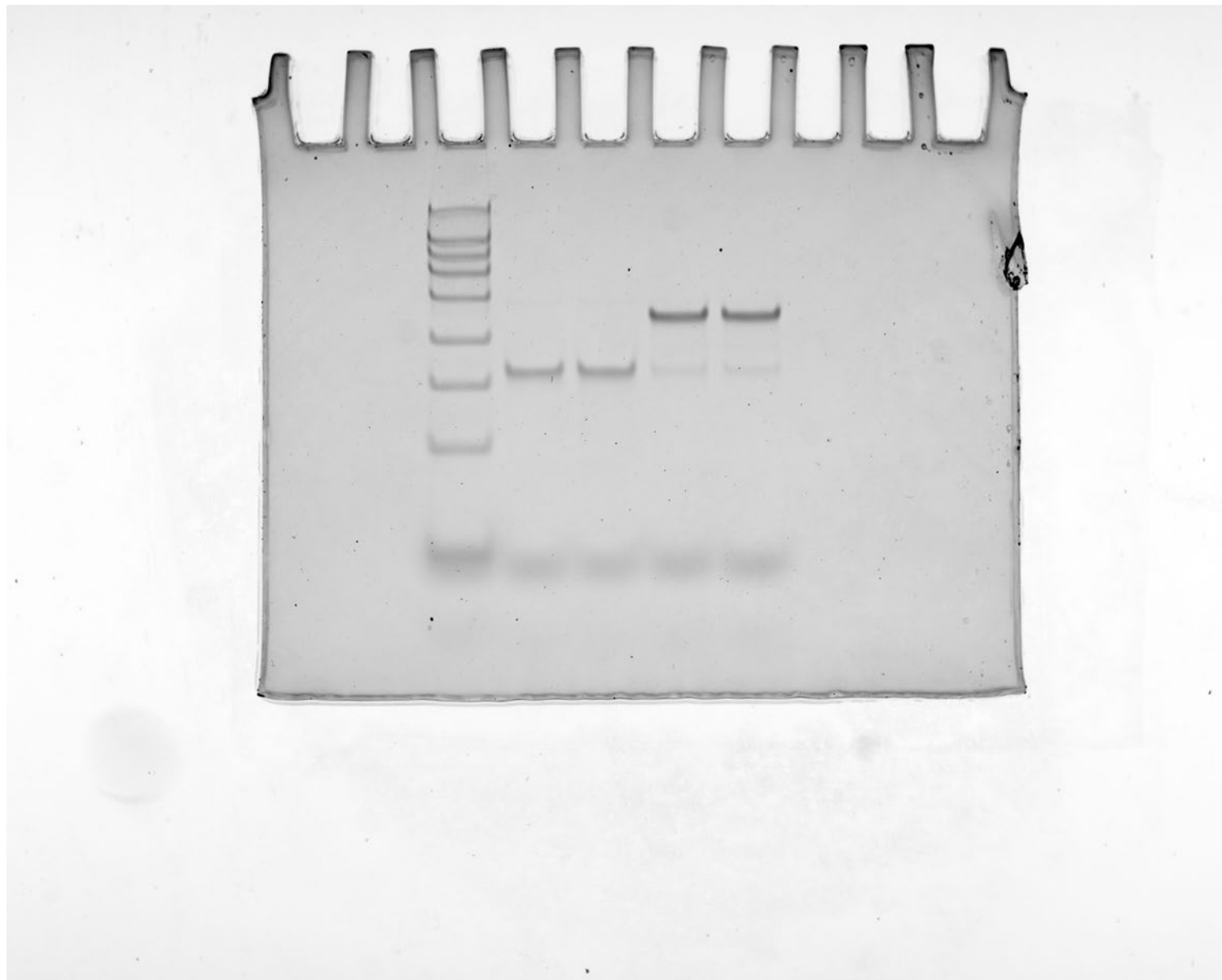

Figure S66. Uncropped image of the original gel shown in Fig. S16b.

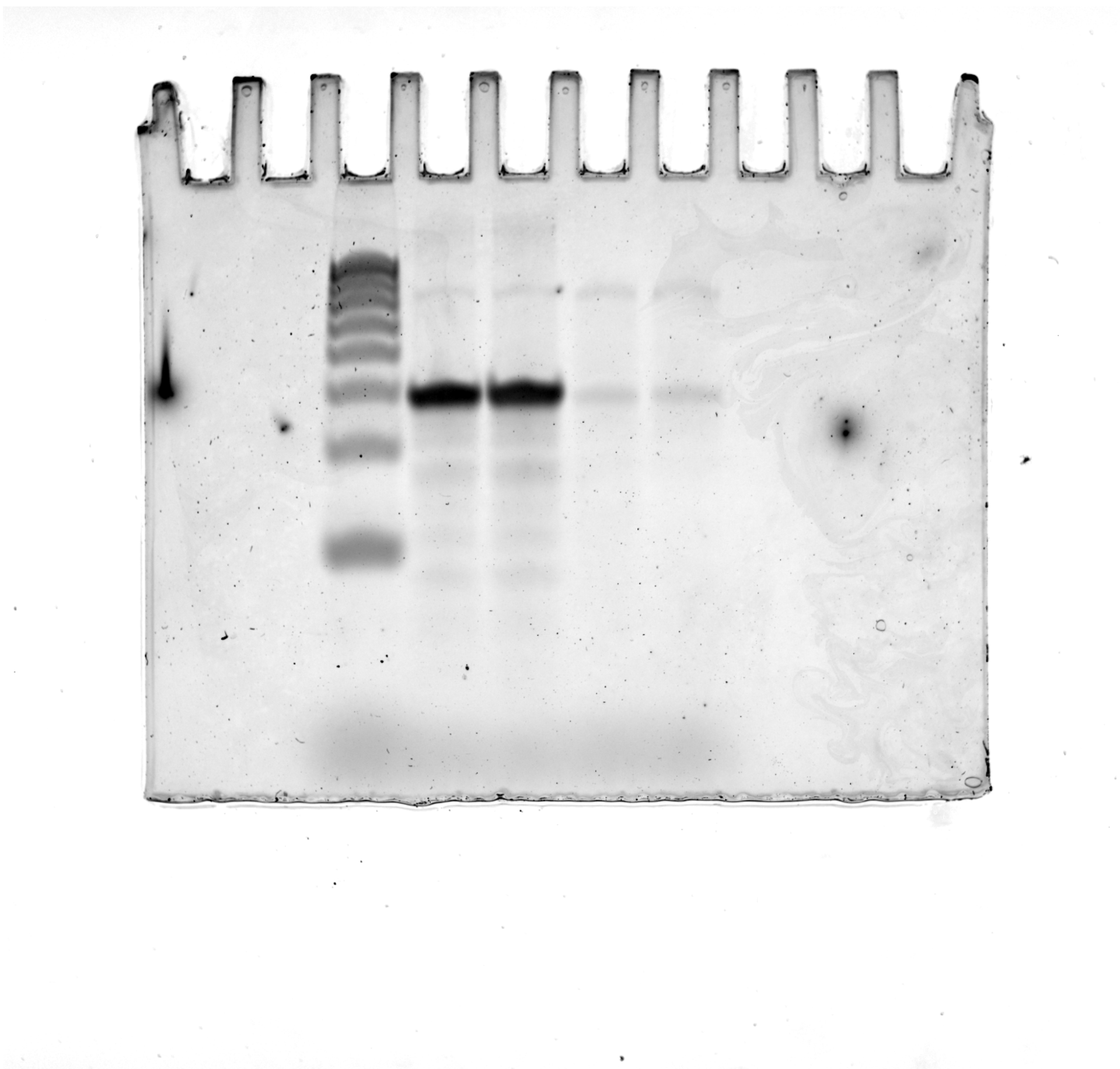

Figure S67. Uncropped image of the original gel shown in Fig. S16c.

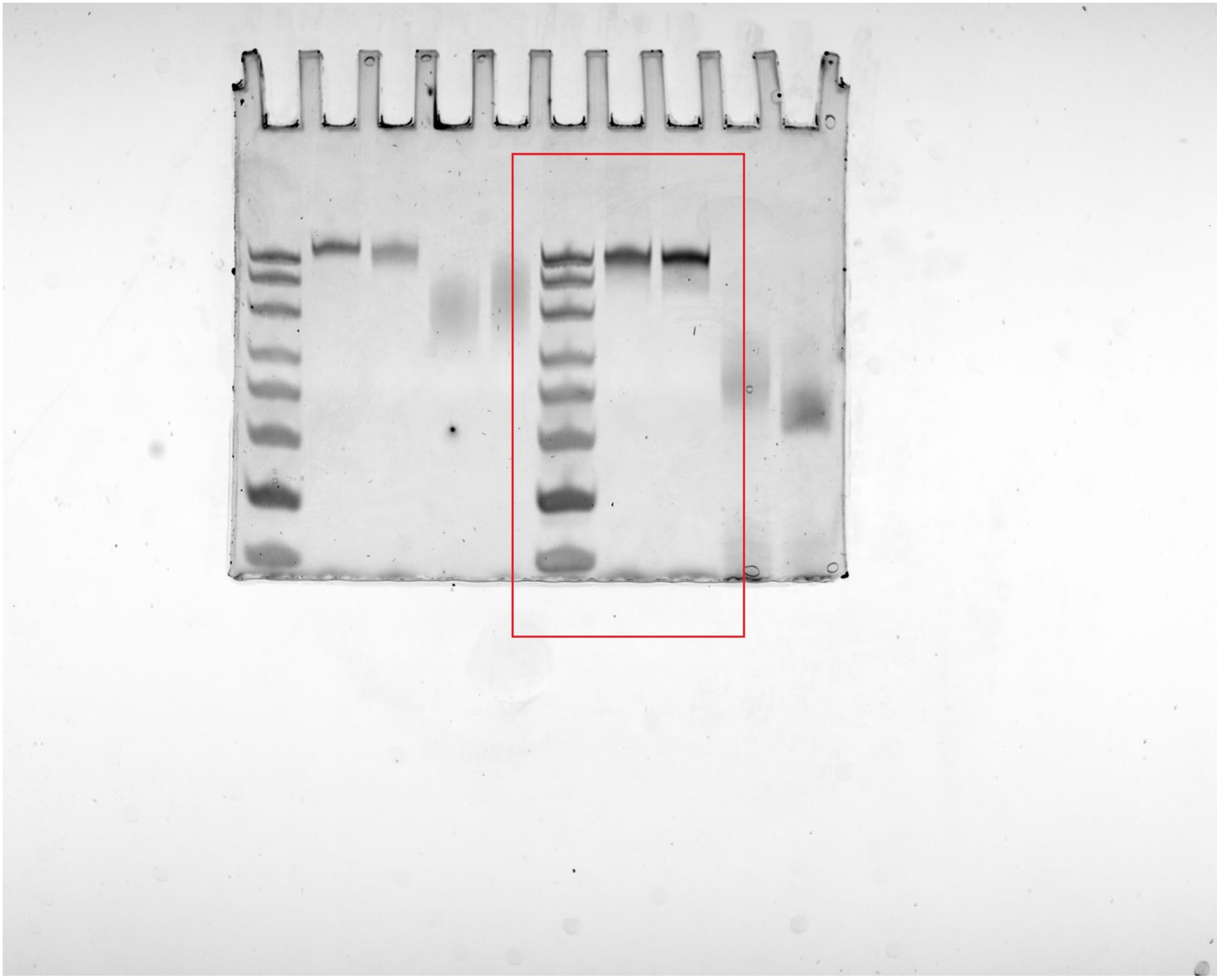

Figure S68. Uncropped image of the original gel shown in Fig. S18b.

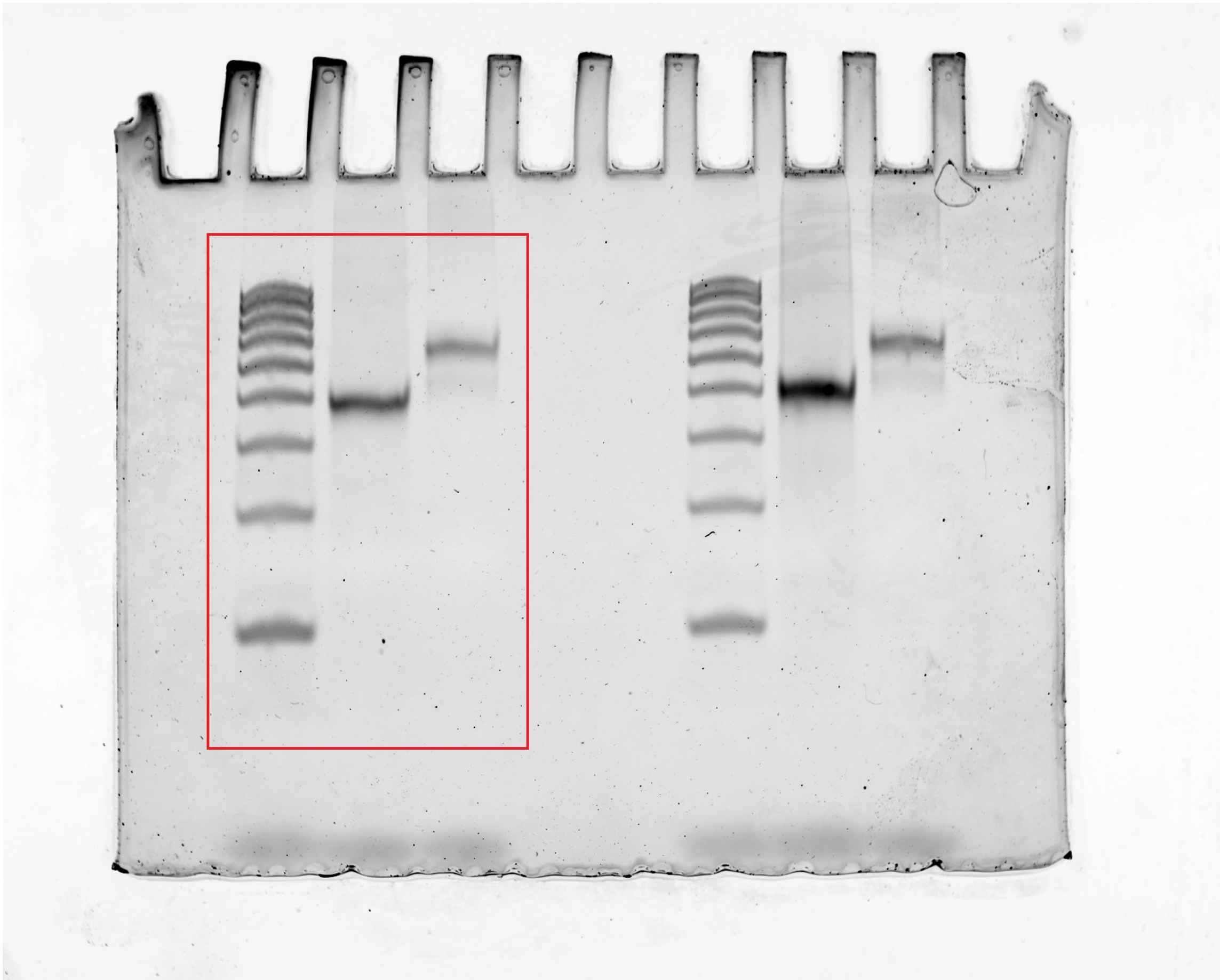

Figure S69. Uncropped image of the original gel shown in Fig. S23b.

SI References

[1] L. Yang, E. Lazo, J. Byrnes, S. Chodankar, S. Antonelli, M. Rakitin, "Tools for Supporting Solution Scattering During the COVID-19 Pandemic" *J. Synchrotron Radiat.* **28**, (2021): 1237-1244.

[2] J. Hopkins, R. Gillilan, S. Skou, "BioXTAS RAW 2.0: The Latest in Saxs Data Analysis" *Acta Crystallogr. A* **76**, (2020): A27.

[3] J. B. Hopkins, R. E. Gillilan, S. Skou, "BioXTAS RAW: A Free Open-Source Program for Small-Angle X-Ray Scattering Data Reduction" *Acta Crystallogr. A* **74**, (2018): a219.

[4] K. Manalastas-Cantos, P. V. Konarev, N. R. Hajizadeh, A. G. Kikhney, M. V. Petoukhov, D. S. Molodenskiy, A. Panjkovich, H. D. Mertens, A. Gruzinov, C. Borges, "ATSAS 3.0: expanded functionality and new tools for small-angle scattering data analysis" *J. Appl. Crystallogr.* **54**, (2021): 343-355.

[5] X. J. Lu, W. K. Olson, "3DNA: A Software Package for the Analysis, Rebuilding and Visualization of Three-Dimensional Nucleic Acid Structures" *Nucleic Acids Res.* **31**, (2003): 5108-5121.

[6] K. Luger, A. W. Mäder, R. K. Richmond, D. F. Sargent, T. J. Richmond, "Crystal structure of the nucleosome core particle at 2.8 Å resolution" *Nature* **389**, (1997): 251-260.

[7] H. W. Horn, W. C. Swope, J. W. Pitera, J. D. Madura, T. J. Dick, G. L. Hura, T. Head-Gordon, "Development of an improved four-site water model for biomolecular simulations: TIP4P-Ew" *J. Chem. Phys.* **120**, (2004): 9665-9678.

[8] Q. Li, J. T. Foy, J.-R. Colard-Itté, A. Goujon, D. Dattler, G. Fuks, E. Moulin, N. Giuseppone, "Gram scale synthesis of functionalized and optically pure Feringa's motors" *Tetrahedron* **73**, (2017): 4874-4882.

[9] M. Klok, N. Boyle, M. T. Pryce, A. Meetsma, W. R. Browne, B. L. Feringa, "MHz unidirectional rotation of molecular rotary motors" *J. Am. Chem. Soc.* **130**, (2008): 10484-10485.

[10] M. Banck, F. Bresciani, J. Bréfort, A. Clark, J. Corkery, V. Favre-Nicolin, F. Fontaine, M. Gillies, R. Gillilan, B. Goldman, T. Hassinen, B. Herger, G. Hutchison, S. Kebekus, E. Kruus, E. Leitl, D. Mathog, C. Morley, P. Murray-Rust, A. Nicholls, S. Patchkovskii, S. Reith, L. Richard, R. Sayle, A. Shah, M. Stahl, B. Tolbert, P. Walters, P. Wolinski, J. Wegner, Open Babel, 2005.

[11] G. Mazzoccanti, R. Franzini, A. Ciogli, M. Pierini, F. Buonsenso, C. Faggi, A. Francioso, S. Manetto, C. Villani, "Theoretically Predicted and Experimentally Detected Chirality of Dibenzocyclooctynes and Their Triazole Adducts with Azides" *J. Org. Chem.* **88**, (2023): 15097-15105.

[12] D. A. Case, H. M. Aktulga, K. Belfon, D. S. Cerutti, G. A. Cisneros, V. W. D. Cruzeiro, N. Forouzesh, T. J. Giese, A. W. Götz, H. Gohlke, "AmberTools" *J. Chem. Inf. Model.* **63**, (2023): 6183-6191.

[13] D. A. Case, D. S. Cerutti, V. W. D. Cruzeiro, T. A. Darden, R. E. Duke, M. Ghazimirsaeed, G. M. Giambaşu, T. J. Giese, A. W. Götz, J. A. Harris, K. Kasavajhala, T.-S. Lee, Z. Li, C. Lin, J. Liu, Y. Miao, R. Salomon-Ferrrer, J. Shen, R. Snyder, J. Swails, R. C. Walker, J. Wang, X. Wu, J. Zeng, T. E. Cheatham Iii, D. R. Roe, A. Roitberg, C. Simmerling, D. M. York, M. C. Nagan, K. M. Merz, Jr.,

"Recent Developments in Amber Biomolecular Simulations" *J. Chem. Inf. Model.* **65**, (2025): 7835-7843.
[14] M. Zgarbová, J. Sponer, M. Otyepka, T. E. Cheatham III, R. Galindo-Murillo, P. Jurecka, "Refinement of the Sugar–Phosphate Backbone Torsion Beta for AMBER Force Fields Improves the Description of Z-and B-Dna" *J. Chem. Theory Comput.* **11**, (2015): 5723-5736.
[15] I. S. Joung, T. E. Cheatham III, "Determination of Alkali and Halide Monovalent Ion Parameters for Use in Explicitly Solvated Biomolecular Simulations" *J. Phys. Chem. B* **112**, (2008): 9020-9041.
[16] J. Wang, W. Wang, P. A. Kollman, D. A. Case, "Automatic atom type and bond type perception in molecular mechanical calculations" *J. Mol. Graph. Model.* **25**, (2006): 247-260.
[17] A. Jakalian, B. L. Bush, D. B. Jack, C. I. Bayly, "Fast, efficient generation of high-quality atomic charges. AM1-BCC model: I. Method" *J. Comput. Chem.* **21**, (2000): 132-146.
[18] J. Wang, R. M. Wolf, J. W. Caldwell, P. A. Kollman, D. A. Case, "Development and Testing of a General Amber Force Field" *J. Comput. Chem.* **25**, (2004): 1157-1174.
[19] T. Darden, D. York, L. Pedersen, "Particle mesh Ewald: An N log (N) method for Ewald sums in large systems" *J. Chem. Phys.* **98**, (1993): 10089-10089.
[20] U. Essmann, L. Perera, M. L. Berkowitz, T. Darden, H. Lee, L. G. Pedersen, "A smooth particle mesh Ewald method" *J. Chem. Phys.* **103**, (1995): 8577-8593.
[21] J.-P. Ryckaert, G. Ciccotti, H. J. Berendsen, "Numerical integration of the cartesian equations of motion of a system with constraints: molecular dynamics of n-alkanes" *J. Comput. Phys.* **23**, (1977): 327-341.
[22] H. J. Berendsen, J. v. Postma, W. F. Van Gunsteren, A. DiNola, J. R. Haak, "Molecular dynamics with coupling to an external bath" *J. Chem. Phys.* **81**, (1984): 3684-3690.
[23] R. J. Loncharich, B. R. Brooks, R. W. Pastor, "Langevin dynamics of peptides: The frictional dependence of isomerization rates of N-acetylalanyl-N′-methylamide" *Biopolymers* **32**, (1992): 523-535.
[24] T. J. Giese, R. Snyder, Z. Piskulich, G. P. Barletta, S. Zhang, E. McCarthy, Ş. Ekesan, D. M. York, "FE-ToolKit: A Versatile Software Suite for Analysis of High-Dimensional Free Energy Surfaces and Alchemical Free Energy Networks" *J. Chem. Inf. Model.* **65**, (2025): 5273-5279.
[25] M. R. Shirts, J. D. Chodera, "Statistically Optimal Analysis of Samples From Multiple Equilibrium States" *J. Chem. Phys.* **129**, (2008).
[26] X.-J. Lu, H. J. Bussemaker, W. K. Olson, "DSSR: an integrated software tool for dissecting the spatial structure of RNA" *Nucleic Acids Res.* **43**, (2015): e142-e142.
[27] W.-Z. Wang, L.-B. Huang, S.-P. Zheng, E. Moulin, O. Gavat, M. Barboiu, N. Giuseppone, "Light-driven molecular motors boost the selective transport of alkali metal ions through phospholipid bilayers" *J. Am. Chem. Soc.* **143**, (2021): 15653-15660.

[28] M. Hajji, M. Cariello, C. Gilroy, M. Kartau, C. D. Syme, A. Karimullah, N. Gadegaard, A. Malfait, P. Woisel, G. Cooke, "Chiral quantum metamaterial for hypersensitive biomolecule detection" *ACS nano* **15**, (2021): 19905-19916.
[29] M. M. Pollard, M. K. ter Wiel, R. A. van Delden, J. Vicario, N. Koumura, C. R. van den Brom, A. Meetsma, B. L. Feringa, "Light-driven rotary molecular motors on gold nanoparticles" *Chem. Eur. J.* **14**, (2008): 11610-11622.
[30] D. J. van Dijken, J. Chen, M. C. Stuart, L. Hou, B. L. Feringa, "Amphiphilic molecular motors for responsive aggregation in water" *J. Am. Chem. Soc* **138**, (2016): 660-669.
[31] S. Ida, M. Ouchi, M. Sawamoto, "Living cationic polymerization of an azide-containing vinyl ether toward addressable functionalization of polymers" *J. Polym. Sci. A Polym. Chem.* **48**, (2010): 1449-1455.
[32] Q. Zhao, J. Jiang, W. Zhao, S.-H. Li, W. Mi, C. Zhang, "Truxone-Based Conductive Metal–Organic Frameworks for the Oxygen Reductive Reaction" *J. Phys. Chem. C* **125**, (2021): 12690-12698.
[33] M. Kathan, S. Crespi, N. O. Thiel, D. L. Stares, D. Morsa, J. de Boer, G. Pacella, T. van den Enk, P. Kobauri, G. Portale, C. A. Schalley, B. L. Feringa, "A light-fuelled nanoratchet shifts a coupled chemical equilibrium" *Nat. Nanotechnol.* **17**, (2022): 159-165.
[34] J. Conyard, A. Cnossen, W. R. Browne, B. L. Feringa, S. R. Meech, "Chemically Optimizing Operational Efficiency of Molecular Rotary Motors" *J. Am. Chem. Soc.* **136**, (2014): 9692-9700.
[35] A. Cnossen, J. C. M. Kistemaker, T. Kojima, B. L. Feringa, "Structural Dynamics of Overcrowded Alkene-Based Molecular Motors during Thermal Isomerization" *J. Org. Chem.* **79**, (2014): 927-935.
[36] C. Gao, A. Vargas Jentzsch, E. Moulin, N. Giuseppone, "Light-Driven Molecular Whirligig" *J. Am. Chem. Soc.* **144**, (2022): 9845-9852.
[37] M. Leng, G. Felsenfeld, "A study of polyadenylic acid at neutral pH" *J. Mol. Biol.* **15**, (1966): 455-466.
[38] H. Eisenberg, G. Felsenfeld, "Studies of the temperature-dependent conformation and phase separation of polyriboadenylic acid solutions at neutral pH" *J. Mol. Biol.* **30**, (1967): 17-37.
